# The best strategy for HCC patients at each BCLC stage: a network meta-analysis of observational studies

**DOI:** 10.18632/oncotarget.14668

**Published:** 2017-01-15

**Authors:** Lei Chang, Yitao Wang, Jibo Zhang, Tao Guo

**Affiliations:** ^1^ Department of General Surgery, Zhongnan Hospital of Wuhan University, Wuhan, P.R. China; ^2^ Department of Neurosurgery, Zhongnan Hospital of Wuhan University, Wuhan, P.R. China

**Keywords:** hepatocellular carcinoma, BCLC stages, network meta-analysis

## Abstract

**Background and Aims:**

Currently, the Barcelona Clinic Liver Cancer staging system remains huge controversies in the management of hepatocellular carcinoma. To determine the best therapeutic strategy for patients at each stage, we conducted a network meta-analysis and aimed to provide a new treatment concept.

**Materials and Methods:**

PubMed, Embase and Cochrane Library database were searched for observational studies up to August 31, 2016. We extracted data on overall survival rate from studies that compared various strategies for use with patients at different stages. Network meta-analysis was conducted by evaluating the different overall survival rate of each stage. Cumulative probability value was utilized to rank the strategies under examination. A node-splitting model was employed to assess consistency and inconsistency.

**Results:**

A total of 198 observational studies were included in the network meta-analysis with a focus on Stages 0-D. By comparing the overall survival rate of each stage, the results revealed that liver transplantation and liver transplantation plus transcatheter arterial chemoembolization were the best options for patients with Stages 0 and A. The applications of surgical resection plus transcatheter arterial chemoembolization and surgical resection plus sorafenib were the best strategies for Stages B and C. For Stage D, whole net connection could not be established, but intra-arterial infusion chemotherapy and liver transplantation could be potential primary options.

**Conclusions:**

The existing therapeutic flowchart needs to be updated. Potential best strategies relating to all stages were identified and should be used as references for clinical treatments.

## INTRODUCTION

Hepatocellular carcinoma (HCC) is one of the most common cancers and causes of cancer death worldwide. Despite recent treatment advances, there are limited options that provide an opportunity for a cure for this disease [1–2]. In addition, a substantial proportion of patients have poor liver reserve and/or compromised portal vein flow; consequently, untreated unresectable HCC has a poor prognosis [3]. So the management of patients with HCC is complicated and keeps developing.

The Barcelona Clinic Liver Cancer (BCLC) staging system, which establishes the prognosis and best treatment strategy for patients at different stages, has been used widely across the world since it was developed [4]. After two modifications [5–6], it became the standard specification for treatment of patients at different stages. The BCLC system is beneficial for HCC patients. Its obvious advantage is that it provides therapy options for each patient at different stages. Therefore, it is a complete management model that is worthy of strong recommendation. However, some scholars have stated that the BCLC therapeutic flowchart is too conservative and have recommended that it could be replaced [7]. However, until now, there was no systematic comprehensive quantitative evidence to use to determine the best therapy strategy for patients at each BCLC stage. Therefore, it is important to establish a new HCC management system to provide the best therapeutic strategies to each patient.

In the last 2 decades, many studies about different therapeutic methods were published in different nations. Various treatment strategies were found to have different advantages. The argument about which one was best still remained. Among these studies, at the same level of objective evidence, observational studies were conducted to examine almost all of the treatments. Based on these facts, we performed a network meta-analysis to determine the best therapeutic strategy for patients at each BCLC stage. Most importantly, the objective of this study was to provide a new treatment concept that extends beyond the specific therapeutic strategy itself.

## RESULTS

### Study characteristics and quality

**Figure 1:**
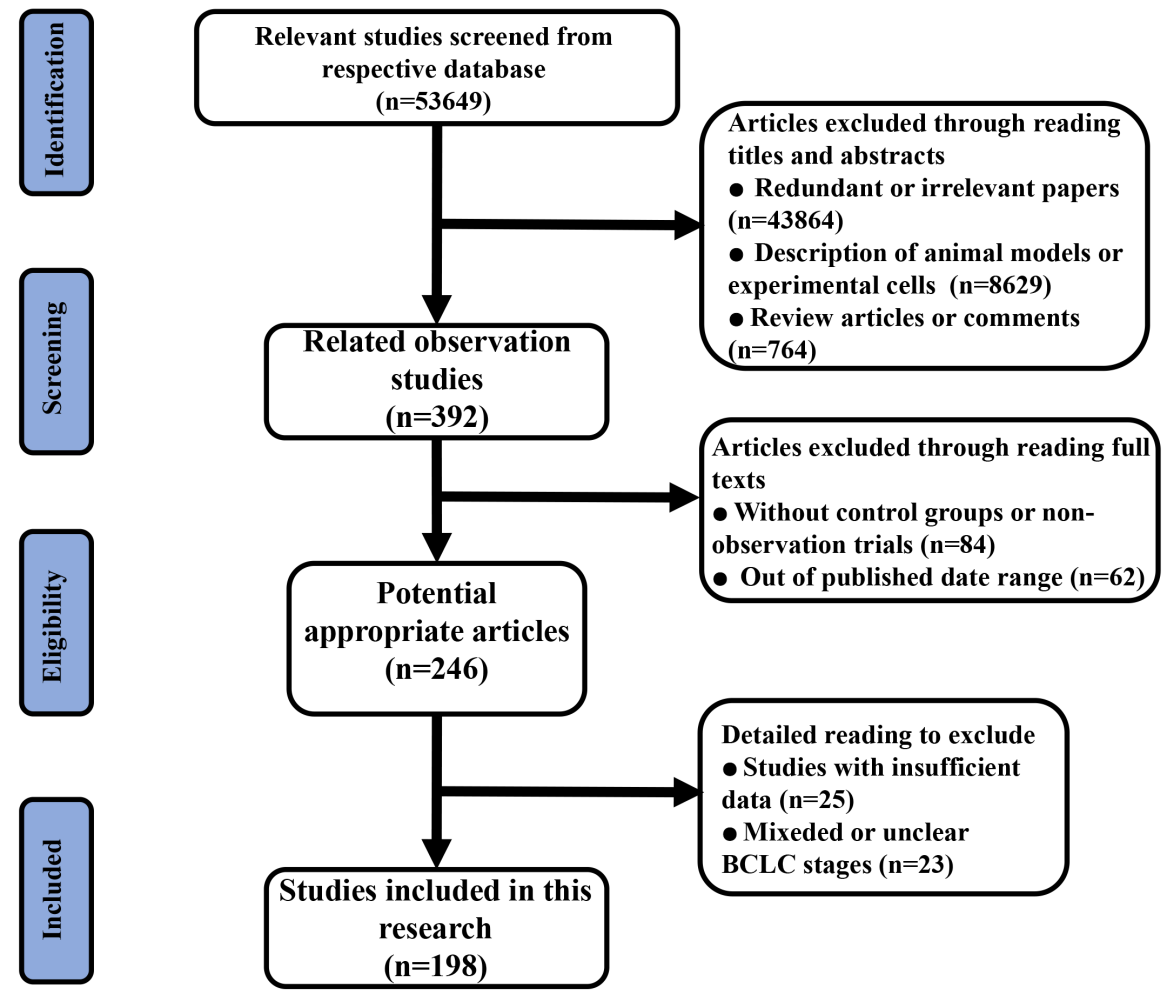
Flow diagram of the process of (and the reasons for) including and excluding studies for this meta-analysis

After identifying 53,649 articles (Figure 1), 198 studies ultimately satisfied the inclusion criteria (Supplementary Table 1). 16 of them reported information on 2 or more stages. The studies were performed in China (*n* = 70), Italy (*n* = 15), Japan (*n* = 32), Taiwan (*n* = 17), Korea (*n* = 33), the USA (*n* = 10), the UK (*n* = 1), Hong Kong (*n* = 5), Australia (*n* = 1), France (*n* = 3), Germany (*n* = 4), Switzerland (*n* = 1), Spain (*n* = 3), Austria (*n* = 2), and Romania (*n* = 1). First-line or potential first-line treatment methods for patients at different BCLC stages included liver transplantation (LT), microwave ablation (MWA), percutaneous ethanol injection (PEI), radiofrequency ablation (RFA), radiation therapy (RT), supportive care (SC), surgical resection (SR), transcatheter arterial chemoembolization (TACE), high-intensity focused ultrasound ablation (HIFU), percutaneous acetic acid injection (PAI), percutaneous cryosurgery (PC), sorafenib, transcatheter arterial chemotherapy (TAC), transarterial radioembolization (TARE), intra-arterial infusion chemotherapy (HAC), systemic chemotherapy (SCT), portal vein chemotherapy (PVC), portal vein embolization (PVE), and their combinations. Of these 198 cohort studies, we calculated their scores according to the cohort study checklist. None had scores from 0-3, 51 had scores from 4-6, and 137 had scores from 7-9 (Supplementary Table 2).

### Network meta-analysis of different overall survival rates associated with respective BCLC stages

**Figure 2:**
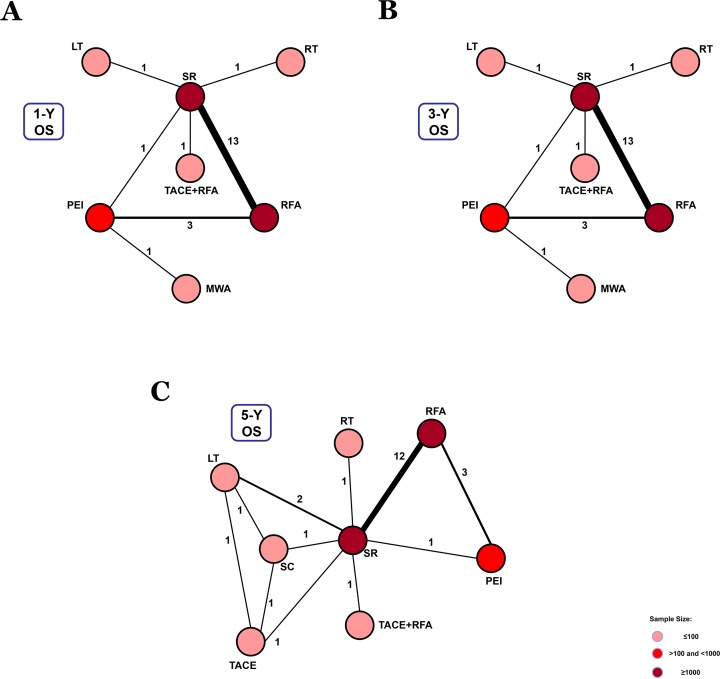
Network connections of included studies for BCLC Stage 0 **A**. Data for 1-year overall survival rate. **B**. Data for 3-year overall survival rate. **C**. Data for 5-year overall survival rate.

For BCLC Stage 0, 21 studies reported data on 1-, 3-, and 5-year overall survival rates (Supplementary Table 1). 7 treatment methods were included in the 1- and 3-year overall survival analysis (Figure 2A, Figure 2B). We found that TACE+RFA and LT were the best therapeutic strategies for 1-year and 3-year overall survival (*P* = 0.43 and *P* = 0.52, respectively) (Figure 5A). In addition, for the 5-year overall survival analysis, 8 treatment methods (Figure 2C) were included. It was determined that LT was the best strategy (*P* = 0.71) (Figure 5A) (Supplementary Table 4).

**Figure 3:**
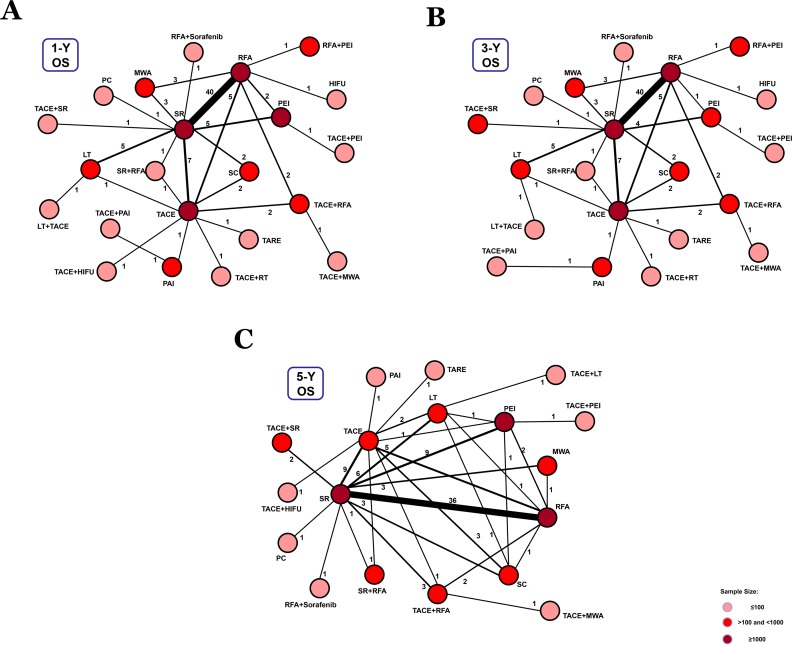
Network connections of included studies for BCLC Stage A **A**. Data for 1-year overall survival rate. **B**. Data for 3-year overall survival rate. **C**. Data for 5-year overall survival rate.

In 91 studies, 1-, 3-, and 5-year overall survival rates for BCLC Stage A were reported in association with 22, 22, and 18 therapeutic methods, respectively (Figure 3). We conducted a network meta-analysis for different overall survival by establishing 3 network connections. The results showed that TACE+PAI seemed to be the best option in terms of early survival rate (1- year and 3-year) (*P* = 0.56, *P* = 0.40, respectively) (Figure 5B). TACE+LT was superior to other methods in terms of late survival rate (5-year) (*P* = 0.42) (Figure 5B) (Supplementary Table 4).

**Figure 4:**
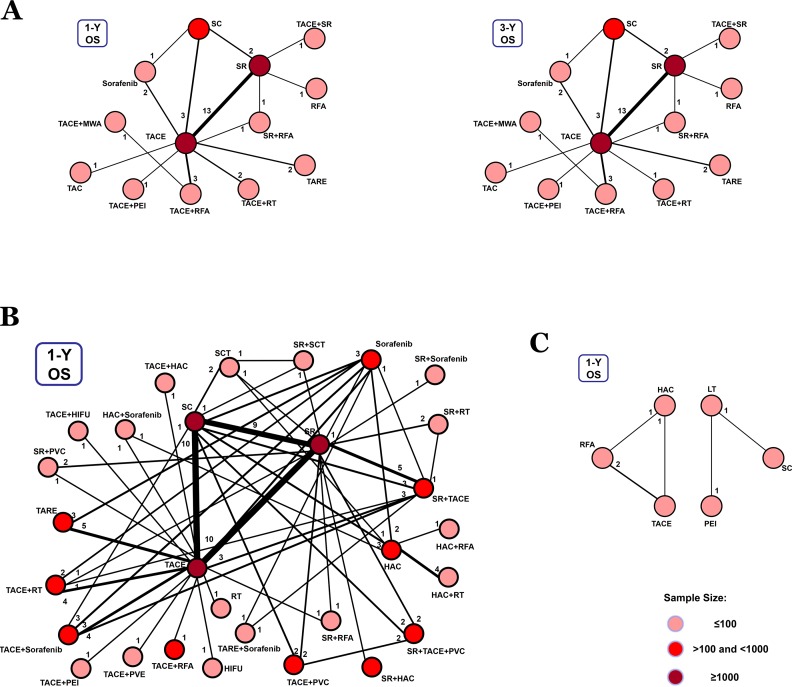
Network connections of included studies for BCLC Stages B, C, and D **A**. Data for 1-year (left panel) and 3-year (right panel) overall survival rates for BCLC Stage B. **B**. Data for 1-year overall survival rate for BCLC Stage C. **C**. Data for 1-year overall survival rate for BCLC Stage D.

**Figure 5:**
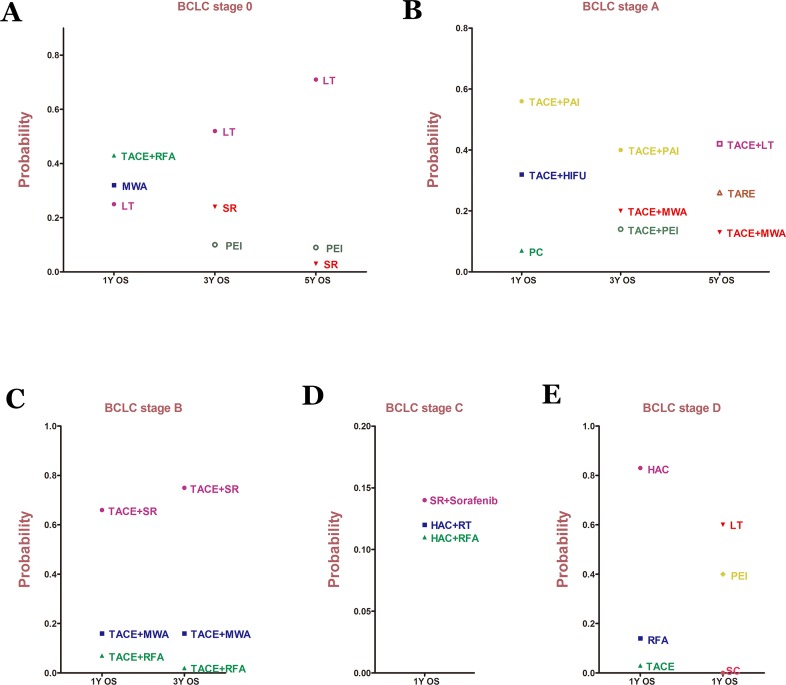
Probability of different therapy strategies as measured by the included outcomes for BCLC **A**. Stage 0; **B**. Stage A; **C**. Stage B; **D**. Stage C; **E**. Stage D. OS: overall survival.

For BCLC Stage B, 29 studies presented 1-year and 3-year overall survival rates associated with 13 therapeutic methods (Figure 4A). After a network meta-analysis of 1-year and 3-year survival rates (Supplementary Table 4), we found that TACE+SR was the best therapeutic strategy for patients with primary HCC in BCLC Stage B in terms of both 1-year and 3-year overall survival rate (*P* = 0.66 and *P* = 0.75, respectively) (Figure 5C).

Only the 1-year overall survival rate that was presented in 74 articles was included for analysis of data from patients with BCLC Stage C. The connection was established with 28 first-line or potential first-line therapeutic strategies (Figure 4B). After pooled estimation (Supplementary Table 4), we found that SR+sorafenib was the most effective therapy for patients with BCLC Stage C in terms of 1-year overall survival rate (*P* = 0.14) (Figure 5D).

For BCLC Stage D patients, we only compared the 1-year overall survival rate. However, the whole network connection could not be established. A total of 6 study arms from 4 studies were divided into 2 separate net connections (Figure 4C). Based on the results of the meta-analysis, we found that HAC and LT may be superior to other therapeutic methods (Supplementary Table 4) (Figure 5E), but the related best strategy was unclear.

### Consistency and convergence analysis

In this research, node-splitting models were developed to assess inconsistency by testing the difference between the direct and indirect effects. The goal was to determine whether direct and indirect evidence on a specific node (the split node) were in agreement. After constructing the node-splitting models, we found that no significant inconsistencies existed (Supplementary Table 5). The result of the consistency model was reliable. Moreover, all PSRF values of the different parameters were limited to 1, which demonstrated good convergence and efficiency.

## DISCUSSION

Staging of patients with HCC is important both for the prognosis and the decision about the treatment [8]. To achieve more accuracy in HCC management, various staging systems for use with HCC have appeared [4–6, 9–13]. Among them, the BCLC staging system has become widely accepted in clinical practice. Although the BCLC staging system is innovative and includes several aspects of HCC biology and underlying liver disease, its general application remains a topic of ongoing discussion, especially in cases of potentially resectable lesions [14]. Moreover, surgical resection for patients in early BCLC stages was treated more invasively than local therapy and did not reveal significant advantages for individual patients [15]. For advanced-stage patients, surgical resection and combined therapy are also recommended [16–17]. Several systematic reviews and meta-analysis tried to explore better HCC management [18–19]. However, so far, there has not been quantitative statistical evidence and systematic objective judgment to guide this discussion.

In the last 2 decades, some RCTs and many observational studies comparing different therapeutic strategies with respect to BCLC stage have been published. Compared to observational studies, RCTs present stronger evidence for meta-analysis. However, appropriate published RCTs in this field were insufficient for analysis (especially for BCLC Stages 0, B, and D). In addition, in objective situations, some therapeutic strategies such as live transplantation cannot be performed using RCTs. In contrast, many observational studies including sufficient respective first-line or potential first-line therapies for each BCLC stage have been reported. Based on these facts, we performed a network meta-analysis to determine the best therapeutic strategy for each BCLC stage, according to the overall survival rate.

Under the premise of good convergence and consistency, results from 198 observational studies were analyzed. According to the results, for BCLC Stage 0, although TACE+RFA had the best effect on 1-year overall survival, LT was superior to other methods in terms of 3-year and 5-year overall survival (Figure 5A). For BCLC Stage A, TACE+LT had the best effect on long-term overall survival rate, even though TACE+PAI may be the best treatment for early survival (Figure 5B). LT may be the best option for patients with early-stage HCC (0~A). For patients at Stage B, TACE+SR showed an obvious benefit compared to other treatments. Therefore, we could see that surgical resection was not an absolutely forbidden treatment. In contrast, it seemed to be the best strategy at this stage. We also found that SR+sorafenib had the best effect among patients at Stage C, which meant that both SR could be used in this stage and sorafenib was needed. Finally, in the analysis of Stage D, 2 separate net connections were established because the whole network could not be conducted. However, LT and HAC were identified to be better than other methods, but it was not clear which was the best treatment.

**Figure 6:**
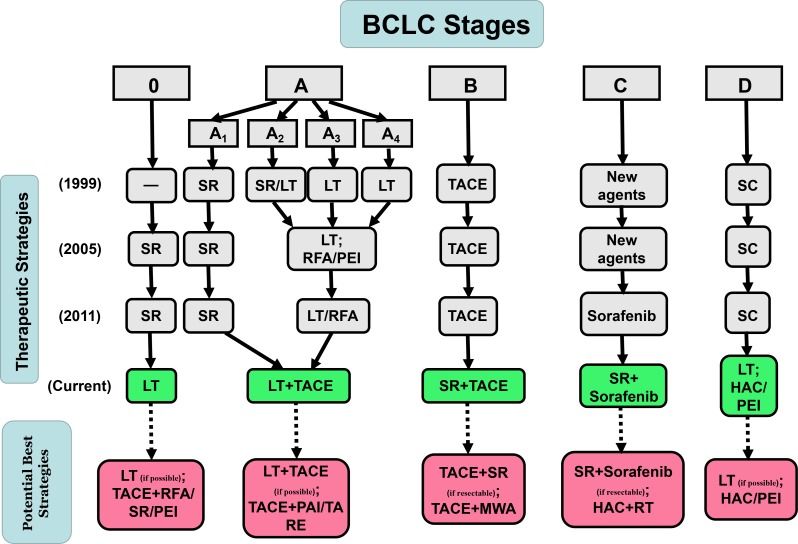
Previous version of and updates to BCLC Therapeutic Flowchart

As previously mentioned, *via* quantitative analysis of the overall survival rate, we examined the best therapeutic strategies for each BCLC stage and updated the BCLC therapeutic flowchart (Figure 6). And we still need to make them more specific and complete to determine the potential best strategies based on these objective data. First, as shown in Figure 5, if possible, LT was the best option for patients at Stages 0, A, D, but not patients at Stages B and C. It was likely that patients at Stages B (large or multiple) and C (vascular invasion or metastasis) had a higher risk of HCC recurrence and LT could not ameliorate this condition. In addition, a combination with TACE was suitable for patients at early stages (0, A, B). However, as stage progressed, TACE was no longer appropriate, possibly because of its revealed harmful effects. Instead, a combination with HAC or HAC alone was superior to other methods in advanced stages (C, D). This finding may be related to the metabolism of tumors in the hypoxic environment [20–21]. Based on these results, it is possible to conclude that LT (if possible) was the best option for patients at Stage 0, whereas TACE+RFA, SR, PEI might be potential best options for patients at Stage 0 (if LT is not possible). For patients at Stage A, LT+TACE (if possible) was the most effective treatment. TACE+PAI or TARE could also be potential best options (if LT is not possible). Furthermore, with resectable HCC, SR+TACE and SR+sorafenib were obviously the best options for patients at Stages B and C, respectively. With unresectable HCC, TACE+WMA and HAC+RT were potential best treatments for each of the 2 stages. Finally, LT (if possible) and HAC or PEI (if LT is not possible) could be the first considerations for patients at Stage D (Figure 6).

For the first time, we performed a quantitative network meta-analysis of overall survival rates associated with different BCLC stages to determine the best therapeutic treatments for each stage. Based on objective results and in-depth analysis, we reached preliminary conclusions. Findings demonstrated that the previous BCLC therapeutic flowchart had a certain reference value but was too conservative. Moreover, at some certain stages, combined therapy should be recommended to replace single therapy. In addition, we determined that the therapeutic flowchart should still be updated in the future as therapeutic methods evolve. On the other hand, although we had demonstrated the data consistency and good convergence, due to the limitations of observational studies, inclusion of only observational studies may lead to potential biases.

Despite the existence of several potential limitations, we updated and addressed the HCC strategies and potential best treatments for the BCLC staging system based on objective data. We concluded that the current therapeutic flowchart should be viewed as a reference for clinical treatment.

## MATERIALS AND METHODS

### Data sources and search strategy

This review was conducted using a predefined protocol and in accordance with PRISMA and MOOSE guidelines [22–23]. Global databases (PubMed, EMBASE, and the Cochrane Library database) from September 1, 1997 to August 31, 2016. We did not apply any language, publication date, or publication status restrictions. For a more comprehensive and inclusive review, we conducted an initial literature search of respective databases using only a few expressions, such as “hepatocellular carcinoma (or HCC and liver cancer)” and “overall survival.” Then, we expanded the search terms to include relevant topics to avoid neglecting eligible studies. All abstracts that were available in English and other languages were reviewed. We referred to the full text when necessary to clarify eligibility status. We limited our attention to the various first-line or potential first-line therapy methods for HCC.

### Study selection and eligibility criteria

The studies included in our meta-analysis satisfied all the following criteria: (1) retrospective or prospective cohort study; (2) primary HCC was clearly diagnosed, and the treatment method was the only intervention in the study; (3) the BCLC stage was clearly described for each treatment method; (4) outcome information, including the overall survival rate, was provided (1-, 3-, and 5-year follow-up for BCLC Stages 0-A; 1- and 3-year for Stage B; 1-year for Stages C and D);

The exclusion criteria eliminated studies with the following characteristics: (1) non-comparative study; (2) mixed BCLC stages or treatment methods; (3) outcome information was not provided or was insufficient; (4) inclusion of patients with recurrent HCC; (5) limited to animals or cells; (6) reviews, study protocols, comments, or case reports; (7) published outside of the date range.

### Data extraction and quality assessment

Two investigators (Chang L, Wang YT) independently reviewed the full manuscripts of eligible studies and entered the extracted information, including publication data (the first author's name, year of publication, and country of the population under examination), treatment methods, number of patients, and overall survival rates, into an electronic database. Any discrepancies in the extraction of data were resolved by the primary investigator (Guo T). We collected the survival data after propensity score matching if it was performed. If both survival rates and Kaplan-Meier curves were presented, only the survival rates were documented. If only Kaplan-Meier curves were reported, we extracted the cumulative 1-, 3-, and 5-year survival rates using the distance tool in the measurements menu of the Foxit PDF Reader software (Foxit Cooperation, California, USA). This software or method is suitable for the measurement of survival rate in meta-analysis [24]. Two reviewers (Chang L, Zhang JB) independently assessed the quality of each study included in the database.

The Newcastle-Ottawa Quality Assessment Scale was selected to assess the methodological quality of the prospective or retrospective cohort studies [25]. Three major components of each study were examined: patient selection; the comparability of the intervention and the observation groups; and outcome assessment (Supplementary Table 2, Supplementary Table 3). Controversial items were discussed with the primary investigator (Tao G) before final consensus was reached.

### Statistical analysis

In this research, we paid close attention to the overall survival rates of different interventions for primary HCC with respect to BCLC stage. It was necessary to make comparisons across all therapy strategies *via* a comprehensive network meta-analysis based on Bayesian theorem. This analysis can be considered to be an extension of the traditional pairwise meta-analysis, as it incorporates both direct and indirect information through a common comparator to obtain estimates of relative effects *via* multiple comparisons [26–27].

We evaluated consistency by combining the quantitative estimates from the indirect comparisons, according to the experimental design and primary outcome of the included studies. If there was no evidence of a relevant inconsistency, a consistency model could be used to draw conclusions about the relative effect of the included interventions. In addition, cumulative P values were calculated to reveal the priorities of each treatment. So a relevant rank probability plot could present the best therapeutic measure. Meanwhile, node-splitting analysis was also performed to investigate whether a statistically significant inconsistency existed when *P* > 0.05. If there was any evidence of a relevant inconsistency, related confounders need to be analyzed. Convergence was assessed to calculate the potential scale reduction factor (PSRF), the values of which were limited to 1.

Data for 1-, 3-, and 5-year overall survival rates in each study with respect to BCLC stage was recorded and added to the pooled estimation using network meta-analysis. For certain BCLC stages, if the included intervention connections could not be established as a whole net, the results could be revealed as separate net connections or direct comparisons and be described together comprehensively.

The automated software Aggregate Data Drug Information System (ADDIS, version 1.16, GZ Groningen, Netherlands) was used for the network pooled estimation and node-splitting analysis. Related plots were drawn using GraphPad Prism (version 5.0, GraphPad Software, La Jolla, USA).

## SUPPLEMENTARY TABLES


