# Supplementary material for: The best strategy for HCC patients at each BCLC stage: a network meta-analysis of observational studies
Source: Oncotarget. 2017 Jan 15;8(12):20418–27. doi: 10.18632/oncotarget.14668 (PMC5386773; doi:10.18632/oncotarget.14668)
Supplement: Supplementary file 1 [file oncotarget-08-20418-s001.pdf]

Supplementary Table S1. Characteristics of the included trials.

Stages	Authors	Pub. Date	Country	Study Arms	Intervention	Sample Size	Available Data
**BCLC Stage 0**							
	Dai [1]	2014	China	2	LT vs. SR	13 vs. 25	1y, 3y, 5y
	Farinati [2]	2009	Italy	4	LT vs. SR vs. TACE vs. SC	3 vs. 8 vs. 17 vs. 3	5y
	Harada [3]	2016	Japan	2	SR vs. RFA	35 vs. 28	1y, 3y, 5y
	Hasegawa [4]	2012	Japan	3	SR vs. RFA vs. PEI	785 vs. 1241 vs. 534	1y, 3y, 5y
	Horigome [5]	1999	Japan	2	PEI vs. MWA	18 vs. 24	1y, 3y
	HUNG [6]	2011	Taiwan	2	SR vs. RFA	50 vs. 66	1y, 3y, 5y
	Imai [7]	2013	Japan	2	SR vs. RFA	38 vs. 51	1y, 3y, 5y
	Kim [8]	2015	Korea	2	SR vs. RFA	152 vs. 152	1y, 3y, 5y
	Lin [9]	2013	China	2	PEI vs. RFA	242 vs. 284	1y, 3y, 5y
	Liu [10]	2015	Taiwan	2	SR vs. RFA	109 vs. 128	1y, 3y, 5y
	Livraghi [11]	2008	Italy	2	SR vs. RFA	100 vs. 118	3y, 5y
	Pompili [12]	2013	Italy	2	SR vs. RFA	99 vs. 109	1y, 3y
	Pompili [13]	2015	Italy	2	PEI vs. RFA	81 vs. 81	1y, 3y, 5y
	Santambrogio [14]	2016	Italy	2	SR vs. RFA	76 vs. 76	1y, 3y, 5y
	Takayama [15]	2010	Japan	2	SR vs. RFA	1235 vs. 1315	1y
	Takuma [16]	2013	Japan	2	TACE+RFA vs. SR	59 vs. 52	1y, 3y, 5y
	TASHIRO [17]	2011	Japan	2	SR vs. RFA	53 vs. 41	1y, 3y, 5y
	Wahl [18]	2015	USA	2	RT vs. RFA	38 vs. 133	1y, 3y, 5y
	Wang [19]	2012	Taiwan	2	SR vs. RFA	52 vs. 91	1y, 3y, 5y
	Zhen [20]	2012	China	2	SR vs. RFA	29 vs. 37	1y, 3y, 5y
	Zhou [21]	2013	China	2	SR vs. RFA	21 vs. 31	1y, 3y, 5y
**BCLC Stage A**							
	Abu-Hilal [22]	2008	UK	2	RFA vs. SR	34 vs. 34	1y, 5y
	BABA [23]	2010	Japan	2	SR vs. TACE	114 vs. 68	5y
	Bu [24]	2015	China	2	RFA vs. SR	46 vs. 42	1y, 3y, 5y
	Chen [25]	2010	China	2	SR+TACE vs. SR	797 vs. 314	3y, 5y
	Chen [26]	2016	China	2	TACE vs. RFA	103 vs. 103	1y, 3y, 5y
	Cheung [27]	2012	HK	2	HIFU vs. RFA	41 vs. 56	1y, 3y
	Cho [28]	2007	Korea	2	SR vs. PEI	116 vs. 116	1y, 3y, 5y
	Daniele [29]	2003	Italy	2	SR vs. PEI	17 vs. 65	1y, 3y, 5y
	Desiderio [30]	2013	Italy	2	RFA vs. SR	44 vs. 52	1y, 3y, 5y
	Ding [31]	2013	China	2	RFA vs. MWA	85 vs. 113	1y, 3y
	Farinati [2]	2009	Italy	6	LT vs. RFA vs. SR vs. PEI vs.TACE vs. SC	11 vs. 58 vs. 75 vs. 106 vs. 128 vs. 62	5y
	Gerunda [32]	2000	Italy	2	TACE+SR vs. SR	20 vs. 20	1y, 5y

Ginsburg [33]	2015	USA	2	TACE+RFA vs. TACE+MWA	28 vs. 36	1y, 3y, 5y
GORY [34]	2015	Australia	2	RFA vs. SR	96 vs. 52	3y, 5y
Gournay [35]	2002	France	2	SR vs. PEI	50 vs. 55	1y, 3y, 5y
Graham [36]	2013	USA	2	LT vs. SR	344 vs. 560	5y
Graham [37]	2013	USA	2	LT vs. SR	608 vs. 557	1y, 3y
Guan [38]	2016	China	2	RFA vs. SR	102 vs. 92	1y, 3y, 5y
Guo [39]	2010	China	2	RFA vs. SR	86 vs. 73	1y, 3y, 5y
Guo [40]	2014	China	2	SR vs. TACE	152 vs. 152	1y, 3y, 5y
Guo [41]	2014	China	2	SR vs. TACE	84 vs. 42	1y, 3y, 5y
Harada [3]	2016	Japan	2	SR vs. RFA	46 vs. 12	1y, 3y, 5y
HARADA [42]	2016	Japan	2	RFA vs. SR	20 vs. 68	1y, 3y, 5y
Hasegawa [43]	2008	Japan	3	SR vs. RFA vs. PEI	2857 vs. 3022 vs. 1306	1y
Hasegawa [4]	2012	Japan	3	SR vs. RFA vs. PEI	2565 vs. 1265 vs. 329	1y, 3y, 5y
Hekele [44]	2009	Austria	2	TACE vs. SC	5 vs. 17	1y, 3y, 5y
Hiraoka [45]	2008	Japan	2	RFA vs. SR	105 vs. 59	1y, 3y, 5y
Ho [46]	2009	Taiwan	3	TACE vs. SR vs. SC	80 vs. 97 vs. 31	1y, 3y, 5y
Hocquelet [47]	2015	France	2	RFA vs. SR	140 vs. 37	3y, 5y
Honda [48]	2013	Japan	2	TACE+RT vs. TACE	30 vs. 38	1y, 3y
Hong [49]	2005	Korea	2	RFA vs. SR	55 vs. 93	1y, 3y
Hsu [50]	2012	Taiwan	2	TACE vs. SR	73 VS. 112	1y, 3y, 5y
Huang [51]	2011	China	2	RFA vs. SR	212 vs. 311	1y, 3y, 5y
Huang [52]	2014	China	2	RFA vs. SR	121 vs. 225	1y, 3y
HUNG [6]	2011	Taiwan	2	RFA vs. SR	190 vs. 229	1y, 3y, 5y
Huo [53]	2003	Taiwan	2	TACE+PAI vs. PAI	53 vs. 55	1y, 3y
HUO [54]	2004	Taiwan	2	TACE vs. PAI	113 vs. 68	1y, 3y, 5y
Hyun [55]	2015	Korea	2	TACE+RFA vs. TACE	37 vs. 54	1y, 3y
Jiang [56]	2015	China	2	RFA vs. SR	160 vs. 224	1y, 3y, 5y
Kagawa [57]	2010	Japan	2	TACE+RFA vs. SR	62 vs. 55	1y, 3y, 5y
Kamada [58]	2002	Japan	2	TACE+PEI vs. PEI	32 vs. 37	1y, 3y, 5y
Kang [59]	2015	Korea	2	RFA vs. SR	438 vs. 142	1y, 3y, 5y
Kim [60]	2014	Korea	2	RFA vs. SR	67 vs. 66	1y, 3y
Kim [61]	2006	Korea	2	LT+TACE vs. LT	36 vs. 21	1y, 3y, 5y
Kim [62]	2011	Korea	2	TACE+RFA vs. RFA	83 vs. 231	1y, 3y, 5y
Kim [8]	2015	Korea	2	RFA vs. SR	150 vs. 209	1y, 3y, 5y
KIM [63]	2012	Korea	2	HIFU+TACE vs. TACE	25 vs. 32	1y, 5y
Kim [64]	2013	Korea	2	TACE+RFA vs. SR	37 vs. 47	1y, 3y
Kobayashi [65]	2009	Japan	2	RFA vs. SR	209 vs. 199	1y, 3y, 5y
Koniaris [66]	2011	USA	2	LT vs. SR	205 vs. 33	1y, 3y, 5y

Lai [67]	2013	HK	2	RFA vs. SR	31 vs. 80	1y, 3y, 5y
LAI [68]	2016	China	2	RFA vs. SR	33 vs. 61	3y
Lee [69]	2016	HK	2	RFA vs. MWA	26 vs. 10	1y, 3y, 5y
Lee [70]	2015	Taiwan	2	RFA vs. SR	147 vs. 147	1y, 3y, 5y
Lei [71]	2014	China	2	RFA vs. SR	156 vs. 133	1y, 3y, 5y
Li [72]	2016	China	2	SR vs. MWA	220 vs. 60	1y, 3y, 5y
LI [73]	2013	China	2	PC vs. SR	24 vs. 58	1y, 3y, 5y
Li [74]	2014	China	2	LT vs. SR	27 vs. 199	1y, 3y, 5y
Lida [75]	2013	Japan	2	RFA vs. SR	33 vs. 15	1y, 3y, 5y
Liu [76]	2014	China	2	RFA vs. TACE	424 vs. 282	1y, 3y, 5y
LUPO [77]	2007	Italy	2	RFA vs. SR	60 vs. 42	1y, 3y, 5y
Montorsi [78]	2005	Italy	2	RFA vs. SR	58 vs. 40	1y, 3y
Moon [79]	2007	Korea	2	LT vs. SR	17 vs. 100	1y, 3y, 5y
Moreno-Luna [80]	2013	USA	2	TARE vs. TACE	12 vs. 23	1y, 3y, 5y
Park [81]	2014	Korea	2	RFA vs. SR	57 vs. 129	1y, 3y, 5y
Peng [82]	2013	China	2	RFA vs. SR	63 vs. 60	1y, 3y, 5y
Pompili [12]	2013	Italy	2	RFA vs. SR	298 vs. 246	1y, 3y
Potretzke [83]	2016	USA	2	RFA vs. MWA	55 vs. 99	1y, 3y
Santambrogio [84]	2009	Italy	2	RFA vs. SR	74 vs. 78	1y, 3y, 5y
Shi [85]	2014	China	2	SR vs. MWA	107 vs. 117	1y, 3y, 5y
Shi [86]	2016	China	2	RFA vs. RFA+PEI	180 vs. 180	1y, 3y, 5y
Song [87]	2016	Korea	3	TACE+RFA vs. RFA vs. TACE	86 vs. 70 vs. 43	1y, 3y, 5y
Song [88]	2015	China	2	RFA vs. SR	78 vs. 78	1y, 3y
Sotiropoulos [89]	2009	Germany	3	LT vs. SR vs. TACE	26 vs. 26 vs. 10	1y, 3y, 5y
Takahashi [90]	2007	Japan	2	RFA vs. SR	171 vs. 53	5y
Takuma [16]	2013	Japan	2	TACE+RFA vs. SR	154 vs. 176	1y, 3y, 5y
TASHIRO [17]	2011	Japan	2	RFA vs. SR	20 vs. 99	1y, 3y, 5y
Tohme [91]	2012	USA	2	RFA vs. SR	60 vs. 50	1y, 3y, 5y
Ueno [92]	2009	Japan	2	RFA vs. SR	34 vs. 84	1y, 3y, 5y
Vitale [93]	2015	Italy	2	SR vs. SC	244 vs. 97	1y, 3y, 5y
Vitali [94]	2015	Switzerland	2	RFA vs. SR	60 vs. 45	1y, 3y, 5y
Vivarelli [95]	2004	Italy	2	RFA vs. SR	13 vs. 19	1y, 3y
Wang [19]	2012	Taiwan	2	RFA vs. SR	254 vs. 208	1y, 3y, 5y
Wong [96]	2012	Taiwan	2	RFA vs. SR	36 vs. 46	1y, 3y, 5y
Yamakado [97]	2008	Japan	2	TACE+RFA vs. SR	104 vs. 62	1y, 3y, 5y
YAN [98]	2016	China	2	SR vs. RFA+Sorafenib	60 vs. 60	1y, 3y, 5y
Yang [99]	2014	Korea	3	TACE vs. RFA vs. SR	66 vs. 79 vs. 52	1y, 3y, 5y
Yun [100]	2011	Korea	2	RFA vs. SR	255 vs. 215	1y, 3y, 5y

Zhang [101]	2015	China	2	SR vs. RFA	19 vs. 16	1y, 3y, 5y
Zhang [102]	2014	China	3	SR vs. SR+RFA vs. TACE	16 vs. 12 vs. 7	1y, 3y, 5y
Zhang [103]	2016	China	2	SR vs. MWA	122 vs. 68	1y, 3y, 5y
**BCLC Stage B**						
Arizumi [104]	2015	Japan	2	TACE vs. Sorafenib	24 vs. 32	1y, 3y
Azuma [105]	2015	Japan	2	TACE+RFA vs. TACE	20 vs. 39	1y, 3y
Ciria [106]	2015	Spain	2	SR vs. TACE	36 vs. 44	1y, 3y
Fouly [107]	2015	Germany	2	TACE vs. TARE	42 vs. 44	1y, 3y
Ginsburg [33]	2015	USA	2	TACE+RFA vs. TACE+MWA	8 vs. 10	1y, 3y
Guo [108]	2015	China	2	TACE+SR vs. SR	26 vs. 34	1y, 3y
Hekele [44]	2009	Austria	2	TACE vs. SC	9 vs. 22	1y, 3y
Ho [109]	2014	USA	2	SR vs. RFA	16 vs. 17	1y, 3y
Ho [46]	2009	Taiwan	3	TACE vs. SR vs. SC	163 vs. 122 vs. 70	1y, 3y
Jacob [110]	2015	USA	2	TACE+RT vs. TACE	37 vs. 123	1y, 3y
Jin [111]	2014	Korea	2	TACE vs. SR	61 vs. 62	1y, 3y
Ke [112]	2014	China	2	SR vs. TACE	53 vs. 53	1y, 3y
Kim [113]	2016	Korea	2	SR vs. TACE	52 vs. 225	1y, 3y
Lee [114]	2015	Korea	2	SR vs. TACE	59 vs. 59	1y, 3y
Lei [115]	2014	China	2	TACE vs. SR	490 vs. 433	1y, 3y
Lin [116]	2010	Taiwan	2	TACE vs. SR	78 vs. 93	1y, 3y
Liu [117]	2016	China	2	SR vs. TACE	156 vs. 156	1y, 3y
Lubienski [118]	2004	Germany	2	TACE+PEI vs. TACE	15 vs. 22	1y, 3y
Luo [119]	2011	China	2	TACE vs. SR	83 vs. 85	1y, 3y
Moreno-Luna [80]	2013	USA	2	TACE vs. TARE	13 vs. 34	1y, 3y, 5y
NISHIKAWA [120]	2014	Japan	2	TACE vs. TAC	145 vs. 81	1y, 3y
Pecorelli [121]	2016	Italy	3	TACE vs. Sorafenib vs. SC	233 vs. 18 vs. 21	1y, 3y
Shim [122]	2005	Korea	2	TACE+RT vs. TACE	38 vs. 35	1y
Vitale [93]	2015	Italy	2	SR vs. SC	264 vs. 213	1y, 3y
Yin [123]	2014	China	2	TACE+RFA vs. TACE	55 vs. 156	1y, 3y
Zhang [102]	2014	China	3	SR vs. SR+RFA vs. TACE	41 vs. 55 vs. 64	1y, 3y
Zhao [124]	2010	China	2	TACE vs. TACE+RFA	45 vs. 51	1y, 3y
Zhong [125]	2014	China	2	TACE vs. SR	135 vs. 257	1y, 3y
Zhong [126]	2013	China	2	SR vs. TACE	208 vs. 209	1y, 3y
**BCLC Stage C**						
Bai [127]	2016	China	3	SR vs. SR+TACE vs. SR+RT	51 vs. 31 vs. 10	1y
Chen [128]	2010	Taiwan	2	HAC+RT vs. HAC	10 vs. 14	1y
Chen [129]	2014	China	2	TACE+Sorafenib vs. TACE	21 vs. 23	1y
Cheng [130]	2005	China	4	SR+TACE vs. SR vs. TACE vs. SC	9 vs. 20 vs. 7 vs. 38 vs. 10	1y

Cheong [131]	2005	Korea	3	SC vs. SCT vs. HAC	24 vs. 25 vs. 53	1y
Cheung [132]	2014	HK	2	HIFU vs. TACE	26 vs. 52	1y
Cho [133]	2014	Korea	2	TACE+RT vs. Sorafenib	27 vs. 27	1y
Cho [134]	2016	Korea	2	TACE+HAC vs. TACE	31 vs. 50	1y
Choi [135]	2016	Korea	2	TARE vs. Sorafenib	32 vs. 31	1y
Choi [136]	2013	Korea	2	TACE+Sorafenib vs. TACE	96 vs. 96	1y
Chung [137]	2011	Korea	2	TACE vs. SC	83 vs. 42	1y
Edeline [138]	2015	France	2	RT vs. Sorafenib	34 vs. 117	1y
Fan [139]	2005	China	4	SC vs. TACE+PVC vs. SR vs. SR+TACE+PVC	18 vs. 53 vs. 24 vs. 84	1y
Fan [140]	2001	China	4	SC vs. TACE+PVC vs. SR vs. SR+TACE+PVC	18 vs. 18 vs. 74 vs. 29	1y
Fan [141]	2003	China	4	SC vs. SCT vs. SR. vs. SR+SCT	14 vs. 41 vs. 19 vs. 64	1y
Ha [142]	2016	Korea	2	Sorafenib vs. TACE+Sorafenib	293 vs. 129	1y
Hirooka [143]	2010	Japan	2	HAC+RFA vs. HAC	20 vs. 33	1y
Ho [46]	2009	Taiwan	3	TACE vs. SR vs. SC	38 vs. 48 vs. 134	1y
Hu [144]	2014	China	2	TACE+Sorafenib vs. TACE	82 vs. 164	1y
Hu [145]	2007	China	2	TACE+SR vs. SR	29 vs. 47	1y
Huang [146]	2016	China	2	TARE vs. TACE	70 vs. 140	1y
Jang [147]	2007	Korea	2	HAC vs. SC	80 vs. 23	1y
Jin [148]	2003	China	2	TACE+HIFU vs. TACE	24 vs. 26	1y
Katamura [149]	2009	Japan	2	HAC+RT vs. HAC	16 vs. 16	1y
Kawaoka [150]	2015	Japan	2	HAC vs. Sorafenib	16 vs. 16	1y
Kim [151]	2009	Korea	2	SR vs. SC	19 vs. 51	1y
Kim [152]	2014	Korea	3	TACE+RT vs. TACE vs. Sorafenib	66 vs.74 vs. 38	1y
Kodama [153]	2011	Japan	2	HAC+RT vs. HAC	19 vs. 26	1y
Lee [154]	2012	Taiwan	2	TACE vs. SC	27 vs. 27	1y
Lee [155]	1997	Korea	2	TACE vs. SC	31 vs. 16	1y
Li [156]	2011	China	2	TACE vs. TARE	30 vs. 26	1y
Li [157]	2016	China	2	TARE vs. TACE	38 vs. 36	1y
Li [158]	2016	China	2	SR+RT vs. SR	45 vs. 50	1y
Li [159]	2016	China	2	TARE+Sorafenib vs. TARE	15 vs. 38	1y
Li [160]	2016	China	2	TACE vs. SC	195 vs. 100	1y
Liang [161]	2008	China	2	SR+PVC vs. SR	33 vs. 53	1y
Lim [162]	2006	Korea	2	HAC vs. SC	40 vs. 39	1y
Liu [163]	2014	Taiwan	2	SR vs. TACE	108 vs. 108	1y
Liu [164]	2015	Taiwan	2	SR vs. TACE	163 vs. 163	1y
Lu [165]	2014	China	2	TACE+RT vs. TACE	30 vs. 33	1y
Luo [166]	2011	HK	2	TACE vs. SC	84 vs. 80	1y
Moreno-Luna [80]	2013	USA	2	TARE vs. TACE	14 vs. 19	1y

NAGAI [167]	2015	Japan	2	HAC+Sorafenib vs. HAC	18 vs. 20	1y
Nitta [168]	2013	Japan	2	SR+HAC vs. SR	38 vs. 35	1y
Niu [169]	2012	China	2	TACE vs. SC	115 vs. 35	1y
Onishi [170]	2015	Japan	2	HAC vs. HAC+RT	34 vs. 33	1y
Pinter [171]	2012	Austria	2	TACE vs. Sorafenib	34 vs. 63	1y
Radu [172]	2013	Romania	2	Sorafenib vs. SC	30 vs. 47	1y
Schmidt [173]	2014	Germany	2	TARE+Sorafenib vs. Sorafenib	16 vs. 21	1y
Song [174]	2014	Korea	2	Sorafenib vs. HAC	60 vs. 50	1y
TAN [175]	2014	China	2	TACE vs. TACE+PVE	64 vs. 52	1y
Tanaka [176]	1999	Japan	2	HAC+SR vs. SR	10 vs. 31	1y
Tang [177]	2013	China	2	TACE+SR vs. TACE+RT	186 vs. 185	1y
Torre [178]	2016	Spain	2	TARE vs. Sorafenib	26 vs. 46	1y
Vitale [93]	2015	Italy	2	SR vs. SC	42 vs. 184	1y
Wang [179]	2013	Taiwan	4	SR vs. Sorafenib vs. SC	68 vs. 11 vs. 140 vs. 88	1y
Wang [180]	2009	China	2	RT vs. TACE	10 vs. 12	1y
Wu [181]	2012	China	2	TARE vs. TACE	56 vs. 50	1y
Wu [182]	2011	China	2	SR vs. TACE+RT	64 vs. 81	1y
Xia [183]	2016	China	2	SR+Sorafenib vs. SR	34 vs. 68	1y
Xu [184]	2013	China	2	TACE+PEI vs. TACE	26 vs. 25	1y
Ye [185]	2014	China	4	SC vs. TACE vs. SR vs. SR+TACE	75 vs. 86 vs. 90 vs. 87	1y
Ye [186]	2016	China	5	TACE vs. TACE+Sorafenib vs. SR+TACE vs. Sorafenib vs. SC	274 vs. 33 vs. 54 vs. 15 vs. 42	1y
You [187]	2007	Korea	2	TACE+RT vs. TACE	27 vs. 22	1y
Yuan [188]	2016	China	2	SR vs. TACE	204 vs. 102	1y
ZHANG [189]	2015	China	2	TACE+Sorafenib vs. Sorafenib	45 vs.44	1y
Zhang [190]	2009	China	2	TACE+RT vs. TACE	15 vs. 28	1y
Zhang [192]	2016	China	2	TACE+Sorafenib vs. TACE	20 vs. 60	1y
Zhang [102]	2014	China	3	SR vs. SR+RFA vs. TACE	32 vs. 47 vs. 76	1y
Zhang [191]	2016	China	2	SR vs. SR+TACE	205 vs. 115	1y
Zhao [124]	2010	China	2	TACE vs. TACE+RFA	32 vs.21	1y
Zheng [193]	2016	China	2	SR vs. TACE	96 vs. 134	1y
Zhong [125]	2014	China	2	SR vs. TACE	248 vs. 85	1y
Zhou [194]	2011	China	3	TACE vs. SR vs. SR+PVC	10 vs. 38 vs. 31	1y
**BCLC Stage D**						
Andriulli [195]	2004	Italy	2	LT vs. PEI	61 vs. 21	1y
D'Avola [196]	2011	Spain	2	LT vs. SC	13 vs. 18	1y
Hiraoka [197]	2016	Japan	2	RFA vs. TACE	10 vs. 20	1y
Kudo [198]	2013	Japan	3	TACE vs. RFA vs. HAC	79 vs.60 vs. 64	1y

RELATED REFERENCES

[1] Dai Y, Li C, Wen TF, Yan LN. Comparison of liver resection and transplantation for Child-pugh A cirrhotic patient with very early hepatocellular carcinoma and portal hypertension. Pak J Med Sci 2014;30:996-1000.

[2] Farinati F, Sergio A, Baldan A, Giacomin A, Di Nolfo MA, Del PP, Benvegnu L, et al. Early and very early hepatocellular carcinoma: when and how much do staging and choice of treatment really matter? A multi-center study. BMC Cancer 2009;9:33.

[3] Harada N, Shirabe K, Maeda T, Kayashima H, Takaki S, Maehara Y. Comparison of the Outcomes of Patients with Hepatocellular Carcinoma and Portal Hypertension After Liver Resection Versus Radiofrequency Ablation. World J Surg 2016;40:1709-1719.

[4] Hasegawa K, Kokudo N, Makuuchi M, Izumi N, Ichida T, Kudo M, Ku Y, et al. Comparison of resection and ablation for hepatocellular carcinoma: a cohort study based on a Japanese nationwide survey. J Hepatol 2013;58:724-729.

[5] Horigome H, Nomura T, Saso K, Itoh M. Standards for selecting percutaneous ethanol injection therapy or percutaneous microwave coagulation therapy for solitary small hepatocellular carcinoma: consideration of local recurrence. Am J Gastroenterol 1999;94:1914-1917.

[6] Hung HH, Chiou YY, Hsia CY, Su CW, Chou YH, Chiang JH, Kao WY, et al. Survival rates are comparable after radiofrequency ablation or surgery in patients with small hepatocellular carcinomas. Clin Gastroenterol Hepatol 2011;9:79-86.

[7] Imai K, Beppu T, Chikamoto A, Doi K, Okabe H, Hayashi H, Nitta H, et al. Comparison between hepatic resection and radiofrequency ablation as first-line treatment for solitary small-sized hepatocellular carcinoma of 3 cm or less. Hepatol Res 2013;43:853-864.

[8] Kim GA, Shim JH, Kim MJ, Kim SY, Won HJ, Shin YM, Kim PN, et al. Radiofrequency ablation as an alternative to hepatic resection for single small hepatocellular carcinomas. Br J Surg 2016;103:126-135.

[9] Lin ZZ, Shau WY, Hsu C, Shao YY, Yeh YC, Kuo RN, Hsu CH, et al. Radiofrequency ablation is superior to ethanol injection in early-stage hepatocellular carcinoma irrespective of tumor size. PLoS One 2013;8:e80276.

[10] Liu PH, Hsu CY, Hsia CY, Lee YH, Huang YH, Chiou YY, Lin HC, et al. Surgical Resection Versus Radiofrequency Ablation for Single Hepatocellular Carcinoma </= 2 cm in a Propensity Score Model. Ann Surg 2016;263:538-545.

[11] Livraghi T, Meloni F, Di Stasi M, Rolle E, Solbiati L, Tinelli C, Rossi S. Sustained complete response and complications rates after radiofrequency ablation of very early hepatocellular carcinoma in cirrhosis: Is resection still the treatment of choice?. Hepatology 2008;47:82-89.

[12] Pompili M, Saviano A, de Matthaeis N, Cucchetti A, Ardito F, Federico B, Brunello F, et al. Long-term effectiveness of resection and radiofrequency ablation for single hepatocellular carcinoma </=3 cm. Results of a multicenter Italian survey. J Hepatol 2013;59:89-97.

[13] Pompili M, De Matthaeis N, Saviano A, De Sio I, Francica G, Brunello F, Cantamessa A, et al. Single hepatocellular carcinoma smaller than 2 cm: are ethanol injection and radiofrequency ablation equally effective?. Anticancer Res 2015;35:325-332.

[14] Santambrogio R, Bruno S, Kluger MD, Costa M, Salceda J, Belli A, Laurent A, et al. Laparoscopic ablation therapies or hepatic resection in cirrhotic patients with small hepatocellular carcinoma. Dig Liver Dis 2016;48:189-196.

[15] Takayama T, Makuuchi M, Hasegawa K. Single HCC smaller than 2 cm: surgery or ablation?: surgeon's perspective. J Hepatobiliary Pancreat Sci 2010;17:422-424.

[16] Takuma Y, Takabatake H, Morimoto Y, Toshikuni N, Kayahara T, Makino Y, Yamamoto H. Comparison of combined transcatheter arterial chemoembolization and radiofrequency ablation with surgical resection by using propensity score matching in patients with hepatocellular carcinoma within Milan criteria. Radiology 2013;269:927-937.

[17] Tashiro H, Aikata H, Waki K, Amano H, Oshita A, Kobayashi T, Tanimoto Y, et al. Treatment strategy for early hepatocellular carcinomas: comparison of radiofrequency ablation with or without transcatheter arterial chemoembolization and surgical resection. J Surg Oncol 2011;104:3-9.

[18] Wahl DR, Stenmark MH, Tao Y, Pollom EL, Caoili EM, Lawrence TS, Schipper MJ, et al. Outcomes After Stereotactic Body Radiotherapy or Radiofrequency Ablation for Hepatocellular Carcinoma. J Clin Oncol 2016;34:452-459.

[19] Wang JH, Wang CC, Hung CH, Chen CL, Lu SN. Survival comparison between surgical resection and radiofrequency ablation for patients in BCLC very early/early stage

hepatocellular carcinoma. J Hepatol 2012;56:412-418.

[20] Peng ZW, Lin XJ, Zhang YJ, Liang HH, Guo RP, Shi M, Chen MS. Radiofrequency ablation versus hepatic resection for the treatment of hepatocellular carcinomas 2 cm or smaller: a retrospective comparative study. Radiology 2012;262:1022-1033.

[21] Zhou Z, Lei J, Li B, Yan L, Wang W, Wei Y, Cheng K. Liver resection and radiofrequency ablation of very early hepatocellular carcinoma cases (single nodule <2 cm): a single-center study. Eur J Gastroenterol Hepatol 2014;26:339-344.

[22] Abu-Hilal M, Primrose JN, Casaril A, McPhail MJ, Pearce NW, Nicoli N. Surgical resection versus radiofrequency ablation in the treatment of small unifocal hepatocellular carcinoma. J Gastrointest Surg 2008;12:1521-1526.

[23] Baba Y, Hayashi S, Ueno K, Nakajo M, Ueno S, Kubo F, Baba Y, et al. Comparison of survival rates between patients treated with transcatheter arterial chemoembolization and hepatic resection for solitary hepatocellular carcinoma. Oncol Lett 2010;1:905-911.

[24] Bu X, Ge Z, Ma J, Guo S, Wang Y, Liu J. Long-term efficacy of radiofrequency ablation compared to surgical resection for the treatment of small hepatocellular carcinoma. J BUON 2015;20:548-554.

[25] Chen XH, Zhang BH, Qiu SJ, Fan J, Ren ZG, Xia JL, Wang YH, et al. [Effect of postoperative adjuvant transarterial chemoembolization on late recurrence of hepatocellular carcinoma after radical resection]. Zhonghua Gan Zang Bing Za Zhi 2010;18:599-603.

[26] Chen RX, Gan YH, Ge NL, Chen Y, Ma H, Wang Y, Zhang BH, et al. Comparison of transarterial chemoembolization with radiofrequency ablation for unresectable Barcelona Clinic Liver Cancer stage 0/A hepatocellular carcinoma: a propensity score matching. J Gastroenterol Hepatol 2016;31:442-449.

[27] Cheung TT, Fan ST, Chu FS, Jenkins CR, Chok KS, Tsang SH, Dai WC, et al. Survival analysis of high-intensity focused ultrasound ablation in patients with small hepatocellular carcinoma. HPB (Oxford) 2013;15:567-573.

[28] Cho YB, Lee KU, Suh KS, Kim YJ, Yoon JH, Lee HS, Hahn S, et al. Hepatic resection compared to percutaneous ethanol injection for small hepatocellular carcinoma using propensity score matching. J Gastroenterol Hepatol 2007;22:1643-1649.

[29] Daniele B, De Sio I, Izzo F, Capuano G, Andreana A, Mazzanti R, Aiello A, et al. Hepatic resection and percutaneous ethanol injection as treatments of small hepatocellular carcinoma: a Cancer of the Liver Italian Program (CLIP 08) retrospective case-control study. J Clin Gastroenterol 2003;36:63-67.

[30] Desiderio J, Trastulli S, Pasquale R, Cavaliere D, Cirocchi R, Boselli C, Noya G, et al. Could radiofrequency ablation replace liver resection for small hepatocellular carcinoma in patients with compensated cirrhosis? A 5-year follow-up. Langenbecks Arch Surg 2013;398:55-62.

[31] Ding J, Jing X, Liu J, Wang Y, Wang F, Wang Y, Du Z. Comparison of two different thermal techniques for the treatment of hepatocellular carcinoma. Eur J Radiol 2013;82:1379-1384.

[32] Gerunda GE, Neri D, Merenda R, Barbazza F, Zangrandi F, Meduri F, Bisello M, et al. Role of transarterial chemoembolization before liver resection for hepatocarcinoma. Liver Transpl 2000;6:619-626.

[33] Ginsburg M, Zivin SP, Wroblewski K, Doshi T, Vasnani RJ, Van Ha TG. Comparison of combination therapies in the management of hepatocellular carcinoma: transarterial chemoembolization with radiofrequency ablation versus microwave ablation. J Vasc Interv Radiol 2015;26:330-341.

[34] Gory I, Fink M, Bell S, Gow P, Nicoll A, Knight V, Dev A, et al. Radiofrequency ablation versus resection for the treatment of early stage hepatocellular carcinoma: a multicenter Australian study. Scand J Gastroenterol 2015;50:567-576.

[35] Gournay J, Tchuenbou J, Richou C, Masliah C, Lerat F, Dupas B, Martin T, et al. Percutaneous ethanol injection vs. resection in patients with small single hepatocellular carcinoma: a retrospective case-control study with cost analysis. Aliment Pharmacol Ther 2002;16:1529-1538.

[36] Graham JA, Newman DA, Smirniotopolous J, Shetty K, Slidell MB, Johnson LB. Transplantation for hepatocellular carcinoma in younger patients has an equivocal survival advantage as compared with resection. Transplant Proc 2013;45:265-271.

[37] Graham JA, Melancon JK, Shetty K, Johnson LB. Liver transplantation should be offered to patients with small solitary hepatocellular carcinoma and a positive serum alpha fetoprotein rather than resection. Am J Surg 2013;205:374-380.

[38] Guan TP, Fang CH, Yang J, Xiang N, Chen QS, Zhong SZ. A Comparison between Three-Dimensional Visualization Guided Hepatectomy and Ultrasonography Guided Radiofrequency Ablation in the Treatment of Small Hepatocellular Carcinoma within the Milan Criteria. Biomed Res Int 2016;2016:8931732.

[39] Guo WX, Zhai B, Lai EC, Li N, Shi J, Lau WY, Wu MC, et al. Percutaneous radiofrequency ablation versus partial hepatectomy for multicentric small hepatocellular carcinomas: a nonrandomized comparative study. World J Surg 2010;34:2671-2676.

[40] Guo Z, Zhong JH, Jiang JH, Zhang J, Xiang BD, Li LQ. Comparison of survival of patients with BCLC stage A hepatocellular carcinoma after hepatic resection or transarterial chemoembolization: a propensity score-based analysis. Ann Surg Oncol 2014;21:3069-3076.

[41] Guo Z, Xiang B, Zhang J, Jiang J, Li L. [Comparison of hepatic resection and transarterial chemoembolization for hepatocellular carcinoma within the Milan criteria]. Zhonghua Yi Xue Za Zhi 2014;94:1526-1529.

[42] Harada N, Maeda T, Yoshizumi T, Ikeda T, Kayashima H, Ikegami T, Harimoto N, et al. Laparoscopic Liver Resection Is a Feasible Treatment for Patients with Hepatocellular Carcinoma and Portal Hypertension. Anticancer Res 2016;36:3489-3497.

[43] Hasegawa K, Makuuchi M, Takayama T, Kokudo N, Arii S, Okazaki M, Okita K, et al. Surgical resection vs. percutaneous ablation for hepatocellular carcinoma: a preliminary report of the Japanese nationwide survey. J Hepatol 2008;49:589-594.

[44] Schoniger-Hekele M, Kettenbach J, Peck-Radosavljevic M, Muller C. Octreotide treatment of patients with hepatocellular carcinoma--a retrospective single centre controlled study. J Exp Clin Cancer Res 2009;28:142.

[45] Hiraoka A, Horiike N, Yamashita Y, Koizumi Y, Doi K, Yamamoto Y, Hasebe A, et al. Efficacy of radiofrequency ablation therapy compared to surgical resection in 164 patients in Japan with single hepatocellular carcinoma smaller than 3 cm, along with report of complications. Hepatogastroenterology 2008;55:2171-2174.

[46] Ho MC, Huang GT, Tsang YM, Lee PH, Chen DS, Sheu JC, Chen CH. Liver resection improves the survival of patients with multiple hepatocellular carcinomas. Ann Surg Oncol 2009;16:848-855.

[47] Hocquelet A, Balageas P, Laurent C, Blanc JF, Frulio N, Salut C, Cassinotto C, et al. Radiofrequency ablation versus surgical resection for hepatocellular carcinoma within the Milan criteria: A study of 281 Western patients. Int J Hyperthermia 2015;31:749-757.

[48] Honda Y, Kimura T, Aikata H, Kobayashi T, Fukuhara T, Masaki K, Nakahara T, et al. Stereotactic body radiation therapy combined with transcatheter arterial chemoembolization for small hepatocellular carcinoma. J Gastroenterol Hepatol 2013;28:530-536.

[49] Hong SN, Lee SY, Choi MS, Lee JH, Koh KC, Paik SW, Yoo BC, et al. Comparing the outcomes of radiofrequency ablation and surgery in patients with a single small hepatocellular carcinoma and well-preserved hepatic function. J Clin Gastroenterol 2005;39:247-252.

[50] Hsu KF, Chu CH, Chan DC, Yu JC, Shih ML, Hsieh HF, Hsieh TY, et al. Superselective transarterial chemoembolization vs hepatic resection for resectable early-stage hepatocellular carcinoma in patients with Child-Pugh class a liver function. Eur J Radiol 2012;81:466-471.

[51] Huang J, Hernandez-Alejandro R, Croome KP, Yan L, Wu H, Chen Z, Prasoon P, et al. Radiofrequency ablation versus surgical resection for hepatocellular carcinoma in Childs A cirrhotics-a retrospective study of 1,061 cases. J Gastrointest Surg 2011;15:311-320.

[52] Huang G, Chen X, Lau WY, Shen F, Wang RY, Yuan SX, Geng WX, et al. Quality of life after surgical resection compared with radiofrequency ablation for small hepatocellular carcinomas. Br J Surg 2014;101:1006-1015.

[53] Huo TI, Huang YH, Wu JC, Chiang JH, Lee PC, Chang FY, Lee SD. Sequential transarterial chemoembolization and percutaneous acetic acid injection therapy versus repeated percutaneous acetic acid injection for unresectable hepatocellular carcinoma: a prospective study. Ann Oncol 2003;14:1648-1653.

[54] Huo T, Huang YH, Wu JC, Chiang JH, Lee PC, Chang FY, Lee SD. Comparison of transarterial chemoembolization and percutaneous acetic acid injection as the primary loco-regional therapy for unresectable hepatocellular carcinoma: a prospective survey. Aliment Pharmacol Ther 2004;19:1301-1308.

[55] Hyun D, Cho SK, Shin SW, Park KB, Park HS, Choo SW, Do YS, et al. Early Stage Hepatocellular Carcinomas Not Feasible for Ultrasound-Guided Radiofrequency Ablation: Comparison of Transarterial Chemoembolization Alone and Combined Therapy with Transarterial Chemoembolization and Radiofrequency Ablation. Cardiovasc Intervent Radiol 2016;39:417-425.

[56] Jiang L, Yan L, Wen T, Li B, Zeng Y, Yang J, Wang W, et al. Comparison of Outcomes of Hepatic Resection and Radiofrequency Ablation for Hepatocellular Carcinoma Patients with Multifocal Tumors Meeting the Barcelona-Clinic Liver Cancer Stage A Classification. J Am Coll Surg 2015;221:951-961.

[57] Kagawa T, Koizumi J, Kojima S, Nagata N, Numata M, Watanabe N, Watanabe T, et al. Transcatheter arterial chemoembolization plus radiofrequency ablation therapy for early stage hepatocellular carcinoma: comparison with surgical resection. Cancer 2010;116:3638-3644.

[58] Kamada K, Kitamoto M, Aikata H, Kawakami Y, Kono H, Imamura M, Nakanishi T, et al. Combination of transcatheter arterial chemoembolization using cisplatin-lipiodol suspension and percutaneous ethanol injection for treatment of advanced small hepatocellular carcinoma. Am J Surg 2002;184:284-290.

[59] Kang TW, Kim JM, Rhim H, Lee MW, Kim YS, Lim HK, Choi D, et al. Small Hepatocellular Carcinoma: Radiofrequency Ablation versus Nonanatomic Resection--Propensity Score

Analyses of Long-term Outcomes. Radiology 2015;275:908-919.

[60] Kim JM, Kang TW, Kwon CH, Joh JW, Ko JS, Park JB, Rhim H, et al. Single hepatocellular carcinoma </= 3 cm in left lateral segment: liver resection or radiofrequency ablation?. World J Gastroenterol 2014;20:4059-4065.

[61] Kim DY, Choi MS, Lee JH, Koh KC, Paik SW, Yoo BC, Shin SW, et al. Milan criteria are useful predictors for favorable outcomes in hepatocellular carcinoma patients undergoing liver transplantation after transarterial chemoembolization. World J Gastroenterol 2006;12:6992-6997.

[62] Kim JW, Kim JH, Won HJ, Shin YM, Yoon HK, Sung KB, Kim PN. Hepatocellular carcinomas 2-3 cm in diameter: transarterial chemoembolization plus radiofrequency ablation vs. radiofrequency ablation alone. Eur J Radiol 2012;81:e189-e193.

[63] Kim J, Chung DJ, Jung SE, Cho SH, Hahn ST, Lee JM. Therapeutic effect of high-intensity focused ultrasound combined with transarterial chemoembolisation for hepatocellular carcinoma <5 cm: comparison with transarterial chemoembolisation monotherapy--preliminary observations. Br J Radiol 2012;85:e940-e946.

[64] Kim JW, Shin SS, Kim JK, Choi SK, Heo SH, Lim HS, Hur YH, et al. Radiofrequency ablation combined with transcatheter arterial chemoembolization for the treatment of single hepatocellular carcinoma of 2 to 5 cm in diameter: comparison with surgical resection. Korean J Radiol 2013;14:626-635.

[65] Kobayashi M, Ikeda K, Kawamura Y, Yatsuji H, Hosaka T, Sezaki H, Akuta N, et al. High serum des-gamma-carboxy prothrombin level predicts poor prognosis after radiofrequency ablation of hepatocellular carcinoma. Cancer 2009;115:571-580.

[66] Koniaris LG, Levi DM, Pedroso FE, Franceschi D, Tzakis AG, Santamaria-Barria JA, Tang J, et al. Is surgical resection superior to transplantation in the treatment of hepatocellular carcinoma?. Ann Surg 2011;254:527-537, 537-538.

[67] Lai EC, Tang CN. Radiofrequency ablation versus hepatic resection for hepatocellular carcinoma within the Milan criteria--a comparative study. Int J Surg 2013;11:77-80.

[68] Lai C, Jin RA, Liang X, Cai XJ. Comparison of laparoscopic hepatectomy, percutaneous radiofrequency ablation and open hepatectomy in the treatment of small hepatocellular carcinoma. J Zhejiang Univ Sci B 2016;17:236-246.

[69] Lee KF, Wong J, Hui JW, Cheung YS, Chong CC, Fong AK, Yu SC, et al. Long-term outcomes of microwave versus radiofrequency ablation for hepatocellular carcinoma by surgical approach: A retrospective comparative study. Asian J Surg 2016.

[70] Lee YH, Hsu CY, Chu CW, Liu PH, Hsia CY, Huang YH, Su CW, et al. Radiofrequency ablation is better than surgical resection in patients with hepatocellular carcinoma within the Milan criteria and preserved liver function: a retrospective study using propensity score analyses. J Clin Gastroenterol 2015;49:242-249.

[71] Lei JY, Wang WT, Yan LN, Wen TF, Li B. Radiofrequency ablation versus surgical resection for small unifocal hepatocellular carcinomas. Medicine (Baltimore) 2014;93:e271.

[72] Li W, Zhou X, Huang Z, Zhang K, Luo X, Zhong J, Chen Y. Short- and Long-term Outcomes of Laparoscopic Hepatectomy, Microwave Ablation and Open Hepatectomy for Small Hepatocellular Carcinoma: A 5-Year Experience in a Single Center. Hepatol Res 2016.

[73] Li Z, Zhang C, Lou C, Yan F, Mao Y, Hong X, Zhang Y. Comparison of percutaneous cryosurgery and surgical resection for the treatment of small hepatocellular carcinoma. Oncol Lett 2013;6:239-245.

[74] Li C, Zhu WJ, Wen TF, Dai Y, Yan LN, Li B, Yang JY, et al. Child-Pugh A hepatitis B-related cirrhotic patients with a single hepatocellular carcinoma up to 5 cm: liver transplantation vs. resection. J Gastrointest Surg 2014;18:1469-1476.

[75] Iida H, Aihara T, Ikuta S, Yamanaka N. Comparative study of percutaneous radiofrequency ablation and hepatic resection for small, poorly differentiated hepatocellular carcinomas. Hepatol Res 2014;44:E156-E162.

[76] Liu PH, Lee YH, Hsu CY, Huang YH, Chiou YY, Lin HC, Huo TI. Survival advantage of radiofrequency ablation over transarterial chemoembolization for patients with hepatocellular carcinoma and good performance status within the Milan criteria. Ann Surg Oncol 2014;21:3835-3843.

[77] Lupo L, Panzera P, Giannelli G, Memeo M, Gentile A, Memeo V. Single hepatocellular carcinoma ranging from 3 to 5 cm: radiofrequency ablation or resection?. HPB (Oxford) 2007;9:429-434.

[78] Montorsi M, Santambrogio R, Bianchi P, Donadon M, Moroni E, Spinelli A, Costa M. Survival and recurrences after hepatic resection or radiofrequency for hepatocellular carcinoma in cirrhotic patients: a multivariate analysis. J Gastrointest Surg 2005;9:62-67, 67-68.

[79] Moon DB, Lee SG, Hwang S. Liver transplantation for hepatocellular carcinoma: single nodule with Child-Pugh class A sized less than 3 cm. Dig Dis 2007;25:320-328.

[80] Moreno-Luna LE, Yang JD, Sanchez W, Paz-Fumagalli R, Harnois DM, Mettler TA, Gansen DN, et al. Efficacy and safety of transarterial radioembolization versus chemoembolization in patients with hepatocellular carcinoma. Cardiovasc Intervent Radiol 2013;36:714-723.

[81] Park EK, Kim HJ, Kim CY, Hur YH, Koh YS, Kim JC, Kim HJ, et al. A comparison between surgical resection and radiofrequency ablation in the treatment of hepatocellular carcinoma. Ann Surg Treat Res 2014;87:72-80.

[82] Peng ZW, Liu FR, Ye S, Xu L, Zhang YJ, Liang HH, Lin XJ, et al. Radiofrequency ablation versus open hepatic resection for elderly patients (> 65 years) with very early or early hepatocellular carcinoma. Cancer 2013;119:3812-3820.

[83] Potretzke TA, Ziemlewicz TJ, Hinshaw JL, Lubner MG, Wells SA, Brace CL, Agarwal P, et al. Microwave versus Radiofrequency Ablation Treatment for Hepatocellular Carcinoma: A Comparison of Efficacy at a Single Center. J Vasc Interv Radiol 2016;27:631-638.

[84] Santambrogio R, Opocher E, Zuin M, Selmi C, Bertolini E, Costa M, Conti M, et al. Surgical resection versus laparoscopic radiofrequency ablation in patients with hepatocellular carcinoma and Child-Pugh class a liver cirrhosis. Ann Surg Oncol 2009;16:3289-3298.

[85] Shi J, Sun Q, Wang Y, Jing X, Ding J, Yuan Q, Ren C, et al. Comparison of microwave ablation and surgical resection for treatment of hepatocellular carcinomas conforming to Milan criteria. J Gastroenterol Hepatol 2014;29:1500-1507.

[86] Shi F, Tan Z, An H, Wang X, Xu Y, Wang S. Hepatocellular carcinoma </= 4 cm treated with radiofrequency ablation with or without percutaneous ethanol injection. Ann Hepatol 2016;15:61-70.

[87] Song MJ, Bae SH, Lee JS, Lee SW, Song DS, You CR, Choi JY, et al. Combination transarterial chemoembolization and radiofrequency ablation therapy for early hepatocellular carcinoma. Korean J Intern Med 2016;31:242-252.

[88] Song J, Wang Y, Ma K, Zheng S, Bie P, Xia F, Li X, et al. Laparoscopic hepatectomy versus radiofrequency ablation for minimally invasive treatment of single, small hepatocellular carcinomas. Surg Endosc 2016;30:4249-4257.

[89] Sotiropoulos GC, Druhe N, Sgourakis G, Molmenti EP, Beckebaum S, Baba HA, Antoch G, et al. Liver transplantation, liver resection, and transarterial chemoembolization for hepatocellular carcinoma in cirrhosis: which is the best oncological approach?. Dig Dis Sci 2009;54:2264-2273.

[90] Takahashi S, Kudo M, Chung H, Inoue T, Nagashima M, Kitai S, Tatsumi C, et al. Outcomes of nontransplant potentially curative therapy for early-stage hepatocellular carcinoma in Child-Pugh stage A cirrhosis is comparable with liver transplantation. Dig Dis 2007;25:303-309.

[91] Tohme S, Geller DA, Cardinal JS, Chen HW, Packiam V, Reddy S, Steel J, et al. Radiofrequency ablation compared to resection in early-stage hepatocellular carcinoma. HPB (Oxford) 2013;15:210-217.

[92] Ueno S, Sakoda M, Kubo F, Hiwatashi K, Tateno T, Baba Y, Hasegawa S, et al. Surgical resection versus radiofrequency ablation for small hepatocellular carcinomas within the Milan criteria. J Hepatobiliary Pancreat Surg 2009;16:359-366.

[93] Vitale A, Burra P, Frigo AC, Trevisani F, Farinati F, Spolverato G, Volk M, et al. Survival benefit of liver resection for patients with hepatocellular carcinoma across different Barcelona Clinic Liver Cancer stages: a multicentre study. J Hepatol 2015;62:617-624.

[94] Vitali GC, Laurent A, Terraz S, Majno P, Buchs NC, Rubbia-Brandt L, Luciani A, et al. Minimally invasive surgery versus percutaneous radio frequency ablation for the treatment of single small (</=3 cm) hepatocellular carcinoma: a case-control study. Surg Endosc 2016;30:2301-2307.

[95] Vivarelli M, Guglielmi A, Ruzzenente A, Cucchetti A, Bellusci R, Cordiano C, Cavallari A. Surgical resection versus percutaneous radiofrequency ablation in the treatment of hepatocellular carcinoma on cirrhotic liver. Ann Surg 2004;240:102-107.

[96] Wong KM, Yeh ML, Chuang SC, Wang LY, Lin ZY, Chen SC, Tsai JF, et al. Survival comparison between surgical resection and percutaneous radiofrequency ablation for patients in Barcelona Clinic Liver Cancer early stage hepatocellular carcinoma. Indian J Gastroenterol 2013;32:253-257.

[97] Yamakado K, Nakatsuka A, Takaki H, Yokoi H, Usui M, Sakurai H, Isaji S, et al. Early-stage hepatocellular carcinoma: radiofrequency ablation combined with chemoembolization versus hepatectomy. Radiology 2008;247:260-266

[98] Yan SY, Zhang Y, Sun C, Cao HX, Li GM, Wang YQ, Fan JG. The clinical effect and relevant mechanism of combined sorafenib and radiofrequency ablation in the treatment of early small hepatocellular carcinoma. Oncol Lett 2016;12:951-955.

[99] Yang HJ, Lee JH, Lee DH, Yu SJ, Kim YJ, Yoon JH, Kim HC, et al. Small single-nodule hepatocellular carcinoma: comparison of transarterial chemoembolization, radiofrequency ablation, and hepatic resection by using inverse probability weighting. Radiology 2014;271:909-918.

[100] Yun WK, Choi MS, Choi D, Rhim HC, Joh JW, Kim KH, Jang TH, et al. Superior long-term outcomes after surgery in child-pugh class a patients with single small hepatocellular carcinoma compared to radiofrequency ablation. Hepatol Int 2011;5:722-729.

[101] Zhang K, Jiang L, Jia Z, Zhang Y, He R, Ding Z, Mu Y. Radiofrequency ablation plus devascularization is the preferred treatment of hepatocellular carcinoma with esophageal varices. Dig Dis Sci 2015;60:1490-1501.

[102] Zhang T, Zeng Y, Huang J, Liao M, Wu H. Combined resection with radiofrequency ablation for bilobar hepatocellular carcinoma: a single-center experience. J Surg Res 2014;191:370-378.

[103] Zhang EL, Yang F, Wu ZB, Yue CS, He TY, Li KY, Xiao ZY, et al. Therapeutic efficacy of percutaneous microwave coagulation versus liver resection for single hepatocellular carcinoma </=3 cm with Child-Pugh A cirrhosis. Eur J Surg Oncol 2016;42:690-697.

[104] Arizumi T, Ueshima K, Minami T, Kono M, Chishina H, Takita M, Kitai S, et al. Effectiveness of Sorafenib in Patients with Transcatheter Arterial Chemoembolization (TACE) Refractory and Intermediate-Stage Hepatocellular Carcinoma. Liver Cancer 2015;4:253-262.

[105] Azuma S, Asahina Y, Nishimura-Sakurai Y, Kakinuma S, Kaneko S, Nagata H, Goto F, et al. Efficacy of additional radiofrequency ablation after transcatheter arterial chemoembolization for intermediate hepatocellular carcinoma. Hepatol Res 2016;46:312-319

[106] Ciria R, Lopez-Cillero P, Gallardo AB, Cabrera J, Pleguezuelo M, Ayllon MD, Luque A, et al. Optimizing the management of patients with BCLC stage-B hepatocellular carcinoma: Modern surgical resection as a feasible alternative to transarterial chemoemolization. Eur J Surg Oncol 2015;41:1153-1161.

[107] El FA, Ertle J, El DA, Shaker MK, Dechene A, Abdella H, Mueller S, et al. In intermediate stage hepatocellular carcinoma: radioembolization with yttrium 90 or chemoembolization?. Liver Int 2015;35:627-635.

[108] Li F, Guo Z, Zhang Y, Wang H, Zhang X, Si T, Yu H, et al. Postoperative adjuvant arterial chemoembolization improves the survival of hepatitis B virus-related hepatocellular carcinoma: a retrospective control study. Ir J Med Sci 2015;184:753-759

[109] Ho EY, Cozen ML, Shen H, Lerrigo R, Trimble E, Ryan JC, Corvera CU, et al. Expanded use of aggressive therapies improves survival in early and intermediate hepatocellular carcinoma. HPB (Oxford) 2014;16:758-767.

[110] Jacob R, Turley F, Redden DT, Saddekni S, Aal AK, Keene K, Yang E, et al. Adjuvant stereotactic body radiotherapy following transarterial chemoembolization in patients with non-resectable hepatocellular carcinoma tumours of >/= 3 cm. HPB (Oxford) 2015;17:140-149.

[111] Jin YJ, Lee JW, Choi YJ, Chung HJ, Kim YS, Lee KY, Ahn SI, et al. Surgery versus transarterial chemoembolization for solitary large hepatocellular carcinoma of BCLC stage A. J Gastrointest Surg 2014;18:555-561.

[112] Ke Y, Zhong J, Guo Z, Liang Y, Li L, Xiang B. [Comparison liver resection with transarterial chemoembolization for Barcelona Clinic Liver Cancer stage B hepatocellular carcinoma patients on long-term survival after SPSS propensity score matching]. Zhonghua Yi Xue Za Zhi 2014;94:747-750.

[113] Kim JY, Sinn DH, Gwak GY, Choi GS, Saleh AM, Joh JW, Cho SK, et al. Transarterial chemoembolization versus resection for intermediate-stage (BCLC B) hepatocellular carcinoma. Clin Mol Hepatol 2016;22:250-258.

[114] Lee YB, Lee DH, Cho Y, Yu SJ, Lee JH, Yoon JH, Lee HS, et al. Comparison of transarterial chemoembolization and hepatic resection for large solitary hepatocellular carcinoma: a propensity score analysis. J Vasc Interv Radiol 2015;26:651-659.

[115] Jianyong L, Lunan Y, Wentao W, Yong Z, Bo L, Tianfu W, Minqing X, et al. Barcelona clinic liver cancer stage B hepatocellular carcinoma: transarterial chemoembolization or hepatic resection?. Medicine (Baltimore) 2014;93:e180.

[116] Lin CT, Hsu KF, Chen TW, Yu JC, Chan DC, Yu CY, Hsieh TY, et al. Comparing hepatic resection and transarterial chemoembolization for Barcelona Clinic Liver Cancer (BCLC) stage B hepatocellular carcinoma: change for treatment of choice?. World J Surg 2010;34:2155-2161

[117] Liu PH, Su CW, Hsu CY, Hsia CY, Lee YH, Huang YH, Lee RC, et al. Solitary Large Hepatocellular Carcinoma: Staging and Treatment Strategy. PLoS One 2016;11:e155588.

[118] Lubienski A, Bitsch RG, Schemmer P, Grenacher L, Dux M, Kauffmann GW. [Long-term results of interventional treatment of large unresectable hepatocellular carcinoma (HCC): significant survival benefit from combined transcatheter arterial chemoembolization (TACE) and percutaneous ethanol injection (PEI) compared to TACE monotherapy]. Rofo 2004;176:1794-1802.

[119] Luo J, Peng ZW, Guo RP, Zhang YQ, Li JQ, Chen MS, Shi M. Hepatic resection versus transarterial lipiodol chemoembolization as the initial treatment for large, multiple, and resectable hepatocellular carcinomas: a prospective nonrandomized analysis. Radiology 2011;259:286-295.

[120] Nishikawa H, Osaki Y, Kita R, Kimura T, Ohara Y, Takeda H, Sakamoto A, et al. Comparison of transcatheter arterial chemoembolization and transcatheter arterial chemotherapy infusion for patients with intermediate-stage hepatocellular carcinoma. Oncol Rep 2014;31:65-72.

[121] Pecorelli A, Lenzi B, Gramenzi A, Garuti F, Farinati F, Giannini EG, Ciccarese F, et al. Curative therapies are superior to standard of care (transarterial chemoembolization) for intermediate stage hepatocellular carcinoma. Liver Int 2016.

[122] Shim SJ, Seong J, Han KH, Chon CY, Suh CO, Lee JT. Local radiotherapy as a complement to incomplete transcatheter arterial chemoembolization in locally advanced hepatocellular carcinoma. Liver Int 2005;25:1189-1196.

[123] Yin X, Zhang L, Wang YH, Zhang BH, Gan YH, Ge NL, Chen Y, et al. Transcatheter arterial chemoembolization combined with radiofrequency ablation delays tumor progression and prolongs overall survival in patients with intermediate (BCLC B) hepatocellular carcinoma. BMC Cancer 2014;14:849.

[124] Zhao M, Wang JP, Wu PH, Zhang FJ, Huang ZL, Li W, Zhang L, et al. [Comparative analysis of TACE alone or plus RFA in the treatment of 167 cases of intermediate and advanced staged primary hepatocellular carcinoma]. Zhonghua Yi Xue Za Zhi 2010;90:2916-2921.

[125] Zhong JH, Ke Y, Gong WF, Xiang BD, Ma L, Ye XP, Peng T, et al. Hepatic resection associated with good survival for selected patients with intermediate and advanced-stage hepatocellular carcinoma. Ann Surg 2014;260:329-340.

[126] Zhong JH, Xiang BD, Gong WF, Ke Y, Mo QG, Ma L, Liu X, et al. Comparison of long-term survival of patients with BCLC stage B hepatocellular carcinoma after liver resection or transarterial chemoembolization. PLoS One 2013;8:e68193.

[127] Bai T, Chen J, Xie ZB, Wu FX, Wang SD, Liu JJ, Li LQ. The efficacy and safety of postoperative adjuvant transarterial embolization and radiotherapy in hepatocellular carcinoma patients with portal vein tumor thrombus. Onco Targets Ther 2016;9:3841-3848.

[128] Chen LW, Chien RN, Fang KM, Yen CL, Chang JJ, Lee TS, Liu CJ. Elucidating therapeutic effects on patients with hepatocellular carcinoma and main portal vein thrombosis. Hepatogastroenterology 2010;57:228-231.

[129] Chen J, Xi W, Wu B, Yu H, Chen S. [Clinical observation of transcatheter arterial chemoembolization plus sorafenib in the treatment of hepatocellular carcinoma with portal vein tumor thrombosis]. Zhonghua Yi Xue Za Zhi 2014;94:2566-2569.

[130] Cheng SQ, Wu MC, Chen H, Shen F, Yang JH, Cong WM, Zhao YX, et al. [Hepatocellular carcinoma with tumor thrombi in the portal vein. A comparison of therapeutic effects by different treatments]. Zhonghua Zhong Liu Za Zhi 2005;27:183-185.

[131] Cheong JY, Lee KM, Cho SW, Won JH, Kim JK, Wang HJ, Hahm KB, et al. Survival benefits of intra-arterial infusion chemotherapy in patients with advanced hepatocellular carcinoma with portal vein tumor thrombosis. Hepatol Res 2005;32:127-133.

[132] Cheung TT, Poon RT, Jenkins CR, Chu FS, Chok KS, Chan AC, Tsang SH, et al. Survival analysis of high-intensity focused ultrasound therapy vs. transarterial chemoembolization for unresectable hepatocellular carcinomas. Liver Int 2014;34:e136-e143.

[133] Cho JY, Paik YH, Park HC, Yu JI, Sohn W, Gwak GY, Choi MS, et al. The feasibility of combined transcatheter arterial chemoembolization and radiotherapy for advanced hepatocellular carcinoma. Liver Int 2014;34:795-801.

[134] Cho YY, Lee M, Kim HC, Chung JW, Kim YH, Gwak GY, Bae SH, et al. Radioembolization Is a Safe and Effective Treatment for Hepatocellular Carcinoma with Portal Vein Thrombosis: A Propensity Score Analysis. PLoS One 2016;11:e154986.

[135] Choi JW, Kim HC, Lee JH, Yu SJ, Kim YJ, Yoon JH, Jae HJ, et al. Transarterial chemoembolization of hepatocellular carcinoma with segmental portal vein tumour thrombus. Eur Radiol 2016.

[136] Choi GH, Shim JH, Kim MJ, Ryu MH, Ryoo BY, Kang YK, Shin YM, et al. Sorafenib alone versus sorafenib combined with transarterial chemoembolization for advanced-stage hepatocellular carcinoma: results of propensity score analyses. Radiology 2013;269:603-611.

[137] Chung GE, Lee JH, Kim HY, Hwang SY, Kim JS, Chung JW, Yoon JH, et al. Transarterial chemoembolization can be safely performed in patients with hepatocellular carcinoma invading the main portal vein and may improve the overall survival. Radiology 2011;258:627-634.

[138] Edeline J, Crouzet L, Campillo-Gimenez B, Rolland Y, Pracht M, Guillygomarc'h A, Boudjema K, et al. Selective internal radiation therapy compared with sorafenib for hepatocellular carcinoma with portal vein thrombosis. Eur J Nucl Med Mol Imaging 2016;43:635-643.

[139] Fan J, Zhou J, Wu ZQ, Qiu SJ, Wang XY, Shi YH, Tang ZY. Efficacy of different treatment strategies for hepatocellular carcinoma with portal vein tumor thrombosis. World J Gastroenterol 2005;11:1215-1219.

[140] Fan J, Wu ZQ, Tang ZY, Zhou J, Qiu SJ, Ma ZC, Zhou XD, et al. Multimodality treatment in hepatocellular carcinoma patients with tumor thrombi in portal vein. World J Gastroenterol 2001;7:28-32.

[141] Fan J, Wu ZQ, Zhou J, Qiu SJ, Shi YH, Chen RX, Tang ZY. Hepatocellular carcinoma associated with tumor thrombosis in the portal vein: the effects of different treatments. Hepatobiliary Pancreat Dis Int 2003;2:513-519.

[142] Ha Y, Lee D, Shim JH, Lim YS, Lee HC, Chung YH, Lee YS, et al. Role of transarterial chemoembolization in relation with sorafenib for patients with advanced hepatocellular carcinoma. Oncotarget 2016.

[143] Hirooka M, Koizumi Y, Kisaka Y, Abe M, Murakami H, Matsuura B, Hiasa Y, et al. Mass reduction by radiofrequency ablation before hepatic arterial infusion chemotherapy improved prognosis for patients with huge hepatocellular carcinoma and portal vein thrombus. AJR Am J Roentgenol 2010;194:W221-W226.

[144] Hu H, Duan Z, Long X, Hertzanu Y, Shi H, Liu S, Yang Z. Sorafenib combined with transarterial chemoembolization versus transarterial chemoembolization alone for advanced-stage hepatocellular carcinoma: a propensity score matching study. PLoS One 2014;9:e96620.

[145] Hu WJ, Liang LJ, Zhou Q, Peng BG, Yin XY, Li DM. [Efficacy of postoperative chemotherapy combined with immunotherapy for hepatocellular carcinoma with major portal vein tumor thrombus]. Zhonghua Wai Ke Za Zhi 2007;45:1325-1327.

[146] Huang M, Lin Q, Wang H, Chen J, Bai M, Wang L, Zhu K, et al. Survival benefit of chemoembolization plus Iodine125 seed implantation in unresectable hepatitis B-related hepatocellular carcinoma with PVTT: a retrospective matched cohort study. Eur Radiol 2016.

[147] Jang JW, Bae SH, Choi JY, Oh HJ, Kim MS, Lee SY, Kim CW, et al. A combination therapy with transarterial chemo-lipiodolization and systemic chemo-infusion for large extensive hepatocellular carcinoma invading portal vein in comparison with conservative management. Cancer Chemother Pharmacol 2007;59:9-15.

[148] Jin CB, Wu F, Wang ZB, Chen WZ, Zhu H. [High intensity focused ultrasound therapy combined with transcatheter arterial chemoembolization for advanced hepatocellular carcinoma]. Zhonghua Zhong Liu Za Zhi 2003;25:401-403.

[149] Katamura Y, Aikata H, Takaki S, Azakami T, Kawaoka T, Waki K, Hiramatsu A, et al. Intra-arterial 5-fluorouracil/interferon combination therapy for advanced hepatocellular carcinoma with or without three-dimensional conformal radiotherapy for portal vein tumor thrombosis. J Gastroenterol 2009;44:492-502.

[150] Kawaoka T, Aikata H, Hyogo H, Morio R, Morio K, Hatooka M, Fukuhara T, et al. Comparison of hepatic arterial infusion chemotherapy versus sorafenib monotherapy in patients with advanced hepatocellular carcinoma. J Dig Dis 2015;16:505-512.

[151] Kim KM, Kim JH, Park IS, Ko GY, Yoon HK, Sung KB, Lim YS, et al. Reappraisal of repeated transarterial chemoembolization in the treatment of hepatocellular carcinoma with portal vein invasion. J Gastroenterol Hepatol 2009;24:806-814.

[152] Kim GA, Shim JH, Yoon SM, Jung J, Kim JH, Ryu MH, Ryoo BY, et al. Comparison of chemoembolization with and without radiation therapy and sorafenib for advanced hepatocellular carcinoma with portal vein tumor thrombosis: a propensity score analysis. J Vasc Interv Radiol 2015;26:320-329.

[153] Kodama H, Aikata H, Murakami E, Miyaki D, Nagaoki Y, Hashimoto Y, Azakami T, et al. Clinical outcome of esophageal varices after hepatic arterial infusion chemotherapy for advanced hepatocellular carcinoma with major portal vein tumor thrombus. Hepatol Res 2011;41:1046-1056.

[154] Lee IC, Huo TI, Huang YH, Chao Y, Li CP, Lee PC, Chiang JH, et al. Transarterial chemoembolization can prolong survival for patients with metastatic hepatocellular carcinoma: a propensity score matching analysis. Hepatol Int 2012;6:753-762.

[155] Lee HS, Kim JS, Choi IJ, Chung JW, Park JH, Kim CY. The safety and efficacy of transcatheter arterial chemoembolization in the treatment of patients with hepatocellular carcinoma and main portal vein obstruction. A prospective controlled study. Cancer 1997;79:2087-2094.

[156] Chuan-Xing L, Xu H, Bao-Shan H, Yong L, Pei-Jian S, Xian-Yi Y, Xiao-Ning L, et al. Efficacy of therapy for hepatocellular carcinoma with portal vein tumor thrombus: chemoembolization and stent combined with iodine-125 seed. Cancer Biol Ther 2011;12:865-871.

[157] Li M, He J, Pan M, Yu Y, Pan Z, Xu B, Zhu J. Iodine-125 implantation plus transarterial chemoembolization for the treatment of hepatocellular carcinoma of 3-5cm: A propensity score matching study. Dig Liver Dis 2016;48:1082-1087.

[158] Li N, Feng S, Xue J, Wei XB, Shi J, Guo WX, Lau WY, et al. Hepatocellular carcinoma with main portal vein tumor thrombus: a comparative study comparing hepatectomy with or without neoadjuvant radiotherapy. HPB (Oxford) 2016;18:549-556.

[159] Li WW, Dai ZY, Wan HG, Yao LZ, Zhu J, Li CL, Wang XJ, et al. [Endovascular implantation of iodine-125 seeds strand and portal vein stenting followed by transcatheter arterial chemoembolization combined therapy with sorafenib for hepatocellular carcinoma with main portal vein tumor thrombus]. Zhonghua Yi Xue Za Zhi 2016;96:1838-1842.

[160] Li L, Gou CY, Li JY, Achakzai R, Li XH. Cancer of the Liver Italian Program score helps identify potential candidates for transarterial chemoembolization in patients with Barcelona Clinic Liver Cancer stage C. Hepatobiliary Pancreat Dis Int 2016;15:152-157.

[161] Liang LJ, Hu WJ, Yin XY, Zhou Q, Peng BG, Li DM, Lu MD. Adjuvant intraportal venous chemotherapy for patients with hepatocellular carcinoma and portal vein tumor thrombi following hepatectomy plus portal thrombectomy. World J Surg 2008;32:627-631.

[162] Lim TY, Cheong JY, Cho SW, Sim SJ, Kim JS, Choi SJ, Choi JW, et al. [Effect of low dose 5-fluorouracil and cisplatin intra-arterial infusion chemotherapy in advanced hepatocellular carcinoma with decompensated cirrhosis]. Korean J Hepatol 2006;12:65-73.

[163] Liu PH, Lee YH, Hsia CY, Hsu CY, Huang YH, Chiou YY, Lin HC, et al. Surgical resection versus transarterial chemoembolization for hepatocellular carcinoma with portal vein tumor thrombosis: a propensity score analysis. Ann Surg Oncol 2014;21:1825-1833.

[164] Liu PH, Hsia CY, Lee YH, Hsu CY, Huang YH, Su CW, Lee RC, et al. Surgical resection versus transarterial chemoembolization for BCLC stage C hepatocellular carcinoma. J Surg Oncol 2015;111:404-409.

[165] Lu DH, Fei ZL, Zhou JP, Hu ZT, Hao WS. A comparison between three-dimensional conformal radiotherapy combined with interventional treatment and interventional treatment alone for hepatocellular carcinoma with portal vein tumour thrombosis. J Med Imaging Radiat Oncol 2015;59:109-114.

[166] Luo J, Guo RP, Lai EC, Zhang YJ, Lau WY, Chen MS, Shi M. Transarterial chemoembolization for unresectable hepatocellular carcinoma with portal vein tumor thrombosis: a prospective comparative study. Ann Surg Oncol 2011;18:413-420.

[167] Nagai H, Mukozu T, Ogino YU, Matsui D, Matsui T, Wakui N, Momiyama K, et al. Sorafenib and hepatic arterial infusion chemotherapy for advanced hepatocellular carcinoma with portal vein tumor thrombus. Anticancer Res 2015;35:2269-2277.

[168] Nitta H, Beppu T, Imai K, Hayashi H, Chikamoto A, Baba H. Adjuvant hepatic arterial infusion chemotherapy after hepatic resection of hepatocellular carcinoma with macroscopic vascular invasion. World J Surg 2013;37:1034-1042.

[169] Niu ZJ, Ma YL, Kang P, Ou SQ, Meng ZB, Li ZK, Qi F, et al. Transarterial chemoembolization compared with conservative treatment for advanced hepatocellular carcinoma with portal vein tumor thrombus: using a new classification. Med Oncol 2012;29:2992-2997.

[170] Onishi H, Nouso K, Nakamura S, Katsui K, Wada N, Morimoto Y, Miyahara K, et al. Efficacy of hepatic arterial infusion chemotherapy in combination with irradiation for advanced hepatocellular carcinoma with portal vein invasion. Hepatol Int 2015;9:105-112.

[171] Pinter M, Hucke F, Graziadei I, Vogel W, Maieron A, Konigsberg R, Stauber R, et al. Advanced-stage hepatocellular carcinoma: transarterial chemoembolization versus sorafenib. Radiology 2012;263:590-599.

[172] Radu P, Groza I, Iancu C, Al HN, Andreica V, Sparchez Z. Treatment of hepatocellular carcinoma in a tertiary Romanian center. Deviations from BCLC recommendations and influence on survival rate. J Gastrointestin Liver Dis 2013;22:291-297.

[173] Schmidt L, den op WM, Fischer K, Straub G, Rauch B, Paprottka PM, Goke B, et al. Addition of local hepatic therapy to sorafenib in patients with advanced hepatocellular carcinoma (stage BCLC C). Digestion 2014;90:219-228.

[174] Song DS, Song MJ, Bae SH, Chung WJ, Jang JY, Kim YS, Lee SH, et al. A comparative study between sorafenib and hepatic arterial infusion chemotherapy for advanced hepatocellular carcinoma with portal vein tumor thrombosis. J Gastroenterol 2015;50:445-454.

[175] Tan X, Xie P, Liu J, Wu H, Xie Y. Therapeutic value of transcatheter arterial chemoembolization combined with portal vein embolization for primary hepatocellular carcinoma with portal vein tumor thrombus: a pilot study. Asia Pac J Clin Oncol 2015;11:e6-e12.

[176] Tanaka K, Shimada H, Togo S, Takahashi T, Endo I, Sekido H, Yoshida T. Use of transcatheter arterial infusion of anticancer agents with lipiodol to prevent recurrence of hepatocellular carcinoma after hepatic resection. Hepatogastroenterology 1999;46:1083-1088.

[177] Tang QH, Li AJ, Yang GM, Lai EC, Zhou WP, Jiang ZH, Lau WY, et al. Surgical resection versus conformal radiotherapy combined with TACE for resectable hepatocellular carcinoma with portal vein tumor thrombus: a comparative study. World J Surg 2013;37:1362-1370.

[178] de la Torre MA, Buades-Mateu J, de la Rosa PA, Lue A, Bustamante FJ, Serrano MT, Testillano M, et al. A comparison of survival in patients with hepatocellular carcinoma and portal vein invasion treated by radioembolization or sorafenib. Liver Int 2016;36:1206-1212.

[179] Wang JH, Kuo YH, Wang CC, Chen CL, Cheng YF, Hsu HC, Lu SN. Surgical resection improves the survival of selected hepatocellular carcinoma patients in Barcelona clinic liver cancer stage C. Dig Liver Dis 2013;45:510-515.

[180] Wang CG, Wang XL, Gong GQ, Chen G, Zeng ZC, Qiu WL, Lin GL, et al. [The preliminary study of metallic stent implantation in combination with three-dimensional conformal radiation therapy in the treatment of hepatocellular carcinoma patients with portal vein tumor thrombus]. Zhonghua Gan Zang Bing Za Zhi 2009;17:417-421.

[181] Wu LL, Luo JJ, Yan ZP, Wang JH, Wang XL, Zhang XB, Fang ZT, et al. [Comparative study of portal vein stent and TACE combined therapy with or without endovascular implantation of iodine-125 seeds strand for treating patients with hepatocellular carcinoma and main portal vein tumor thrombus]. Zhonghua Gan Zang Bing Za Zhi 2012;20:915-919.

[182] Wu ZJ, Cai J, Xu AB, Su XQ, Wang XQ, Zhang YX, Shao BF, et al. [Combined three-dimensional conformal radiotherapy plus transcatheter arterial chemoembolization and surgical intervention for portal vein tumor thrombus in patients with hepatocellular carcinoma]. Zhonghua Yi Xue Za Zhi 2011;91:2841-2844.

[183] Xia F, Wu LL, Lau WY, Huan HB, Wen XD, Ma KS, Li XW, et al. Adjuvant sorafenib after heptectomy for Barcelona Clinic Liver Cancer-stage C hepatocellular carcinoma patients. World J Gastroenterol 2016;22:5384-5392.

[184] Xu GB, Xiong B, Long QY. [Curative effect of a comprehensive interventional treatment modality on hepatocellular carcinoma complicated with main branch portal vein tumor thrombosis]. Zhonghua Gan Zang Bing Za Zhi 2013;21:367-371.

[185] Ye JZ, Zhang YQ, Ye HH, Bai T, Ma L, Xiang BD, Li LQ. Appropriate treatment strategies improve survival of hepatocellular carcinoma patients with portal vein tumor thrombus. World J Gastroenterol 2014;20:17141-17147.

[186] Ye HH, Ye JZ, Xie ZB, Peng YC, Chen J, Ma L, Bai T, et al. Comprehensive treatments for hepatocellular carcinoma with tumor thrombus in major portal vein. World J Gastroenterol 2016;22:3632-3643.

[187] You CR, Jang JW, Kang SH, Bae SH, Choi JY, Yoon SK, Choi IB, et al. [Efficacy of transarterial chemolipiodolization with or without 3-dimensional conformal radiotherapy for huge HCC with portal vein tumor thrombosis]. Korean J Hepatol 2007;13:378-386.

[188] Yuan BH, Yuan WP, Li RH, Xiang BD, Gong WF, Li LQ, Zhong JH. Propensity score-based comparison of hepatic resection and transarterial chemoembolization for patients with advanced hepatocellular carcinoma. Tumour Biol 2016;37:2435-2441.

[189] Zhang Y, Fan W, Wang Y, Lu L, Fu S, Yang J, Huang Y, et al. Sorafenib With and Without Transarterial Chemoembolization for Advanced Hepatocellular Carcinoma With Main Portal Vein Tumor Thrombosis: A Retrospective Analysis. Oncologist 2015;20:1417-1424.

[190] Zhang XB, Wang JH, Yan ZP, Qian S, Du SS, Zeng ZC. Hepatocellular carcinoma with main portal vein tumor thrombus: treatment with 3-dimensional conformal radiotherapy after portal vein stenting and transarterial chemoembolization. Cancer 2009;115:1245-1252.

[191] Zhang YF, Wei W, Wang JH, Xu L, Jian PE, Xiao CZ, Zhong XP, et al. Transarterial chemoembolization combined with sorafenib for the treatment of hepatocellular carcinoma with hepatic vein tumor thrombus. Onco Targets Ther 2016;9:4239-4246.

[192] Zhang YF, Guo RP, Zou RH, Shen JX, Wei W, Li SH, OuYang HY, et al. Efficacy and safety of preoperative chemoembolization for resectable hepatocellular carcinoma with portal vein invasion: a prospective comparative study. Eur Radiol 2016;26:2078-2088.

[193] Zheng N, Wei X, Zhang D, Chai W, Che M, Wang J, Du B. Hepatic resection or transarterial chemoembolization for hepatocellular carcinoma with portal vein tumor thrombus. Medicine (Baltimore) 2016;95:e3959.

[194] Zhou Q, Wang Y, Zhou X, Peng B, Yang J, Liang L, Li J. Prognostic analysis for treatment modalities in hepatocellular carcinomas with portal vein tumor thrombi. Asian Pac J Cancer Prev 2011;12:2847-2850.

[195] Andriulli A, de Sio I, Solmi L, De Carlis L, Troisi R, Grasso A, Festa V, et al. Survival of cirrhotic patients with early hepatocellular carcinoma treated by percutaneous ethanol injection or liver transplantation. Liver Transpl 2004;10:1355-1363.

[196] D'Avola D, Inarrairaegui M, Pardo F, Rotellar F, Marti P, Bilbao JI, Martinez-Cuesta A, et al. Prognosis of hepatocellular carcinoma in relation to treatment across BCLC stages. Ann Surg Oncol 2011;18:1964-1971.

[197] Hiraoka A, Kumada T, Michitaka K, Toyoda H, Tada T, Ishikawa T, Itobayashi E, et al. Is there a survival benefit in interventional radiology for hepatocellular carcinoma in patients with Child-Pugh C liver cirrhosis?: A multicenter study. Hepatol Res 2016;46:521-528.

[198] Kudo M, Osaki Y, Matsunaga T, Kasugai H, Oka H, Seki T. Hepatocellular carcinoma in Child-Pugh C cirrhosis: prognostic factors and survival benefit of nontransplant treatments. Dig Dis 2013;31:490-498.

Supplementary Table S2. Quality assessment of included prospective or retrospective cohort based on Newcastle-Ottawa Scale (NOS).

Authors	Pub. Date	Selection			Comparability		Outcome			Score
		Exposed Cohort	Non Exposed Selection	Exposure to Implants	Outcome of Interest	Design or Analysis	Assessment	Adequate Follow-up	Complete Follow-up	
**BCLC Stage 0**										
Dai [1]	2014	*	*	*	*	**	*	*		********
Farinati [2]	2009	*	*	*	*	**	*	*		********
Harada [3]	2016	*	*	*		*	*	*	*	*******
Hasegawa [4]	2012	*	*	*		*	*			*****
Horigome [5]	1999	*	*	*	*	**	*	*	*	*********
HUNG [6]	2011	*	*	*	*	*	*	*		*******
Imai [7]	2013	*	*	*	*	*	*	*	*	********
Kim [8]	2015	*	*			**	*	*	*	*******
Lin [9]	2013	*	*	*	*	*	*	*	*	********
Liu [10]	2015	*	*	*	*	**	*	*		********
Livraghi [11]	2008	*	*		*	*	*	*		******
Pompili [12]	2013	*	*			*	*	*		*****
Pompili [13]	2015	*	*	*		*	*	*	*	*******
Santambrogio [14]	2016	*	*		*	**	*	*	*	********
Takayama [15]	2010	*	*				*	*		****
Takuma [16]	2013	*	*			*	*	*		*****
TASHIRO [17]	2011	*	*		*	**	*	*	*	********
Wahl [18]	2015	*	*	*	*	*	*	*		*******
Wang [19]	2012	*	*		*	*	*	*		******
Zhen [20]	2012	*	*	*	*	*	*	*	*	********
Zhou [21]	2013	*	*	*		*	*	*	*	*******
**BCLC Stage A**										
Abu-Hilal [22]	2008	*	*		*	*	*	*		******
BABA [23]	2010	*	*	*		**	*	*	*	********
Bu [24]	2015	*	*	*	*	*	*	*		*******
Chen [25]	2010	*	*	*	*	**	*	*	*	*********
Chen [26]	2016	*	*	*		**	*	*		*******
Cheung [27]	2012	*	*	*	*	*	*	*		*******
Cho [28]	2007	*	*	*		*	*	*	*	*******
Daniele [29]	2003	*	*	*	*	*	*	*		*******
Desiderio [30]	2013	*	*		*	**	*	*	*	********
Ding [31]	2013	*	*	*	*	**	*	*	*	*********
Farinati [2]	2009	*	*	*	*	**	*	*		********

Gerunda [32]	2000	*	*	*	*	**	*	*	*	*********
Ginsburg [33]	2015	*	*	*	*	**	*	*		********
GORY [34]	2015	*		*	*	*	*	*		******
Gournay [35]	2002	*		*		**	*	*	*	*******
Graham [36]	2013	*	*		*	*	*	*	*	*******
Graham [37]	2013	*	*		*	*	*	*	*	*******
Guan [38]	2016	*	*	*	*	**	*	*		********
Guo [39]	2010	*	*	*	*	*	*	*	*	********
Guo [40]	2014	*	*	*	*	*	*	*	*	********
Guo [41]	2014	*	*	*	*	*	*	*		*******
Harada [3]	2016	*	*		*	*	*	*		******
HARADA [42]	2016	*	*		*	*	*	*		******
Hasegawa [43]	2008	*		*	*	*	*	*		******
Hasegawa [4]	2012	*	*			*	*			****
Hekele [44]	2009	*	*	*	*		*	*	*	*******
Hiraoka [45]	2008	*	*	*	*	*	*	*	*	********
Ho [46]	2009	*	*	*	*	*	*		*	*******
Hocquelet [47]	2015	*			*	*	*	*		*****
Honda [48]	2013	*	*		*	*	*	*	*	*******
Hong [49]	2005	*	*		*	**	*	*		*******
Hsu [50]	2012	*	*		*	**	*	*		*******
Huang [51]	2011	*	*	*	*	*	*	*	*	********
Huang [52]	2014	*	*	*	*	**	*	*	*	*********
HUNG [6]	2011	*	*	*	*	*	*	*		*******
Huo [53]	2003	*	*	*	*	*	*	*		*******
HUO [54]	2004	*	*	*	*	*	*	*	*	********
Hyun [55]	2015	*	*	*	*	**	*	*		********
Jiang [56]	2015	*	*		*	*	*	*		******
Kagawa [57]	2010	*	*	*	*	**	*	*	*	*********
Kamada [58]	2002	*		*	*	**	*	*	*	********
Kang [59]	2015	*	*	*	*	*	*	*		*******
Kim [60]	2014	*	*	*	*	*	*	*		*******
Kim [61]	2006	*	*	*	*	**	*	*	*	*********
Kim [62]	2011	*		*	*	**	*	*		*******
Kim [8]	2015	*	*		*	*	*		*	******
KIM [63]	2012	*	*	*	*		*	*		******
Kim [64]	2013	*	*	*	*	**	*	*	*	*********
Kobayashi [65]	2009	*	*	*	*	*	*	*		*******

Koniaris [66]	2011	*	*		*	*	*		*	******
Lai [67]	2013	*	*		*	**	*	*		*******
LAI [68]	2016	*	*	*	*	**	*	*	*	*********
Lee [69]	2016	*		*	*	*	*		*	******
Lee [70]	2014	*	*	*	*	*	*	*		*******
Lei [71]	2014	*		*	*	**	*	*	*	********
Li [72]	2016	*	*	*	*	*	*	*		*******
LI [73]	2013	*	*		*	**	*	*	*	********
Li [74]	2014	*	*	*	*	*	*	*		*******
Lida [75]	2013	*	*	*	*	**	*		*	********
Liu [76]	2014	*	*		*	*	*		*	******
LUPO [77]	2007	*	*	*	*	**	*	*	*	*********
Montorsi [78]	2005	*	*	*	*	*	*	*		*******
Moon [79]	2007	*	*		*	*	*		*	******
Moreno-Luna [80]	2013	*	*	*	*	*	*	*	*	********
Park [81]	2014	*	*	*	*	*	*	*		*******
Peng [82]	2013	*	*	*	*	**	*	*	*	*********
Pompili [12]	2013	*	*			*	*	*		*****
Potretzke [83]	2016	*	*	*	*	*	*	*	*	********
Santambrogio [84]	2009	*	*	*	*	*	*	*		*******
Shi [85]	2014	*	*	*	*	**	*	*	*	*********
Shi [86]	2016	*	*	*	*	*	*	*		*******
Song [87]	2016	*	*	*	*	**	*	*	*	*********
Song [88]	2015	*	*	*	*	**	*	*		********
Sotiropoulos [89]	2009	*	*	*	*		*	*		******
Takahashi [90]	2007	*	*	*	*	*	*	*	*	********
Takuma [16]	2013	*	*		*	*	*	*		******
TASHIRO [17]	2011	*	*	*	*	*	*	*	*	********
Tohme [91]	2012	*	*	*	*	**	*	*	*	*********
Ueno [92]	2009	*	*	*	*	*	*	*		*******
Vitale [93]	2015	*	*		*		*	*		*****
Vitali [94]	2015	*			*	*	*	*	*	******
Vivarelli [95]	2004	*	*		*	*	*	*		******
Wang [19]	2012	*	*		*	*	*	*		******
Wong [96]	2012	*		*	*	**	*	*	*	********
Yamakado [97]	2008	*	*	*	*	*	*	*		*******
YAN [98]	2016	*	*	*	*		*	*	*	*******
Yang [99]	2014	*	*	*	*	*	*	*	*	********

Yun [100]	2011	*		*	*	*	*	*		******
Zhang [101]	2014	*	*	*	*	*	*	*		*******
Zhang [102]	2014	*		*	*	*	*	*	*	*******
Zhang [103]	2016	*	*	*	*	**	*	*		********
**BCLC Stage B**										
Arizumi [104]	2015	*	*	*	*	**	*	*		********
Azuma [105]	2015	*	*	*	*	**	*	*	*	*********
Ciria [106]	2015	*	*	*	*	*	*	*		*******
Fouly [107]	2015	*	*		*	*	*	*		******
Ginsburg [33]	2015	*	*		*	**	*	*	*	********
Guo [108]	2015	*	*	*	*	*	*	*	*	********
Hekele [44]	2009	*	*	*	*		*	*	*	*******
Ho [109]	2014	*	*	*	*	*			*	******
Ho [46]	2009	*	*	*	*	*	*		*	*******
Jacob [110]	2015	*	*		*	**	*	*	*	********
Jin [111]	2014	*		*	*	**	*	*	*	********
Ke [112]	2014	*	*	*	*	*	*	*		*******
Kim [113]	2016	*	*	*	*	*	*	*	*	********
Lee [114]	2015	*	*	*	*	*	*	*		*******
Lei [115]	2014	*	*	*	*	**	*	*	*	*********
Lin [116]	2010	*	*		*	*	*	*		******
Liu [117]	2016	*	*	*	*	*	*	*		*******
Lubienski [118]	2004	*	*	*	*		*		*	******
Luo [119]	2011	*	*	*	*	*	*	*		*******
Moreno-Luna [80]	2013	*	*	*	*	*	*	*	*	********
NISHIKAWA [120]	2014	*	*	*	*	*	*	*		*******
Pecorelli [121]	2016	*	*		*	*	*	*		******
Shim [122]	2005	*	*	*	*	**	*	*	*	*********
Vitale [93]	2015	*	*		*	*	*	*		******
Yin [123]	2014	*	*	*	*	**	*	*		********
Zhang [102]	2014	*	*	*	*	*	*	*	*	********
Zhao [124]	2010	*	*	*	*	**	*	*	*	*********
Zhong [125]	2014	*	*	*	*	**	*	*		********
Zhong [126]	2013	*	*	*	*	**	*	*	*	*********
**BCLC Stage C**										
Bai [127]	2016	*	*	*	*	**	*	*	*	*********
Chen [128]	2010	*	*	*	*	**	*	*		********
Chen [129]	2014	*	*	*	*	**	*	*	*	*********
Cheng [130]	2005	*	*	*	*	*	*			******

Cheong [131]	2005	*		*	*	**	*	*		*******
Cheung [132]	2014	*	*	*	*	*	*	*	*	********
Cho [133]	2013	*	*		*	*	*	*	*	*******
Cho [134]	2016	*	*		*	*	*	*	*	*******
Choi [135]	2016	*	*	*	*	**	*	*	*	*********
Choi [136]	2013	*	*	*	*	*	*	*		*******
Chung [137]	2011	*	*	*	*	*	*	*		*******
Edeline [138]	2015	*			*		*	*	*	*****
Fan [139]	2005	*	*	*	*	**	*	*		********
Fan [140]	2001	*	*		*	*	*		*	******
Fan [141]	2003	*	*	*	*		*	*		******
Ha [142]	2016	*	*		*	*	*	*		******
Hirooka [143]	2010	*	*	*	*	**	*	*	*	*********
Ho [46]	2009	*	*	*	*	*	*		*	*******
Hu [144]	2014	*	*	*	*	**	*	*	*	*********
Hu [145]	2007	*	*	*	*		*	*		******
Huang [146]	2016	*	*	*	*	**	*	*	*	*********
Jang [147]	2007	*	*	*	*	**	*	*		********
Jin [148]	2003	*	*	*	*		*	*		******
Katamura [149]	2009	*	*	*	*	**	*	*		********
Kawaoka [150]	2015	*	*	*	*	*	*	*	*	********
Kim [151]	2009	*	*	*	*	*	*	*		*******
Kim [152]	2014	*	*	*	*	*	*	*		*******
Kodama [153]	2011	*	*	*	*		*	*		******
Lee [154]	2012	*	*	*	*	*	*	*	*	********
Lee [155]	1997	*	*	*	*	**	*	*		********
Li [156]	2011	*	*	*	*	*	*	*		*******
Li [157]	2016	*	*	*	*	**	*	*	*	*********
Li [158]	2016	*		*	*	*	*	*		******
Li [159]	2016	*	*	*	*	**	*	*	*	*********
Li [160]	2016	*	*	*	*	**	*	*	*	*********
Liang [161]	2008	*		*	*	**	*	*		*******
Lim [162]	2006	*	*		*	**	*			******
Liu [163]	2014	*	*	*	*	*	*	*		*******
Liu [164]	2015	*	*	*	*	*	*	*		*******
Lu [165]	2014	*	*	*	*	**	*	*	*	*********
Luo [166]	2011	*	*		*	*	*	*		******
Moreno-Luna [80]	2013	*	*	*	*	*	*	*	*	********

NAGAI [167]	2015	*	*	*	*	**	*	*		********
Nitta [168]	2013	*	*	*	*	*	*	*		*******
Niu [169]	2012	*	*	*	*	**	*	*		********
Onishi [170]	2015	*	*	*	*	**	*	*	*	*********
Pinter [171]	2012	*	*	*	*	*	*	*		*******
Radu [172]	2013	*	*	*	*		*	*		******
Schmidt [173]	2014	*	*	*	*	**	*	*		********
Song [174]	2014	*	*	*	*	**	*	*	*	*********
TAN [175]	2014	*	*	*	*		*	*		******
Tanaka [176]	1999	*			*	*	*	*	*	******
Tang [177]	2013	*	*	*	*	*	*	*	*	********
Torre [178]	2016	*	*	*	*	**	*	*	*	*********
Vitale [93]	2015	*	*		*		*	*		*****
Wang [179]	2009	*	*	*	*		*	*		******
Wang [180]	2013	*	*		*	*	*	*	*	*******
Wu [181]	2012	*	*	*	*	**	*	*	*	*********
Wu [182]	2011	*	*	*	*		*	*		******
Xia [183]	2016	*	*	*	*	*	*	*		*******
Xu [184]	2013	*	*	*	*	**	*	*		********
Ye [185]	2014	*	*	*	*	**	*	*		********
Ye [186]	2016	*	*	*	*	**	*	*		********
You [187]	2007	*	*		*	**	*		*	*******
Yuan [188]	2016	*	*	*	*	**	*	*	*	*********
ZHANG [189]	2015	*	*	*	*	**	*	*		********
Zhang [190]	2009	*	*	*	*	**	*	*		********
Zhang [192]	2014	*	*	*	*	*	*	*		*******
Zhang [102]	2016	*	*	*	*	**	*	*	*	*********
Zhang [191]	2016	*	*	*	*	**	*	*	*	*********
Zhao [124]	2010	*	*	*	*	**	*	*		********
Zheng [193]	2016	*	*		*	**	*	*		*******
Zhong [125]	2014	*	*	*	*	**	*	*	*	*********
Zhou [194]	2011	*	*	*	*	**	*	*		********
**BCLC Stage D**										
Andriulli [195]	2004	*	*		*	*	*	*	*	*******
D'Avola [196]	2011	*	*	*	*		*	*	*	*******
Hiraoka [197]	2016	*	*	*	*	*	*	*		*******
Kudo [198]	2013	*	*		*		*		*	*****

Supplementary Table S3. Checklist for quality assessment and scoring of prospective or retrospective cohort studies.

Check List

Selection

1. Definitive diagnosis and assignment for treatment: any clear evidence ? (if yes, one star; no star if record linkage or based on self reports or no description)
2. Selection of the non-exposed cohort? (if drawn from the same community as the exposed cohort, one star; no star if drawn from a different source or no description)
3. Ascertainment of exposure to implants? (if secure medical record or structured interview, one star; no star if no description)
4. Demonstration that outcome of interest was not present at start of study. (if yes, one star; no star if no)

Comparability

Comparability of cases and controls on the basis of the design or analysis. (one star was assigned if study controls for BCLC stage and age; one star was assigned if study controls for any additional factor)

Outcome

1. Assessment of outcome? (if independent blind assessment or record linkage, one star)
2. Was follow up long enough for outcomes to occur? (if yes, one star)
3. Adequacy of follow up of cohorts ? (if complete follow up or small number lost < 40 %, or description of those losts, one star)

Supplementary Table S4. Pooled hazard ratios for death.

Stages	Comparisons

BCLC Stage 0. 1-year overall survival

LT	103.01 (0.00, 8.16E+37)	0.00 (0.00, 1.24E+18)	0.00 (0.00, 1.35E+18)	0.00 (0.00, 6.7E+17)	0.00 (0.00, 1.78E+18)	1.02E+7 (0.00, 8.13E+37)
0.01 (0.00, 1.26E+31)	MWA	0.00 (0.00, 1.12E+16)	0.00 (0.00, 1.17E+16)	0.00 (0.00, 6.3E+15)	0.00 (0.00, 1.45E+16)	1090.65 (0.00, 1.03E+45)
9861.78 (0.00, 1.61E+32)	8.22E+7 (0.00, 1.67E+33)	PEI	1.08 (0.32, 3.68)	0.54 (0.04, 8.08)	1.43 (0.41, 6.45)	1.86E+10 (2.32, 2.67E+35)
9493.97 (0.00, 1.40E+32)	7.59E+7 (0.00, 1.73E+33)	0.93 (0.27, 3.16)	RFA	0.50 (0.05, 5.95)	1.34 (0.69, 3.21)	1.71E+10 (2.49, 2.52E+35)
20437.99 (0.00, 2.64E+32)	1.45E+7 (0.00, 3.3E+33)	1.87 (0.12, 24.07)	2.02 (0.17, 19.71)	RT	2.69 (0.28, 25.57)	3.29E+10 (3.87, 5.96E+35)
6965.94 (0.00, 9.92E+32)	5.54E+7 (0.00, 1.17E+33)	0.70 (0.16, 2.45)	0.75 (0.31, 1.45)	0.37 (0.04, 3.52)	SR	1.23E+10 (1.84, 1.88E+35)
0.00 (0.00, 1.98E+23)	0.00 (0.00, 1.53E+25)	0.00 (0.00, 0.43)	0.00 (0.00, 0.40)	0.00 (0.00, 0.26)	0.00 (0.00, 0.54)	TACE+RFA

BCLC Stage 0. 3-year overall survival

LT	0.17 (0.00, 8.06)	0.59 (0.01, 14.99)	0.54 (0.01, 11.90)	0.25 (0.00, 10.09)	0.77 (0.02, 16.84)	0.12 (0.00, 5.70)
6.02 (0.12, 508.65)	MWA	3.38 (0.42, 31.56)	3.13 (0.32, 34.64)	1.51 (0.08, 30.56)	4.44 (0.44, 49.86)	0.73 (0.03, 19.55)
1.70 (0.07, 94.59)	0.30 (0.03, 2.38)	PEI	0.92 (0.40, 2.16)	0.44 (0.05, 3.37)	1.30 (0.52, 3.50)	0.22 (0.02, 2.16)
1.84 (0.08, 96.77)	0.32 (0.03, 3.13)	1.08 (0.46, 2.53)	RFA	0.48 (0.07, 3.18)	1.42 (0.87, 2.37)	0.24 (0.02, 2.05)
4.03 (0.10, 284.91)	0.66 (0.03, 13.24)	2.26 (0.30, 19.64)	2.07 (0.31, 15.14)	RT	2.94 (0.49, 20.27)	0.51 (0.02, 8.37)
1.29 (0.06, 63.14)	0.23 (0.02, 2.26)	0.77 (0.29, 1.93)	0.71 (0.42, 1.15)	0.34 (0.05, 2.04)	SR	0.17 (0.02, 1.32)
8.14 (0.18, 718.05)	1.36 (0.05, 34.67)	4.51 (0.46, 57.51)	4.16 (0.49, 47.28)	1.98 (0.12, 40.24)	5.92 (0.76, 65.36)	TACE+RFA

BCLC Stage 0. 5-year overall survival

LT	0.25 (0.01, 3.98)	0.13 (0.00, 1.73)	0.13 (0.00, 7.29)	0.04 (0.00, 1.96)	0.24 (0.01, 3.09)	0.04 (0.00, 0.80)	0.04 (0.00, 1.13)
4.03 (0.25, 181.91)	PEI	0.52 (0.20, 1.42)	0.60 (0.01, 12.89)	0.15 (0.00, 5.47)	0.95 (0.33, 2.82)	0.15 (0.01, 2.26)	0.17 (0.02, 1.58)
7.62 (0.58, 305.20)	1.92 (0.71, 5.07)	RFA	1.14 (0.03, 20.80)	0.30 (0.00, 9.50)	1.82 (1.04, 3.23)	0.29 (0.02, 3.64)	0.33 (0.04, 2.59)
7.51 (0.14, 1152.93)	1.66 (0.08, 73.36)	0.88 (0.05, 33.54)	RT	0.28 (0.00, 34.73)	1.57 (0.10, 58.96)	0.26 (0.01, 18.98)	0.29 (0.01, 19.91)
28.41 (0.51, 4557.71)	6.50 (0.18, 472.48)	3.37 (0.11, 226.56)	3.62 (0.03, 642.71)	SC	6.18 (0.21, 407.38)	0.96 (0.04, 51.93)	1.12 (0.02, 108.56)
4.15 (0.32, 164.24)	1.05 (0.35, 3.07)	0.55 (0.31, 0.96)	0.64 (0.02, 10.34)	0.16 (0.00, 4.67)	SR	0.16 (0.01, 1.81)	0.18 (0.03, 1.29)
26.72 (1.25, 1548.02)	6.57 (0.44, 102.85)	3.45 (0.27, 44.40)	3.81 (0.05, 177.16)	1.04 (0.02, 28.05)	6.35 (0.55, 80.25)	TACE	1.17 (0.05, 27.95)
24.00 (0.88, 1390.55)	5.78 (0.63, 53.21)	3.03 (0.39, 24.32)	3.42 (0.05, 109.18)	0.89 (0.01, 43.56)	5.52 (0.77, 39.57)	0.86 (0.04, 18.88)	TACE +RFA

BCLC Stage A. 1-year overall survival

HIFU	3.25E+13 (1.63, 1.90E+57)	0.57 (0.01, 9.38)	0.24 (0.00, 8.39)	0.32 (0.01, 5.30)	1.09 (0.02, 40.25)	0.00 (0.00, 5.35*10^{20})	0.46 (0.01, 7.10)	0.33 (0.01, 4.61)	0.68 (0.01, 13.43)	0.39 (0.01, 9.87)	0.06 (0.00, 0.89)	0.39 (0.01, 5.71)	0.29 (0.00, 11.46)	0.34 (0.01, 4.96)	1.52 (0.02, 41.87)	0.73 (0.01, 23.44)	0.67 (0.02, 10.67)	0.30 (0.01, 6.92)	1.01 (0.02, 33.37)	0.15 (0.00, 4.61)	7.97E+12 (4.42, 5.29E+63)
0.00 (0.00, 0.61)	HIFU+ TACE	0.00 (0.00, 0.32)	0.00 (0.00, 0.18)	0.00 (0.00, 0.19)	0.00 (0.00, 0.69)	0.00 (0.00, 1.31E+13)	0.00 (0.00, 0.25)	0.00 (0.00, 0.19)	0.00 (0.00, 0.43)	0.00 (0.00, 0.25)	0.00 (0.00, 0.03)	0.00 (0.00, 0.22)	0.00 (0.00, 0.17)	0.00 (0.00, 0.19)	0.00 (0.00, 1.11)	0.00 (0.00, 0.47)	0.00 (0.00, 0.38)	0.00 (0.00, 0.21)	0.00 (0.00, 0.77)	0.00 (0.00, 0.10)	33.24 (0.00, 1.47E+21)
1.75 (0.11, 74.06)	8.26E+13 (3.08, 2.30E+57)	LT	0.44 (0.04, 3.49)	0.54 (0.17, 1.73)	1.94 (0.22, 20.91)	0.00 (0.00, 6.65E+20)	0.79 (0.30, 2.19)	0.57 (0.25, 1.30)	1.17 (0.25, 5.53)	0.70 (0.11, 4.49)	0.10 (0.03, 0.32)	0.69 (0.31, 1.53)	0.52 (0.05, 6.97)	0.57 (0.23, 1.44)	2.65 (0.31, 24.63)	1.29 (0.14, 12.19)	1.14 (0.41, 3.38)	0.53 (0.08, 3.67)	1.83 (0.22, 17.09)	0.27 (0.03, 2.40)	1.42E+12 (11.62, 1.04E+63)
4.12 (0.12, 282.86)	1.99E+13 (5.70, 4.60E+57)	2.25 (0.29, 23.51)	LT+TACE	1.22 (0.12, 15.98)	4.48 (0.23, 124.57)	0.00 (0.00, 1.34E+20)	1.77 (0.18, 22.50)	1.27 (0.14, 14.55)	2.67 (0.21, 41.62)	1.55 (0.09, 28.90)	0.22 (0.02, 2.93)	1.54 (0.17, 17.64)	1.23 (0.05, 33.46)	1.28 (0.14, 15.44)	6.13 (0.30, 137.25)	2.94 (0.14, 71.37)	2.56 (0.26, 31.59)	1.19 (0.07, 23.84)	4.09 (0.22, 103.44)	0.61 (0.03, 14.26)	3.28E+12 (26.44, 246E+63)
3.14 (0.19, 137.12)	1.64E+13 (5.22, 4.52E+57)	1.84 (0.58, 5.83)	0.82 (0.06, 8.54)	MWA	3.55 (0.41, 39.18)	0.00 (0.00, 1.27E+20)	1.45 (0.52, 4.13)	1.04 (0.45, 2.40)	2.17 (0.46, 10.03)	1.28 (0.18, 8.46)	0.18 (0.05, 0.60)	1.25 (0.54, 2.90)	0.93 (0.08, 12.97)	1.05 (0.41, 2.72)	4.89 (0.56, 44.76)	2.35 (0.26, 24.40)	2.11 (0.72, 6.35)	0.96 (0.14, 7.09)	3.36 (0.39, 30.85)	0.49 (0.06, 4.63)	2.67E+12 (21.46, 198E+63)
0.91 (0.02, 59.22)	4.09E+13 (1.46, 1.44E+57)	0.52 (0.05, 4.47)	0.22 (0.01, 4.30)	0.28 (0.03, 2.42)	PAI	0.00 (0.00, 4.10E+20)	0.41 (0.04, 3.38)	0.30 (0.03, 2.13)	0.60 (0.04, 6.75)	0.36 (0.02, 4.71)	0.05 (0.00, 0.41)	0.36 (0.04, 2.60)	0.27 (0.01, 6.22)	0.30 (0.03, 2.07)	1.37 (0.07, 24.22)	0.66 (0.03, 12.41)	0.60 (0.06, 4.82)	0.27 (0.02, 3.50)	0.93 (0.05, 17.41)	0.14 (0.01, 2.36)	6.54E+11 (5.69, 513E+63)
5.02E+3 (0.00, 4.82E+21)	2.12E+23 (0.00, 3.86E+59)	1.70E+3 (0.00, 2.15E+21)	9.58E+3 (0.00, 1.13E+21)	9.83E+3 (0.00, 1.15E+21	3.38E+4 (0.00, 3.86E+21	PC	1.41E+4 (0.00, 1.67E+21)	1.08E+4 (0.00, 1.09E+21)	2.11E+4 (0.00, 2.16E+21)	1.20E+4 (0.00, 1.99E+21)	1.78E+3 (0.00, 1.90E+20)	1.25E+4 (0.00, 1.30E+21	1.06E+4 (0.00, 9.82E+20	1.06E+4 (0.00, 1.04E+21)	4.92E+4 (0.00, 4.44E+21)	2.20E+4 (0.00, 3.45E+21)	2.07E+4 (0.00, 2.12E+21)	9.16E+3 (0.00, 1.44E+21)	3.20E+4 (0.00, 3.03E+21)	5.14E+3 (0.00, 6.97E+20)	3.56E+22 (0.00, 2.72E+68)

				)	)							)	)								
2.19 (0.14, 87.35)	1.07E+14 (4.02, 3.11E+57)	1.26 (0.46, 3.38)	0.57 (0.04, 5.41)	0.69 (0.24, 1.91)	2.41 (0.30, 24.85)	0.00 (0.00, 8.30E+20)	PEI	0.72 (0.37, 1.34)	1.49 (0.34, 6.36)	0.87 (0.14, 5.19)	0.12 (0.04, 0.35)	0.86 (0.46, 1.57)	0.64 (0.06, 8.49)	0.72 (0.33, 1.53)	3.39 (0.41, 28.46)	1.62 (0.23, 12.44)	1.45 (0.58, 3.63)	0.66 (0.10, 4.33)	2.30 (0.30, 19.57)	0.34 (0.04, 2.90)	1.82E+12 (14.07, 1.30E+62)
3.06 (0.22, 118.17)	1.44E+14 (5.26, 3.88E+57)	1.76 (0.77, 4.01)	0.79 (0.07, 7.14)	0.96 (0.42, 2.21)	3.38 (0.47, 33.03)	0.00 (0.00, 1.23E+20)	1.40 (0.74, 2.72)	RFA	2.08 (0.57, 7.57)	1.21 (0.21, 6.73)	0.17 (0.07, 0.43)	1.21 (0.93, 1.57)	0.89 (0.09, 11.14)	1.01 (0.62, 1.67)	4.70 (0.64, 38.18)	2.28 (0.29, 19.38)	2.03 (1.00, 4.21)	0.93 (0.16, 5.46)	3.19 (0.45, 26.41)	0.47 (0.06, 3.90)	2.58E+12 (20.29, 1.92E+63)
1.47 (0.07, 72.07)	7.36E+14 (2.31, 1.92E+57)	0.86 (0.18, 4.02)	0.37 (0.02, 4.85)	0.46 (0.10, 2.19)	1.66 (0.15, 22.76)	0.00 (0.00, 6.41E+20)	0.67 (0.16, 2.92)	0.48 (0.13, 1.76)	RFA+PEI	0.59 (0.07, 4.85)	0.08 (0.02, 0.40)	0.58 (0.15, 2.18)	0.44 (0.03, 7.66)	0.49 (0.12, 2.05)	2.29 (0.21, 25.82)	1.09 (0.09, 14.27)	0.98 (0.22, 4.35)	0.45 (0.05, 3.93)	1.57 (0.15, 17.59)	0.23 (0.02, 2.57)	1.32E+12.51 (9.79, 9.86E+63)
2.58 (0.10, 132.30)	1.37E+14 (3.92, 2.65E+57)	1.43 (0.22, 9.47)	0.65 (0.03, 11.19)	0.78 (0.12, 5.47)	2.78 (0.21, 48.62)	0.00 (0.00, 9.28E+20)	1.14 (0.19, 7.27)	0.83 (0.15, 4.66)	1.69 (0.21, 14.77)	RFA+Sorafenib	0.14 (0.02, 0.96)	1.00 (0.18, 5.43)	0.75 (0.04, 15.62)	0.83 (0.15, 4.89)	3.94 (0.29, 56.67)	1.92 (0.13, 28.20)	1.66 (0.27, 10.81)	0.77 (0.07, 8.79)	2.65 (0.20, 36.28)	0.39 (0.03, 5.73)	2.03E+12 (15.57, 1.67E+63)
18.13 (1.13, 774.46)	8.98E+14 (35.01, 215E+57)	10.28 (3.17, 34.26)	4.61 (0.34, 48.89)	5.62 (1.67, 19.72)	19.86 (2.41, 231.53)	0.00 (0.00, 7.03E+20)	8.13 (2.83, 25.29)	5.80 (2.34, 15.01)	11.97 (2.51, 64.80)	7.09 (1.05, 49.22)	SC	7.00 (2.97, 17.69)	5.25 (0.49, 78.00)	5.89 (2.33, 15.32)	27.54 (3.15, 264.61)	13.26 (1.45, 133.60)	11.88 (3.94, 37.54)	5.46 (0.78, 38.16)	18.59 (2.23, 181.45)	2.71 (0.33, 27.55)	1.38E+13 (109.55, 1.05E+64)
2.55 (0.18, 101.48)	1.23E+14 (4.47, 3.13E+557)	1.46 (0.66, 3.23)	0.65 (0.06, 5.79)	0.80 (0.34, 1.86)	2.80 (0.38, 27.84)	0.00 (0.00, 9.93E+20)	1.16 (0.64, 2.19)	0.83 (0.64, 1.07)	1.72 (0.46, 6.55)	1.00 (0.18, 5.46)	0.14 (0.06, 0.34)	SR	0.74 (0.08, 9.13)	0.84 (0.52, 1.35)	3.91 (0.53, 30.71)	1.89 (0.24, 16.26)	1.68 (0.84, 3.44)	0.77 (0.13, 4.48)	2.65 (0.39, 21.41)	0.39 (0.05, 3.16)	2.01E+12 (17.23, 1.54E+63)
3.44 (0.09, 256.74)	1.69E+14 (5.94, 3.80E+57)	1.94 (0.14, 22.01)	0.81 (0.03, 19.97)	1.08 (0.08, 11.90)	3.77 (0.16, 89.79)	0.00 (0.00, 1.15E+21)	1.57 (0.12, 16.19)	1.12 (0.09, 10.86)	2.28 (0.13, 31.86)	1.33 (0.06, 22.71)	0.19 (0.01, 2.06)	1.35 (0.11, 12.85)	SR+RFA	1.13 (0.09, 10.77)	5.20 (0.22, 112.97)	2.59 (0.10, 53.63)	2.24 (0.17, 23.89)	1.02 (0.05, 17.35)	3.58 (0.14, 74.28)	0.52 (0.02, 11.60)	2.68E+12 (21.47, 2.48E+63)
2.98 (0.20, 126.52)	1.39E+14 (5.21, 3.72E+57)	1.74 (0.69, 4.35)	0.78 (0.06, 7.22)	0.95 (0.37, 2.47)	3.33 (0.48, 31.97)	0.00 (0.00, 1.26E+21)	1.38 (0.66, 3.04)	0.99 (0.60, 1.63)	2.06 (0.49, 8.26)	1.20 (0.20, 6.88)	0.17 (0.07, 0.43)	1.19 (0.74, 1.93)	0.89 (0.09, 10.92)	TACE	4.64 (0.61, 38.88)	2.24 (0.27, 19.73)	2.02 (0.93, 4.37)	0.92 (0.17, 5.10)	3.19 (0.42, 26.26)	0.47 (0.07, 3.62)	2.68E+12 (21.20, 1.82E+63)
0.66 (0.02, 43.08)	3.52E+13 (0.90, 6.85E+57)	0.38 (0.04, 3.23)	0.16 (0.01, 3.29)	0.20 (0.02, 1.79)	0.73 (0.04, 14.94)	0.00 (0.00, 2.59E+20)	0.29 (0.04, 2.46)	0.21 (0.03, 1.56)	0.44 (0.04, 4.83)	0.25 (0.02, 3.48)	0.04 (0.00, 0.32)	0.26 (0.03, 1.88)	0.19 (0.01, 4.62)	0.22 (0.03, 1.63)	TACE+MWA	0.47 (0.03, 8.70)	0.43 (0.06, 2.75)	0.19 (0.01, 2.73)	0.69 (0.04, 11.76)	0.10 (0.01, 1.77)	5.91E+12 (3.98, 4.22E+63)
1.38 (0.04, 104.31)	6.83E+13 (2.14, 2.27E+57)	0.77 (0.08, 7.10)	0.34 (0.01, 7.31)	0.43 (0.04, 3.82)	1.50 (0.08, 31.94)	0.00 (0.00, 5.46E+20)	0.62 (0.08, 4.36)	0.44 (0.05, 3.45)	0.92 (0.07, 10.59)	0.52 (0.04, 7.84)	0.08 (0.01, 0.69)	0.53 (0.06, 4.19)	0.39 (0.02, 10.11)	0.45 (0.05, 3.66)	2.11 (0.11, 36.50)	TACE+PEI	0.89 (0.09, 7.82)	0.41 (0.02, 6.03)	1.44 (0.08, 25.11)	0.21 (0.01, 3.76)	9.93E+12 (7.90, 8.30E+63)
1.50 (0.09, 62.48)	7.45E+13 (2.63, 1.88E+57)	0.88 (0.30, 2.45)	0.39 (0.03, 3.86)	0.47 (0.16, 1.39)	1.66 (0.21, 18.00)	0.00 (0.00, 6.31E+20)	0.69 (0.28, 1.72)	0.49 (0.24, 1.00)	1.02 (0.23, 4.53)	0.60 (0.09, 3.73)	0.08 (0.03, 0.25)	0.60 (0.29, 1.18)	0.45 (0.04, 5.99)	0.50 (0.23, 1.07)	2.31 (0.36, 15.50)	1.12 (0.13, 10.84)	TACE+RFA	0.46 (0.07, 2.99)	1.60 (0.19, 14.27)	0.23 (0.03, 2.03)	1.27E+13 (10.61, 9.41E+63)
3.36 (0.14, 192.80)	1.62E+14 (4.69, 4.04E+57)	1.88 (0.27, 13.20)	0.84 (0.04, 13.35)	1.05 (0.14, 7.32)	3.70 (0.29, 56.96)	0.00 (0.00, 1.18E+21)	1.51 (0.23, 10.00)	1.07 (0.18, 6.35)	2.20 (0.25, 21.29)	1.30 (0.11, 14.78)	0.18 (0.03, 1.28)	1.29 (0.22, 7.55)	0.98 (0.06, 20.74)	1.09 (0.20, 6.04)	5.13 (0.37, 76.44)	2.43 (0.17, 40.22)	2.19 (0.33, 14.22)	TACE+RT	3.53 (0.24, 50.41)	0.52 (0.04, 7.18)	3.47E+12 (20.09, 2.02E+63)
0.99 (0.03, 63.60)	4.36E+13 (1.30, 1.29E+57)	0.55 (0.06, 4.54)	0.24 (0.01, 4.60)	0.30 (0.03, 2.59)	1.08 (0.06, 20.47)	0.00 (0.00, 4.76E+20)	0.43 (0.05, 3.36)	0.31 (0.04, 2.21)	0.64 (0.06, 6.76)	0.38 (0.03, 5.03)	0.05 (0.01, 0.45)	0.38 (0.05, 2.57)	0.28 (0.01, 6.98)	0.31 (0.04, 2.36)	1.45 (0.09, 26.08)	0.70 (0.04, 12.48)	0.63 (0.07, 5.14)	0.28 (0.02, 4.13)	TACE+SR	0.14 (0.01, 2.53)	7.64E+11 (4.92, 5.22E+63)
6.70 (0.22,	3.47E+13	3.72 (0.42,	1.63 (0.07,	2.03 (0.22	7.26 (0.42	0.00 (0.00,	2.96 (0.34,	2.11 (0.26,	4.31 (0.39,	2.57 (0.17,	0.37 (0.04,	2.57 (0.32	1.91 (0.09	2.13 (0.28,	9.88 (0.57,	4.75 (0.27,	4.31 (0.49,	1.94 (0.14,	6.92 (0.40,	TARE	6.79E+11

452.32)	(9.54, 7.70E+57)	32.22)	35.73)	, 17.30)	, 131.38)	3.21E+21)	24.49)	15.83)	47.75)	35.10)	2.99)	, 18.52)	, 43.72)	15.18)	169.51)	90.62)	34.11)	25.84)	120.33)		(43.04, 3.48E+63)
0.00 (0.00, 0.23)	0.03 (0.00, 1.11E+31)	0.00 (0.00, 0.09)	0.00 (0.00, 0.04)	0.00 (0.00, 0.05)	0.00 (0.00, 0.18)	0.00 (0.00, 8.57E+16)	0.00 (0.00, 0.07)	0.00 (0.00, 0.05)	0.00 (0.00, 0.10)	0.00 (0.00, 0.06)	0.00 (0.00, 0.01)	0.00 (0.00, 0.06)	0.00 (0.00, 0.05)	0.00 (0.00, 0.05)	0.00 (0.00, 0.25)	0.00 (0.00, 0.13)	0.00 (0.00, 0.09)	0.00 (0.00, 0.05)	0.00 (0.00, 0.20)	0.00 (0.00, 0.02)	Tace+PAI

BCLC Stage A. 3-year overall survival

HIFU	6.25 (1.36, 31.27)	3.71 (0.37, 38.00)	1.18 (0.27, 5.56)	2.04 (0.30, 13.68)	2.20 (0.27, 17.62)	1.98 (0.43, 9.69)	1.49 (0.37, 6.06)	2.55 (0.43, 15.53)	2.43 (0.35, 15.73)	0.30 (0.06, 1.60)	2.40 (0.58, 10.05)	1.68 (0.18, 14.99)	1.38 (0.33, 6.12)	6.28 (0.85, 53.13)	5.22 (0.65, 42.31)	1.75 (0.40, 8.28)	0.00 (0.00, 0.29)	3.10 (0.51, 19.36)	2.17 (0.16, 28.12)	9.16 (0.90, 93.44)
0.16 (0.03, 0.73)	LT	0.59 (0.11, 3.16)	0.19 (0.08, 0.44)	0.32 (0.08, 1.33)	0.34 (0.07, 1.87)	0.32 (0.13, 0.74)	0.24 (0.12, 0.45)	0.41 (0.11, 1.47)	0.39 (0.09, 1.57)	0.05 (0.02, 0.14)	0.39 (0.20, 0.72)	0.26 (0.04, 1.68)	0.22 (0.11, 0.45)	1.00 (0.19, 5.31)	0.84 (0.16, 4.35)	0.28 (0.12, 0.62)	0.00 (0.00, 0.04)	0.49 (0.14, 1.78)	0.35 (0.03, 3.20)	1.46 (0.21, 9.84)
0.27 (0.03, 2.68)	1.69 (0.32, 9.38)	LT+TACE	0.32 (0.05, 2.19)	0.55 (0.06, 5.04)	0.60 (0.06, 6.65)	0.54 (0.08, 3.82)	0.40 (0.07, 2.51)	0.68 (0.08, 6.10)	0.65 (0.07, 6.08)	0.08 (0.01, 0.58)	0.65 (0.11, 4.01)	0.45 (0.04, 5.81)	0.37 (0.06, 2.48)	1.74 (0.16, 18.02)	1.44 (0.13, 15.67)	0.48 (0.08, 3.12)	0.00 (0.00, 0.08)	0.84 (0.10, 7.06)	0.59 (0.03, 10.05)	2.53 (0.18, 32.46)
0.85 (0.18, 3.66)	5.22 (2.29, 12.15)	3.13 (0.46, 20.34)	MWA	1.69 (0.43, 6.88)	1.81 (0.36, 9.77)	1.68 (0.76, 3.75)	1.25 (0.72, 2.17)	2.15 (0.60, 7.27)	2.02 (0.50, 8.04)	0.25 (0.09, 0.69)	2.02 (1.17, 3.53)	1.41 (0.23, 8.60)	1.16 (0.61, 2.26)	5.30 (1.05, 27.64)	4.38 (0.86, 22.47)	1.48 (0.71, 3.06)	0.00 (0.00, 0.21)	2.58 (0.76, 9.07)	1.85 (0.19, 16.69)	7.61 (1.12, 52.85)
0.49 (0.07, 3.31)	3.09 (0.75, 12.60)	1.83 (0.20, 16.79)	0.59 (0.15, 2.34)	PAI	1.08 (0.15, 8.04)	0.99 (0.24, 3.99)	0.74 (0.20, 2.64)	1.27 (0.23, 6.70)	1.19 (0.20, 7.25)	0.15 (0.03, 0.66)	1.20 (0.33, 4.30)	0.83 (0.10, 7.12)	0.69 (0.20, 2.30)	3.15 (0.43, 22.66)	2.62 (0.36, 19.15)	0.89 (0.22, 3.31)	0.00 (0.00, 0.12)	1.53 (0.28, 8.41)	1.08 (0.09, 11.77)	4.49 (1.20, 17.02)
0.45 (0.06, 3.74)	2.92 (0.53, 15.09)	1.68 (0.15, 17.61)	0.55 (0.10, 2.76)	0.92 (0.12, 6.86)	PC	0.91 (0.17, 4.64)	0.69 (0.14, 3.14)	1.17 (0.17, 7.86)	1.10 (0.14, 8.22)	0.14 (0.02, 0.77)	1.11 (0.23, 5.07)	0.77 (0.07, 7.84)	0.64 (0.13, 3.06)	2.93 (0.32, 26.68)	2.47 (0.27, 20.51)	0.81 (0.15, 4.12)	0.00 (0.00, 0.12)	1.42 (0.20, 9.76)	1.01 (0.07, 13.70)	4.16 (0.36, 47.41)
0.51 (0.10, 2.35)	3.12 (1.36, 7.60)	1.85 (0.26, 12.12)	0.60 (0.27, 1.32)	1.01 (0.25, 4.13)	1.10 (0.22, 5.81)	PEI	0.75 (0.40, 1.38)	1.29 (0.36, 4.48)	1.20 (0.30, 4.91)	0.15 (0.05, 0.43)	1.20 (0.67, 2.20)	0.83 (0.13, 5.20)	0.69 (0.35, 1.42)	3.15 (0.64, 16.49)	2.64 (0.64, 10.66)	0.89 (0.41, 1.93)	0.00 (0.00, 0.12)	1.55 (0.43, 5.48)	1.09 (0.12, 9.68)	4.56 (0.67, 31.08)
0.67 (0.17, 2.72)	4.17 (2.21, 8.32)	2.48 (0.40, 15.05)	0.80 (0.46, 1.39)	1.34 (0.38, 4.92)	1.46 (0.32, 7.11)	1.34 (0.72, 2.48)	RFA	1.71 (0.56, 5.21)	1.62 (0.43, 5.77)	0.20 (0.08, 0.48)	1.61 (1.32, 1.98)	1.11 (0.19, 6.26)	0.93 (0.63, 1.41)	4.23 (0.92, 20.21)	3.53 (0.74, 16.42)	1.19 (0.72, 1.98)	0.00 (0.00, 0.17)	2.06 (0.67, 6.53)	1.47 (0.17, 12.07)	6.10 (0.99, 39.04)
0.39 (0.06, 2.33)	2.43 (0.68, 9.17)	1.46 (0.16, 11.92)	0.46 (0.14, 1.67)	0.78 (0.15, 4.27)	0.85 (0.13, 5.93)	0.77 (0.22, 2.79)	0.58 (0.19, 1.78)	RFA+PEI	0.93 (0.17, 5.14)	0.12 (0.03, 0.48)	0.94 (0.31, 2.90)	0.66 (0.08, 5.13)	0.54 (0.17, 1.77)	2.51 (0.36, 16.60)	2.05 (0.31, 13.81)	0.68 (0.21, 2.31)	0.00 (0.00, 0.11)	1.20 (0.25, 5.97)	0.86 (0.07, 9.41)	3.53 (0.42, 29.80)
0.41 (0.06, 2.86)	2.59 (0.64, 10.95)	1.53 (0.16, 14.15)	0.50 (0.12, 2.00)	0.84 (0.14, 4.91)	0.91 (0.12, 6.93)	0.83 (0.20, 3.37)	0.62 (0.17, 2.31)	1.08 (0.19, 5.81)	RFA+Sorafenib	0.13 (0.03, 0.56)	1.00 (0.28, 3.69)	0.69 (0.08, 6.07)	0.58 (0.16, 2.18)	2.66 (0.36, 19.67)	2.20 (0.29, 15.87)	0.73 (0.19, 2.90)	0.00 (0.00, 0.11)	1.29 (0.23, 6.93)	0.90 (0.07, 10.97)	3.83 (0.42, 35.60)
3.31 (0.63, 17.95)	20.74 (7.39, 63.66)	12.20 (1.72, 91.89)	3.93 (1.45, 11.44)	6.69 (1.51, 31.92)	7.18 (1.30, 45.70)	6.62 (2.35, 19.42)	4.95 (2.07, 12.37)	8.47 (2.07, 36.27)	7.99 (1.77, 36.64)	SC	8.00 (3.47, 19.87)	5.47 (0.78, 40.03)	4.58 (1.92, 11.81)	20.88 (3.85, 127.01)	17.44 (2.92, 103.77)	5.86 (2.25, 16.09)	0.00 (0.00, 0.85)	10.11 (2.55, 45.02)	7.29 (0.71, 71.73)	30.18 (4.04, 240.23)
0.42 (0.10, 1.72)	2.58 (1.39, 4.95)	1.53 (0.25, 9.25)	0.49 (0.28, 0.85)	0.83 (0.23, 3.02)	0.90 (0.20, 4.35)	0.83 (0.46, 1.49)	0.62 (0.50, 0.75)	1.07 (0.34, 3.20)	1.00 (0.27, 3.51)	0.13 (0.05, 0.29)	SR	0.69 (0.12, 3.85)	0.58 (0.39, 0.85)	2.62 (0.57, 12.40)	2.18 (0.47, 10.08)	0.73 (0.45, 1.20)	0.00 (0.00, 0.10)	1.28 (0.41, 3.95)	0.91 (0.10, 7.51)	3.78 (0.60, 23.37)
0.59 (0.07, 5.58)	3.78 (0.59, 24.52)	2.24 (0.17, 26.48)	0.71 (0.12, 4.34)	1.20 (0.14, 10.20)	1.30 (0.13, 14.13)	1.20 (0.19, 7.63)	0.90 (0.16, 5.17)	1.52 (0.20, 11.88)	1.45 (0.16, 12.54)	0.18 (0.02, 1.28)	1.45 (0.26, 8.34)	SR+RFA	0.84 (0.14, 4.93)	3.82 (0.37, 38.36)	3.16 (0.32, 33.21)	1.06 (0.17, 6.54)	0.00 (0.00, 0.16)	1.85 (0.24, 14.20)	1.32 (0.08, 18.89)	5.48 (0.45, 65.65)
0.72 (0.16, 3.04)	4.48 (2.21, 9.52)	2.69 (0.40, 16.87)	0.86 (0.44, 1.64)	1.45 (0.43, 4.93)	1.56 (0.33, 7.65)	1.45 (0.71, 2.88)	1.08 (0.71, 1.59)	1.85 (0.57, 6.01)	1.74 (0.46, 6.40)	0.22 (0.08, 0.52)	1.74 (1.18, 2.55)	1.20 (0.20, 6.95)	TACE	4.51 (0.97, 22.06)	3.78 (0.75, 18.58)	1.27 (0.71, 2.25)	0.00 (0.00, 0.18)	2.23 (0.69, 7.20)	1.58 (0.18, 12.52)	6.56 (1.08, 38.74)
0.16 (0.02, 1.18)	1.00 (0.19, 5.21)	0.57 (0.06, 6.15)	0.19 (0.04, 0.95)	0.32 (0.04, 2.31)	0.34 (0.04, 3.11)	0.32 (0.06, 1.57)	0.24 (0.05, 1.09)	0.40 (0.06, 2.75)	0.38 (0.05, 2.79)	0.05 (0.01, 0.26)	0.38 (0.08, 1.75)	0.26 (0.03, 2.71)	0.22 (0.05, 1.03)	TACE+MWA	0.81 (0.10, 7.45)	0.28 (0.06, 1.20)	0.00 (0.00, 0.05)	0.49 (0.07, 3.26)	0.34 (0.02, 4.78)	1.43 (0.13, 16.18)
0.19 (0.02, 1.53)	1.19 (0.23, 6.39)	0.70 (0.06, 7.54)	0.23 (0.04, 1.16)	0.38 (0.05, 2.80)	0.40 (0.05, 3.75)	0.38 (0.09, 1.56)	0.28 (0.06, 1.35)	0.49 (0.07, 3.24)	0.45 (0.06, 3.43)	0.06 (0.01, 0.34)	0.46 (0.10, 2.15)	0.32 (0.03, 3.11)	0.26 (0.05, 1.33)	1.23 (0.13, 10.36)	TACE+PEI	0.34 (0.07, 1.69)	0.00 (0.00, 0.05)	0.59 (0.09, 3.83)	0.41 (0.03, 5.75)	1.75 (0.16, 18.51)

0.57 (0.12, 2.52)	3.52 (1.62, 8.01)	2.09 (0.32, 13.17)	0.68 (0.33, 1.41)	1.13 (0.30, 4.55)	1.23 (0.24, 6.46)	1.13 (0.52, 2.42)	0.84 (0.51, 1.39)	1.47 (0.43, 4.86)	1.37 (0.34, 5.15)	0.17 (0.06, 0.45)	1.36 (0.83, 2.23)	0.94 (0.15, 5.74)	0.78 (0.44, 1.41)	3.59 (0.83, 15.53)	2.97 (0.59, 14.92)	TACE+RFA	0.00 (0.00, 0.15)	1.74 (0.51, 5.95)	1.24 (0.13, 10.48)	5.12 (0.76, 34.65)
2.04E+10 (3.43, 5.48E+24)	1.16E+11 (24.65, 2.86E+24)	7.44E+10 (12.08, 1.93E+24)	2.14E+10 (4.76, 5.40E+24)	4.06E+10 (8.25, 1.07E+24)	4.06E+10 (8.11, 1.09E+24)	3.51E+10 (8.06, 9.43E+24)	2.67E+10 (5.89, 7.35E+24)	5.33E+10 (9.04, 1.27E+24)	4.64E+10 (8.80, 1.15E+24)	5.72E+10 (1.18, 1.38E+24)	4.35E+10 (9.85, 1.17E+24)	3.64E+10 (6.14, 9.29E+24)	2.53E+10 (5.62, 6.82E+24)	1.25E+10 (21.89, 2.52E+24)	9.84E+10 (20.18, 2.41E+24)	3.22E+10 (6.84, 9.63E+24)	TACE+RT	6.06E+10 (11.34, 1.42E+24)	4.49E+10 (6.73, 1.35E+24)	1.82E+10 (32.63, 4.77E+24)
0.32 (0.05, 1.97)	2.02 (0.56, 7.31)	1.19 (0.14, 9.95)	0.39 (0.11, 1.32)	0.65 (0.12, 3.60)	0.70 (0.10, 4.90)	0.65 (0.18, 2.33)	0.49 (0.15, 1.50)	0.83 (0.17, 3.93)	0.78 (0.14, 4.27)	0.10 (0.02, 0.39)	0.78 (0.25, 2.42)	0.54 (0.07, 4.14)	0.45 (0.14, 1.46)	2.04 (0.31, 14.01)	1.71 (0.26, 11.75)	0.57 (0.17, 1.98)	0.00 (0.00, 0.09)	TACE+SR	0.72 (0.06, 7.92)	2.91 (0.35, 25.48)
0.46 (0.04, 6.17)	2.87 (0.31, 28.82)	1.70 (0.10, 28.85)	0.54 (0.06, 5.21)	0.93 (0.08, 11.01)	0.99 (0.07, 14.94)	0.92 (0.10, 8.36)	0.68 (0.08, 5.94)	1.16 (0.11, 13.66)	1.11 (0.09, 13.93)	0.14 (0.01, 1.42)	1.10 (0.13, 9.66)	0.75 (0.05, 11.96)	0.63 (0.08, 5.49)	2.95 (0.21, 40.29)	2.42 (0.17, 32.18)	0.81 (0.10, 7.43)	0.00 (0.00, 0.15)	1.39 (0.13, 16.04)	TARE	4.24 (0.25, 67.64)
0.11 (0.01, 1.11)	0.69 (0.10, 4.86)	0.39 (0.03, 5.59)	0.13 (0.02, 0.89)	0.22 (0.06, 0.84)	0.24 (0.02, 2.80)	0.22 (0.03, 1.50)	0.16 (0.03, 1.01)	0.28 (0.03, 2.36)	0.26 (0.03, 2.41)	0.03 (0.00, 0.25)	0.26 (0.04, 1.66)	0.18 (0.02, 2.20)	0.15 (0.03, 0.93)	0.70 (0.06, 7.58)	0.57 (0.05, 6.40)	0.20 (0.03, 1.32)	0.00 (0.00, 0.03)	0.34 (0.04, 2.86)	0.24 (0.01, 4.00)	Tace+PAI

BCLC Stage A.5-year overall survival

HIFU+TACE	2.90 (0.49, 16.92)	5.92 (0.56, 65.08)	0.79 (0.13, 4.70)	1.14 (0.13, 8.97)	0.42 (0.04, 3.95)	0.68 (0.11, 3.72)	0.65 (0.12, 3.44)	1.06 (0.13, 8.26)	1.01 (0.12, 8.53)	0.14 (0.02, 0.90)	1.02 (0.19, 5.41)	0.85 (0.06, 9.85)	0.48 (0.09, 2.40)	2.79 (0.26, 30.64)	2.55 (0.24, 26.90)	0.80 (0.14, 4.60)	1.79 (0.25, 12.31)	2.68 (0.09, 182.27)
0.35 (0.06, 2.02)	LT	2.01 (0.39, 10.31)	0.27 (0.10, 0.67)	0.38 (0.08, 1.72)	0.14 (0.03, 0.73)	0.23 (0.10, 0.50)	0.22 (0.11, 0.42)	0.36 (0.09, 1.40)	0.35 (0.08, 1.54)	0.05 (0.02, 0.13)	0.35 (0.19, 0.64)	0.29 (0.03, 2.24)	0.17 (0.08, 0.33)	0.96 (0.16, 5.88)	0.87 (0.15, 5.14)	0.27 (0.12, 0.63)	0.61 (0.19, 1.92)	0.88 (0.04, 51.63)
0.17 (0.02, 1.80)	0.50 (0.10, 2.55)	LT+TACE	0.13 (0.02, 0.89)	0.19 (0.02, 1.73)	0.07 (0.01, 0.72)	0.11 (0.02, 0.71)	0.11 (0.02, 0.64)	0.18 (0.02, 1.53)	0.17 (0.02, 1.55)	0.02 (0.00, 0.16)	0.17 (0.03, 0.99)	0.14 (0.01, 1.86)	0.08 (0.01, 0.48)	0.48 (0.04, 5.42)	0.44 (0.04, 4.83)	0.14 (0.02, 0.84)	0.30 (0.04, 2.16)	0.44 (0.02, 31.85)
1.26 (0.21, 7.89)	3.66 (1.50, 9.59)	7.44 (1.12, 47.91)	MWA	1.42 (0.30, 6.60)	0.53 (0.10, 2.76)	0.84 (0.35, 2.07)	0.82 (0.40, 1.70)	1.32 (0.32, 5.46)	1.29 (0.28, 5.86)	0.18 (0.06, 0.53)	1.29 (0.65, 2.62)	1.06 (0.11, 8.94)	0.61 (0.28, 1.33)	3.53 (0.58, 22.64)	3.21 (0.52, 20.38)	1.01 (0.41, 2.51)	2.27 (0.68, 7.38)	3.34 (0.16, 189.41)
0.88 (0.11, 7.52)	2.62 (0.58, 12.15)	5.25 (0.58, 47.67)	0.70 (0.15, 3.36)	PAI	0.37 (0.05, 2.87)	0.60 (0.13, 2.66)	0.58 (0.14, 2.34)	0.94 (0.15, 6.05)	0.90 (0.13, 6.38)	0.13 (0.03, 0.62)	0.91 (0.23, 3.69)	0.75 (0.06, 8.44)	0.43 (0.11, 1.66)	2.51 (0.28, 22.78)	2.28 (0.26, 19.65)	0.72 (0.16, 3.09)	1.60 (0.29, 8.61)	2.37 (0.09, 141.08)
2.41 (0.25, 23.64)	6.95 (1.37, 36.09)	14.06 (1.38, 142.77)	1.89 (0.36, 9.83)	2.70 (0.35, 20.69)	PC	1.59 (0.32, 8.00)	1.54 (0.34, 7.15)	2.54 (0.35, 17.87)	2.43 (0.33, 19.03)	0.35 (0.06, 1.97)	2.45 (0.54, 11.02)	1.97 (0.14, 25.19)	1.15 (0.25, 5.43)	6.81 (0.70, 65.99)	6.18 (0.64, 58.78)	1.91 (0.37, 9.72)	4.30 (0.72, 25.89)	6.25 (0.25, 429.49)
1.48 (0.27, 8.97)	4.35 (1.98, 10.00)	8.80 (1.42, 54.55)	1.19 (0.48, 2.87)	1.66 (0.38, 7.66)	0.63 (0.13, 3.14)	PEI	0.97 (0.54, 1.75)	1.55 (0.41, 6.22)	1.54 (0.35, 6.46)	0.22 (0.08, 0.55)	1.53 (0.88, 2.69)	1.26 (0.14, 9.58)	0.72 (0.38, 1.39)	4.19 (0.74, 25.79)	3.78 (0.80, 18.69)	1.20 (0.53, 2.66)	2.68 (0.87, 8.21)	3.92 (0.20, 217.23)
1.54 (0.29, 8.37)	4.49 (2.38, 8.70)	9.11 (1.56, 51.51)	1.23 (0.59, 2.50)	1.74 (0.43, 7.14)	0.65 (0.14, 2.96)	1.03 (0.57, 1.84)	RFA	1.62 (0.48, 5.63)	1.59 (0.39, 6.31)	0.22 (0.10, 0.50)	1.58 (1.27, 1.98)	1.30 (0.15, 9.42)	0.74 (0.50, 1.12)	4.35 (0.81, 24.51)	3.93 (0.74, 20.71)	1.23 (0.69, 2.26)	2.76 (1.01, 7.43)	3.98 (0.22, 224.38)
0.95 (0.12, 7.54)	2.79 (0.71, 11.16)	5.61 (0.65, 47.58)	0.75 (0.18, 3.13)	1.07 (0.17, 6.89)	0.39 (0.06, 2.89)	0.64 (0.16, 2.46)	0.62 (0.18, 2.10)	RFA+PEI	0.98 (0.15, 6.05)	0.14 (0.03, 0.59)	0.98 (0.28, 3.41)	0.79 (0.07, 8.34)	0.46 (0.13, 1.66)	2.69 (0.34, 21.79)	2.42 (0.31, 18.69)	0.76 (0.20, 2.95)	1.72 (0.35, 7.99)	2.56 (0.10, 165.93)
0.99 (0.12, 8.35)	2.88 (0.65, 13.24)	5.74 (0.65, 55.56)	0.78 (0.17, 3.59)	1.11 (0.16, 7.89)	0.41 (0.05, 3.06)	0.65 (0.15, 2.88)	0.63 (0.16, 2.56)	1.02 (0.17, 6.48)	RFA+Sorafenib	0.14 (0.03, 0.69)	1.00 (0.26, 4.03)	0.84 (0.07, 8.94)	0.47 (0.12, 1.97)	2.80 (0.31, 24.06)	2.51 (0.29, 22.08)	0.78 (0.18, 3.52)	1.76 (0.33, 9.44)	2.62 (0.11, 168.68)
6.90 (1.12, 45.08)	20.24 (7.53, 55.21)	40.82 (6.25, 273.20)	5.50 (1.88, 16.17)	7.75 (1.60, 38.16)	2.89 (0.51, 16.02)	4.62 (1.82, 11.94)	4.47 (2.01, 10.39)	7.23 (1.69, 33.17)	7.06 (1.44, 33.85)	SC	7.05 (3.18, 16.13)	5.77 (0.58, 49.94)	3.35 (1.44, 7.96)	19.44 (3.14, 123.46)	17.81 (2.87, 111.73)	5.52 (2.10, 15.08)	12.38 (3.58, 44.03)	17.95 (0.86, 1.09E+3.13)
0.98 (0.18, 5.35)	2.84 (1.56, 5.38)	5.74 (1.01, 32.43)	0.77 (0.38, 1.55)	1.10 (0.27, 4.44)	0.41 (0.09, 1.84)	0.65 (0.37, 1.14)	0.63 (0.50, 0.79)	1.02 (0.29, 3.61)	1.00 (0.25, 3.78)	0.14 (0.06, 0.31)	SR	0.83 (0.09, 5.79)	0.47 (0.32, 0.69)	2.75 (0.51, 14.86)	2.47 (0.47, 13.12)	0.78 (0.44, 1.40)	1.76 (0.65, 4.65)	2.54 (0.14, 139.26)

1.18 (0.10, 17.99)	3.46 (0.45, 32.67)	7.07 (0.54, 107.28)	0.94 (0.11, 9.08)	1.33 (0.12, 17.42)	0.51 (0.04, 7.11)	0.80 (0.10, 7.17)	0.77 (0.11, 6.80)	1.26 (0.12, 14.87)	1.20 (0.11, 15.28)	0.17 (0.02, 1.73)	1.21 (0.17, 10.74)	SR+RFA	0.57 (0.08, 4.87)	3.39 (0.25, 48.53)	3.02 (0.23, 47.30)	0.94 (0.12, 8.66)	2.13 (0.23, 22.34)	3.26 (0.09, 287.19)
2.07 (0.42, 10.82)	6.02 (3.02, 12.40)	12.22 (2.07, 71.73)	1.64 (0.75, 3.63)	2.31 (0.60, 9.13)	0.87 (0.18, 4.08)	1.38 (0.72, 2.64)	1.34 (0.89, 2.01)	2.18 (0.60, 7.88)	2.12 (0.51, 8.61)	0.30 (0.13, 0.69)	2.12 (1.46, 3.16)	1.75 (0.21, 12.86)	TACE	5.84 (1.06, 33.80)	5.26 (0.97, 29.30)	1.65 (0.85, 3.22)	3.73 (1.29, 10.58)	5.36 (0.29, 292.67)
0.36 (0.03, 3.81)	1.04 (0.17, 6.40)	2.10 (0.18, 22.56)	0.28 (0.04, 1.72)	0.40 (0.04, 3.55)	0.15 (0.02, 1.43)	0.24 (0.04, 1.35)	0.23 (0.04, 1.24)	0.37 (0.05, 2.96)	0.36 (0.04, 3.26)	0.05 (0.01, 0.32)	0.36 (0.07, 1.97)	0.30 (0.02, 4.01)	0.17 (0.03, 0.94)	TACE +MWA	0.91 (0.08, 9.80)	0.28 (0.06, 1.38)	0.64 (0.09, 4.57)	0.95 (0.03, 69.43)
0.39 (0.04, 4.15)	1.15 (0.19, 6.79)	2.30 (0.21, 25.76)	0.31 (0.05, 1.93)	0.44 (0.05, 3.89)	0.16 (0.02, 1.57)	0.26 (0.05, 1.26)	0.25 (0.05, 1.35)	0.41 (0.05, 3.21)	0.40 (0.05, 3.40)	0.06 (0.01, 0.35)	0.40 (0.08, 2.15)	0.33 (0.02, 4.30)	0.19 (0.03, 1.03)	1.10 (0.10, 11.89)	TACE +PEI	0.31 (0.05, 1.74)	0.71 (0.10, 4.66)	1.04 (0.04, 73.87)
1.25 (0.22, 7.38)	3.65 (1.58, 8.68)	7.31 (1.18, 45.29)	0.99 (0.40, 2.41)	1.40 (0.32, 6.35)	0.52 (0.10, 2.71)	0.84 (0.38, 1.88)	0.81 (0.44, 1.46)	1.32 (0.34, 5.10)	1.28 (0.28, 5.70)	0.18 (0.07, 0.48)	1.28 (0.72, 2.30)	1.06 (0.12, 8.23)	0.61 (0.31, 1.18)	3.51 (0.72, 17.59)	3.20 (0.57, 18.20)	TACE+RFA	2.25 (0.71, 6.87)	3.27 (0.17, 194.35)
0.56 (0.08, 3.95)	1.63 (0.52, 5.28)	3.30 (0.46, 24.91)	0.44 (0.14, 1.46)	0.62 (0.12, 3.48)	0.23 (0.04, 1.38)	0.37 (0.12, 1.15)	0.36 (0.13, 0.99)	0.58 (0.13, 2.87)	0.57 (0.11, 3.07)	0.08 (0.02, 0.28)	0.57 (0.22, 1.53)	0.47 (0.04, 4.29)	0.27 (0.09, 0.78)	1.56 (0.22, 11.28)	1.42 (0.21, 9.90)	0.44 (0.15, 1.41)	TACE +SR	1.44 (0.07, 88.10)
0.37 (0.01, 11.13)	1.13 (0.02, 22.65)	2.25 (0.03, 64.31)	0.30 (0.01, 6.31)	0.42 (0.01, 10.63)	0.16 (0.00, 4.01)	0.26 (0.00, 4.92)	0.25 (0.00, 4.58)	0.39 (0.01, 9.88)	0.38 (0.01, 9.44)	0.06 (0.00, 1.16)	0.39 (0.01, 7.35)	0.31 (0.00, 10.85)	0.19 (0.00, 3.40)	1.05 (0.01, 31.83)	0.96 (0.01, 27.58)	0.31 (0.01, 5.92)	0.69 (0.01, 14.73)	TARE

BCLC Stage B. 1-year overall survival

RFA	0.19 (0.02, 2.07)	1.92 (0.22, 18.56)	1.69 (0.13, 22.49)	0.73 (0.06, 9.27)	1.06 (0.08, 16.32)	0.84 (0.09, 8.15)	2.98 (0.08, 95.25)	2.01 (0.09, 43.89)	4.06 (0.35, 49.25)	2.30 (0.19, 28.03)	12.00 (0.67, 249.99)	0.84 (0.07, 11.17)
5.20 (0.48, 53.59)	SC	10.04 (4.64, 23.55)	8.73 (1.82, 42.03)	3.80 (1.02, 14.72)	5.52 (1.03, 30.98)	4.39 (2.03, 10.07)	14.83 (0.83, 256.18)	10.34 (1.15, 95.86)	20.99 (5.72, 81.67)	11.86 (3.06, 46.36)	60.73 (8.00, 547.19)	4.43 (1.06, 20.12)
0.52 (0.05, 4.49)	0.10 (0.04, 0.22)	SR	0.87 (0.21, 3.39)	0.37 (0.11, 1.27)	0.54 (0.11, 2.53)	0.43 (0.28, 0.65)	1.46 (0.09, 22.51)	1.03 (0.12, 8.57)	2.06 (0.67, 6.66)	1.18 (0.35, 3.81)	5.99 (0.88, 45.87)	0.44 (0.12, 1.59)
0.59 (0.04, 7.73)	0.11 (0.02, 0.55)	1.16 (0.29, 4.72)	SR+RFA	0.43 (0.07, 2.64)	0.64 (0.08, 4.57)	0.50 (0.13, 1.99)	1.68 (0.08, 34.40)	1.19 (0.09, 15.26)	2.40 (0.41, 14.11)	1.37 (0.23, 8.07)	6.98 (0.68, 81.25)	0.51 (0.08, 3.29)
1.36 (0.11, 16.85)	0.26 (0.07, 0.98)	2.68 (0.78, 9.20)	2.30 (0.38, 13.49)	Sorafenib	1.44 (0.22, 10.22)	1.16 (0.36, 3.76)	3.85 (0.20, 73.17)	2.73 (0.24, 27.60)	5.49 (1.15, 27.45)	3.11 (0.61, 15.76)	16.19 (1.68, 185.82)	1.17 (0.20, 6.35)
0.94 (0.06, 13.07)	0.18 (0.03, 0.97)	1.84 (0.40, 8.78)	1.57 (0.22, 11.95)	0.69 (0.10, 4.59)	TAC	0.80 (0.17, 3.52)	2.69 (0.12, 60.62)	1.89 (0.14, 23.89)	3.88 (0.61, 23.16)	2.15 (0.33, 13.65)	11.25 (0.92, 133.71)	0.81 (0.11, 5.58)
1.18 (0.12, 10.75)	0.23 (0.10, 0.49)	2.31 (1.53, 3.53)	1.99 (0.50, 7.77)	0.86 (0.27, 2.81)	1.25 (0.28, 5.73)	TACE	3.41 (0.22, 51.37)	2.37 (0.28, 18.92)	4.79 (1.68, 14.21)	2.70 (0.87, 8.23)	13.83 (2.00, 110.70)	1.01 (0.29, 3.44)
0.34 (0.01, 11.87)	0.07 (0.00, 1.20)	0.68 (0.04, 11.01)	0.59 (0.03, 12.92)	0.26 (0.01, 4.98)	0.37 (0.02, 8.12)	0.29 (0.02, 4.49)	TACE +MWA	0.70 (0.02, 20.86)	1.44 (0.11, 18.05)	0.80 (0.04, 16.00)	4.11 (0.14, 131.41)	0.30 (0.01, 5.63)
0.50 (0.02, 11.36)	0.10 (0.01, 0.87)	0.97 (0.12, 8.52)	0.84 (0.07, 10.75)	0.37 (0.04, 4.14)	0.53 (0.04, 7.09)	0.42 (0.05, 3.59)	1.42 (0.05, 43.80)	TACE+PEI	2.00 (0.20, 22.54)	1.14 (0.11, 12.31)	5.83 (0.35, 116.49)	0.43 (0.04, 4.82)
0.25 (0.02, 2.84)	0.05 (0.01, 0.17)	0.49 (0.15, 1.49)	0.42 (0.07, 2.42)	0.18 (0.04, 0.87)	0.26 (0.04, 1.64)	0.21 (0.07, 0.60)	0.70 (0.06, 8.74)	0.50 (0.04, 4.95)	TACE+RFA	0.56 (0.12, 2.66)	2.89 (0.30, 29.88)	0.21 (0.04, 1.08)

0.44 (0.04, 5.25)	0.08 (0.02, 0.33)	0.85 (0.26, 2.83)	0.73 (0.12, 4.31)	0.32 (0.06, 1.65)	0.47 (0.07, 3.01)	0.37 (0.12, 1.15)	1.26 (0.06, 23.30)	0.88 (0.08, 9.05)	1.78 (0.38, 8.60)	TACE+RT	5.20 (0.55, 54.27)	0.38 (0.07, 1.97)
0.08 (0.00, 1.49)	0.02 (0.00, 0.13)	0.17 (0.02, 1.14)	0.14 (0.01, 1.48)	0.06 (0.01, 0.60)	0.09 (0.01, 1.08)	0.07 (0.01, 0.50)	0.24 (0.01, 6.97)	0.17 (0.01, 2.83)	0.35 (0.03, 3.34)	0.19 (0.02, 1.82)	TACE+SR	0.07 (0.01, 0.70)
1.19 (0.09, 13.93)	0.23 (0.05, 0.95)	2.29 (0.63, 8.32)	1.97 (0.30, 12.13)	0.86 (0.16, 4.91)	1.24 (0.18, 8.86)	0.99 (0.29, 3.41)	3.39 (0.18, 66.75)	2.31 (0.21, 26.19)	4.72 (0.93, 24.78)	2.66 (0.51, 14.17)	13.62 (1.42, 156.44)	TARE

BCLC Stage B. 3-year overall survival

RFA	1.01 (0.06, 20.38)	11.37 (0.92, 190.74)	7.94 (0.37, 228.14)	4.96 (0.21, 139.33)	3.95 (0.16, 114.40)	3.69 (0.30, 65.28)	14.82 (0.08, 3.10E+3)	2.22 (0.02, 150.52)	17.80 (1.13, 401.19)	4.80 (0.16, 160.25)	137.79 (4.64, 5.33E+3)	16.16 (0.74, 430.81)
0.99 (0.05, 16.63)	SC	11.30 (3.81, 39.86)	8.06 (1.03, 65.55)	4.86 (0.68, 38.00)	3.81 (0.44, 39.25)	3.75 (1.22, 12.96)	13.97 (0.15, 1.53E+3)	2.26 (0.05, 67.15)	17.68 (3.75, 104.25)	4.64 (0.41, 53.76)	136.63 (11.54, 1.91E+3)	15.67 (2.17, 144.25)
0.09 (0.01, 1.08)	0.09 (0.03, 0.26)	SR	0.70 (0.12, 3.89)	0.44 (0.06, 2.62)	0.34 (0.05, 2.37)	0.33 (0.19, 0.55)	1.22 (0.01, 114.19)	0.19 (0.00, 5.12)	1.57 (0.43, 5.81)	0.42 (0.04, 3.49)	11.91 (1.21, 116.48)	1.38 (0.24, 9.00)
0.13 (0.00, 2.70)	0.12 (0.02, 0.97)	1.43 (0.26, 8.12)	SR+RFA	0.62 (0.05, 7.01)	0.48 (0.04, 6.40)	0.47 (0.09, 2.63)	1.76 (0.01, 229.96)	0.28 (0.00, 10.55)	2.25 (0.27, 18.92)	0.60 (0.04, 8.90)	16.89 (1.03, 319.57)	1.98 (0.17, 25.89)
0.20 (0.01, 4.76)	0.21 (0.03, 1.47)	2.29 (0.38, 15.56)	1.62 (0.14, 19.48)	Sorafenib	0.79 (0.06, 10.41)	0.76 (0.13, 4.71)	2.91 (0.03, 389.68)	0.45 (0.01, 18.23)	3.60 (0.48, 32.88)	0.96 (0.06, 15.10)	27.61 (1.52, 566.47)	3.19 (0.28, 40.18)
0.25 (0.01, 6.08)	0.26 (0.03, 2.28)	2.96 (0.42, 21.70)	2.08 (0.16, 26.53)	1.27 (0.10, 17.07)	TAC	0.97 (0.14, 6.38)	3.62 (0.03, 490.31)	0.58 (0.01, 24.66)	4.58 (0.51, 44.81)	1.21 (0.07, 21.14)	35.41 (1.90, 716.30)	4.10 (0.32, 56.55)
0.27 (0.02, 3.37)	0.27 (0.08, 0.82)	3.03 (1.82, 5.27)	2.13 (0.38, 11.73)	1.32 (0.21, 7.41)	1.03 (0.16, 6.90)	TACE	3.74 (0.05, 350.41)	0.59 (0.01, 14.83)	4.72 (1.51, 15.56)	1.26 (0.15, 10.12)	36.00 (3.58, 375.58)	4.18 (0.81, 25.29)
0.07 (0.00, 11.82)	0.07 (0.00, 6.89)	0.82 (0.01, 70.44)	0.57 (0.00, 68.94)	0.34 (0.00, 39.63)	0.28 (0.00, 32.01)	0.27 (0.00, 22.05)	TACE+MWA	0.15 (0.00, 39.77)	1.30 (0.01, 89.93)	0.33 (0.00, 44.37)	9.82 (0.06, 1302.60)	1.15 (0.01, 130.23)
0.45 (0.01, 40.93)	0.44 (0.01, 21.09)	5.17 (0.20, 228.91)	3.57 (0.09, 213.13)	2.23 (0.05, 149.71)	1.73 (0.04, 111.70)	1.69 (0.07, 72.30)	6.83 (0.03, 2070.72)	TACE+PEI	8.16 (0.25, 397.46)	2.14 (0.05, 144.37)	62.40 (1.17, 5112.59)	7.36 (0.18, 445.26)
0.06 (0.00, 0.88)	0.06 (0.01, 0.27)	0.64 (0.17, 2.32)	0.44 (0.05, 3.67)	0.28 (0.03, 2.09)	0.22 (0.02, 1.98)	0.21 (0.06, 0.66)	0.77 (0.01, 66.81)	0.12 (0.00, 4.01)	TACE+RFA	0.27 (0.02, 2.86)	7.59 (0.57, 100.17)	0.90 (0.11, 7.55)
0.21 (0.01, 6.09)	0.22 (0.02, 2.41)	2.40 (0.29, 22.91)	1.68 (0.11, 27.40)	1.05 (0.07, 15.93)	0.83 (0.05, 14.53)	0.79 (0.10, 6.84)	3.05 (0.02, 394.79)	0.47 (0.01, 20.44)	3.76 (0.35, 48.65)	TACE+RT	29.01 (1.32, 696.97)	3.39 (0.23, 56.30)
0.01 (0.00, 0.22)	0.01 (0.00, 0.09)	0.08 (0.01, 0.82)	0.06 (0.00, 0.97)	0.04 (0.00, 0.66)	0.03 (0.00, 0.53)	0.03 (0.00, 0.28)	0.10 (0.00, 15.49)	0.02 (0.00, 0.86)	0.13 (0.01, 1.76)	0.03 (0.00, 0.76)	TACE+SR	0.12 (0.01, 2.37)
0.06 (0.00, 1.35)	0.06 (0.01, 0.46)	0.72 (0.11, 4.11)	0.50 (0.04, 5.85)	0.31 (0.02, 3.58)	0.24 (0.02, 3.08)	0.24 (0.04, 1.23)	0.87 (0.01, 117.41)	0.14 (0.00, 5.59)	1.11 (0.13, 9.51)	0.29 (0.02, 4.36)	8.59 (0.42, 152.85)	TARE

BCLC Stage C. 1-year overall survival

HAC	2.10 (0.21, 22.44)	3.55 (0.99, 13.72)	2.11 (0.35, 12.92)	0.83 (0.06, 11.57)	0.90 (0.04, 26.98)	0.05 (0.01, 0.17)	0.33 (0.04, 2.49)	0.70 (0.19, 2.52)	0.40 (0.05, 3.72)	2.04 (0.29, 14.87)	0.90 (0.09, 8.44)	3.19 (0.43, 23.60)	2.14 (0.19, 24.22)	2.69 (0.19, 40.11)	0.95 (0.22, 3.97)	2.30 (0.33, 16.19)	0.21 (0.06, 0.74)	0.38 (0.11, 1.35)	1.90 (0.15, 23.55)	0.41 (0.01, 27.93)	0.50 (0.03, 7.97)	0.26 (0.03, 2.01)	0.56 (0.04, 6.97)	1.20 (0.08, 15.86)	1.18 (0.27, 4.99)	3.87 (0.84, 16.86)	1.25 (0.29, 5.28)	1.76 (0.20, 16.87)
0.48 (0.04, 4.79)	HAC+RFA	1.68 (0.11, 25.36)	1.00 (0.05, 19.31)	0.40 (0.01, 13.23)	0.42 (0.01, 24.37)	0.02 (0.00, 0.33)	0.16 (0.01, 3.38)	0.33 (0.02, 4.72)	0.19 (0.01, 4.83)	0.94 (0.05, 19.13)	0.42 (0.02, 9.90)	1.54 (0.07, 33.56)	0.99 (0.04, 27.03)	1.25 (0.04, 48.90)	0.45 (0.03, 6.90)	1.05 (0.05, 23.31)	0.10 (0.01, 1.39)	0.18 (0.01, 2.49)	0.91 (0.03, 26.86)	0.19 (0.00, 26.08)	0.23 (0.01, 8.29)	0.12 (0.01, 2.72)	0.26 (0.01, 7.69)	0.57 (0.02, 18.36)	0.56 (0.03, 8.75)	1.79 (0.11, 28.73)	0.59 (0.04, 9.13)	0.84 (0.03, 21.66)
0.28 (0.07, 1.01)	0.60 (0.04, 8.88)	HAC+RT	0.59 (0.06, 5.45)	0.24 (0.01, 4.32)	0.25 (0.01, 9.00)	0.01 (0.00, 0.08)	0.09 (0.01, 1.04)	0.20 (0.03, 1.15)	0.11 (0.01, 1.44)	0.57 (0.05, 6.03)	0.25 (0.02, 3.35)	0.90 (0.08, 9.41)	0.60 (0.04, 9.40)	0.77 (0.04, 14.91)	0.27 (0.04, 1.83)	0.64 (0.06, 6.40)	0.06 (0.01, 0.35)	0.11 (0.02, 0.62)	0.54 (0.03, 8.67)	0.12 (0.00, 9.36)	0.14 (0.01, 2.88)	0.07 (0.01, 0.78)	0.16 (0.01, 2.59)	0.34 (0.02, 6.00)	0.33 (0.04, 2.30)	1.08 (0.14, 7.65)	0.36 (0.05, 2.45)	0.50 (0.04, 6.76)
0.47 (0.08, 2.88)	1.00 (0.05, 19.63)	1.69 (0.18, 16.01)	HAC+Sorafenib	0.40 (0.02, 7.24)	0.42 (0.01, 15.83)	0.02 (0.00, 0.15)	0.16 (0.01, 1.96)	0.33 (0.05, 2.14)	0.19 (0.02, 2.42)	0.96 (0.09, 10.32)	0.43 (0.03, 5.37)	1.50 (0.13, 17.62)	1.02 (0.06, 16.70)	1.26 (0.07, 27.81)	0.46 (0.06, 3.23)	1.08 (0.11, 11.48)	0.10 (0.02, 0.65)	0.18 (0.03, 1.06)	0.92 (0.05, 14.61)	0.20 (0.00, 16.08)	0.23 (0.01, 5.08)	0.12 (0.01, 1.37)	0.27 (0.01, 4.52)	0.57 (0.03, 10.76)	0.56 (0.08, 3.93)	1.82 (0.24, 12.84)	0.59 (0.08, 4.11)	0.85 (0.06, 11.14)
1.21 (0.09, 16.90)	2.49 (0.08, 87.28)	4.24 (0.23, 82.96)	2.49 (0.14, 49.77)	HIFU	1.05 (0.03, 54.58)	0.06 (0.01, 0.67)	0.40 (0.02, 8.71)	0.84 (0.08, 8.97)	0.48 (0.02, 9.87)	2.46 (0.15, 39.29)	1.06 (0.06, 20.76)	3.79 (0.21, 69.53)	2.52 (0.10, 65.45)	3.21 (0.11, 92.70)	1.14 (0.09, 13.19)	2.70 (0.18, 43.22)	0.25 (0.02, 2.95)	0.46 (0.04, 4.65)	2.28 (0.09, 55.46)	0.49 (0.00, 49.20)	0.60 (0.02, 17.30)	0.31 (0.02, 5.62)	0.67 (0.03, 16.26)	1.41 (0.05, 37.37)	1.41 (0.11, 17.81)	4.54 (0.36, 57.48)	1.49 (0.12, 17.97)	2.17 (0.10, 41.18)
1.11 (0.04, 27.67)	2.35 (0.04, 124.29)	3.96 (0.11, 130.60)	2.39 (0.06, 75.96)	0.95 (0.02, 39.18)	RT	0.06 (0.00, 1.18)	0.36 (0.01, 12.50)	0.79 (0.03, 16.38)	0.45 (0.01, 14.56)	2.25 (0.07, 61.24)	0.99 (0.03, 32.46)	3.59 (0.10, 105.96)	2.34 (0.05, 96.50)	3.04 (0.06, 139.08)	1.08 (0.04, 23.46)	2.57 (0.07, 70.60)	0.24 (0.01, 5.10)	0.43 (0.02, 8.40)	2.15 (0.04, 82.22)	0.46 (0.00, 68.91)	0.57 (0.01, 27.09)	0.29 (0.01, 8.60)	0.62 (0.01, 23.02)	1.30 (0.03, 59.15)	1.29 (0.05, 28.53)	4.30 (0.16, 92.81)	1.41 (0.05, 30.71)	2.03 (0.05, 69.54)
19.26 (5.76, 71.68)	41.15 (3.03, 609.77)	68.19 (12.17, 465.37)	40.61 (6.47, 280.63)	16.23 (1.49, 186.31)	17.23 (0.85, 438.86)	SC	6.44 (0.94, 48.07)	13.59 (6.83, 28.54)	7.92 (1.17, 52.37)	39.60 (7.87, 207.28)	17.55 (2.45, 129.29)	62.35 (11.12, 351.33)	40.46 (4.64, 437.33)	52.14 (4.92, 630.75)	18.45 (7.17, 50.19)	43.61 (9.22, 236.82)	4.10 (1.80, 10.15)	7.43 (3.94, 14.93)	36.64 (3.75, 374.69)	7.87 (0.13, 495.03)	9.78 (0.77, 124.23)	5.06 (0.97, 28.56)	10.85 (1.14, 109.56)	22.93 (2.00, 273.85)	22.57 (8.17, 67.11)	74.18 (25.99, 228.17)	24.08 (8.81, 69.84)	34.55 (4.93, 254.12)
3.00 (0.40, 23.13)	6.33 (0.30, 148.94)	10.62 (0.96, 120.94)	6.35 (0.51, 83.65)	2.50 (0.11, 52.04)	2.75 (0.08, 106.91)	0.16 (0.02, 1.06)	SCT	2.12 (0.30, 13.84)	1.23 (0.09, 17.02)	6.09 (0.53, 64.85)	2.68 (0.19, 39.19)	9.88 (0.78, 108.38)	6.36 (0.77, 55.69)	8.27 (0.39, 163.49)	2.86 (0.35, 22.46)	6.83 (0.62, 83.88)	0.64 (0.09, 4.72)	1.16 (0.16, 7.96)	5.77 (0.29, 100.41)	1.21 (0.01, 109.83)	1.49 (0.06, 34.36)	0.79 (0.07, 8.98)	1.69 (0.09, 30.39)	3.64 (0.16, 73.63)	3.54 (0.44, 28.64)	11.55 (1.42, 94.35)	3.76 (0.45, 30.24)	5.35 (0.37, 85.04)
1.42 (0.40, 5.28)	3.03 (0.21, 44.01)	4.94 (0.87, 34.67)	2.99 (0.47, 19.23)	1.19 (0.11, 13.29)	1.27 (0.06, 33.12)	0.07 (0.04, 0.15)	0.47 (0.07, 3.33)	SR	0.58 (0.09, 3.46)	2.88 (0.67, 13.07)	1.27 (0.19, 8.45)	4.56 (0.93, 22.96)	2.94 (0.35, 29.64)	3.80 (0.38, 41.39)	1.36 (0.58, 3.08)	3.20 (0.74, 14.94)	0.30 (0.13, 0.72)	0.54 (0.31, 0.97)	2.68 (0.27, 26.16)	0.58 (0.01, 37.39)	0.72 (0.05, 8.76)	0.37 (0.08, 1.85)	0.80 (0.08, 7.54)	1.68 (0.15, 18.74)	1.66 (0.66, 4.37)	5.42 (1.93, 15.78)	1.77 (0.65, 4.86)	2.52 (0.36, 18.08)
2.47 (0.27, 21.61)	5.27 (0.21, 130.51)	8.79 (0.69, 120.48)	5.19 (0.41, 66.21)	2.07 (0.10, 41.10)	2.21 (0.07, 82.27)	0.13 (0.02, 0.86)	0.82 (0.06, 11.67)	1.71 (0.29, 10.59)	SR+HAC	4.90 (0.50, 50.72)	2.19 (0.17, 28.76)	7.86 (0.76, 86.22)	5.20 (0.32, 97.56)	6.65 (0.38, 129.49)	2.32 (0.33, 17.57)	5.59 (0.57, 57.36)	0.52 (0.07, 3.95)	0.93 (0.15, 6.08)	4.59 (0.26, 87.80)	1.01 (0.01, 97.42)	1.23 (0.05, 26.12)	0.64 (0.06, 7.02)	1.37 (0.08, 24.52)	2.86 (0.15, 57.20)	2.87 (0.40, 22.18)	9.26 (1.23, 78.75)	3.04 (0.42, 24.11)	4.43 (0.31, 63.83)
0.49 (0.07, 3.44)	1.06 (0.05, 21.50)	1.76 (0.17, 18.61)	1.04 (0.10, 10.87)	0.41 (0.03, 6.62)	0.45 (0.02, 13.65)	0.03 (0.00, 0.13)	0.16 (0.02, 1.88)	0.35 (0.08, 1.50)	0.20 (0.02, 2.01)	SR+PVC	0.44 (0.04, 4.68)	1.58 (0.17, 14.42)	1.04 (0.08, 14.57)	1.34 (0.08, 20.91)	0.47 (0.09, 2.46)	1.12 (0.14, 9.17)	0.11 (0.02, 0.57)	0.19 (0.04, 0.87)	0.94 (0.06, 13.68)	0.20 (0.00, 16.05)	0.25 (0.01, 4.48)	0.13 (0.01, 1.14)	0.27 (0.02, 3.91)	0.59 (0.03, 9.48)	0.57 (0.10, 3.34)	1.87 (0.30, 11.15)	0.62 (0.11, 3.58)	0.88 (0.07, 10.34)
1.11 (0.12, 10.68)	2.37 (0.10, 60.50)	4.00 (0.30, 54.23)	2.34 (0.19, 31.25)	0.94 (0.05, 17.96)	1.01 (0.03, 39.67)	0.06 (0.01, 0.41)	0.37 (0.03, 5.25)	0.79 (0.12, 5.21)	0.46 (0.03, 5.96)	2.28 (0.21, 24.93)	SR+RFA	3.60 (0.31, 43.46)	2.35 (0.14, 45.46)	3.00 (0.15, 64.82)	1.06 (0.14, 8.07)	2.56 (0.24, 29.04)	0.24 (0.03, 1.78)	0.43 (0.06, 2.80)	2.13 (0.11, 39.40)	0.46 (0.01, 41.84)	0.56 (0.02, 12.39)	0.29 (0.02, 3.45)	0.62 (0.04, 10.79)	1.33 (0.07, 26.93)	1.30 (0.17, 10.69)	4.24 (0.54, 34.65)	1.38 (0.17, 10.67)	1.98 (0.14, 29.02)
0.31 (0.04, 2.35)	0.65 (0.03, 14.36)	1.12 (0.11, 12.69)	0.67 (0.06, 7.61)	0.26 (0.01, 4.84)	0.28 (0.01, 9.65)	0.02 (0.00, 0.09)	0.10 (0.01, 1.28)	0.22 (0.04, 1.07)	0.13 (0.01, 1.32)	0.63 (0.07, 5.78)	0.28 (0.02, 3.21)	SR+RT	0.65 (0.05, 10.01)	0.84 (0.05, 14.49)	0.30 (0.05, 1.59)	0.70 (0.08, 6.43)	0.07 (0.01, 0.40)	0.12 (0.02, 0.62)	0.59 (0.03, 9.54)	0.13 (0.00, 10.31)	0.15 (0.01, 2.88)	0.08 (0.01, 0.78)	0.17 (0.01, 2.77)	0.37 (0.02, 6.70)	0.37 (0.06, 2.32)	1.19 (0.18, 7.96)	0.39 (0.06, 2.53)	0.56 (0.04, 7.11)

0.47 (0.04, 5.17)	1.01 (0.04, 27.24)	1.67 (0.11, 27.68)	0.98 (0.06, 15.77)	0.40 (0.02, 9.56)	0.43 (0.01, 20.02)	0.02 (0.00, 0.22)	0.16 (0.02, 1.29)	0.34 (0.03, 2.86)	0.19 (0.01, 3.13)	0.96 (0.07, 12.85)	0.43 (0.02, 7.15)	1.54 (0.10, 21.63)	SR+SCT	1.28 (0.05, 29.79)	0.45 (0.04, 4.44)	1.09 (0.07, 14.84)	0.10 (0.01, 1.01)	0.18 (0.02, 1.58)	0.89 (0.04, 20.51)	0.19 (0.00, 21.40)	0.23 (0.01, 6.53)	0.12 (0.01, 1.75)	0.26 (0.01, 5.80)	0.56 (0.02, 13.70)	0.56 (0.05, 5.69)	1.81 (0.16, 19.48)	0.59 (0.05, 6.36)	0.84 (0.05, 14.87)
0.37 (0.02, 5.29)	0.80 (0.02, 25.36)	1.31 (0.07, 26.63)	0.79 (0.04, 15.20)	0.31 (0.01, 9.06)	0.33 (0.01, 15.82)	0.02 (0.00, 0.20)	0.12 (0.01, 2.54)	0.26 (0.02, 2.64)	0.15 (0.01, 2.64)	0.75 (0.05, 12.77)	0.33 (0.02, 6.65)	1.19 (0.07, 20.37)	0.78 (0.03, 20.05)	SR+Sorafenib	0.35 (0.03, 4.05)	0.84 (0.05, 12.96)	0.08 (0.01, 0.96)	0.14 (0.01, 1.56)	0.71 (0.03, 17.70)	0.15 (0.00, 17.14)	0.19 (0.01, 5.77)	0.10 (0.01, 1.73)	0.21 (0.01, 5.64)	0.43 (0.02, 11.64)	0.44 (0.03, 5.49)	1.42 (0.11, 17.55)	0.46 (0.04, 5.95)	0.66 (0.03, 14.98)
1.05 (0.25, 4.50)	2.21 (0.15, 34.11)	3.72 (0.55, 27.85)	2.20 (0.31, 16.28)	0.88 (0.08, 11.13)	0.92 (0.04, 24.84)	0.05 (0.02, 0.14)	0.35 (0.04, 2.82)	0.74 (0.32, 1.72)	0.43 (0.06, 3.00)	2.12 (0.41, 11.75)	0.94 (0.12, 6.99)	3.36 (0.63, 18.42)	2.21 (0.23, 24.18)	2.84 (0.25, 35.27)	SR+TACE	2.36 (0.44, 14.07)	0.23 (0.08, 0.64)	0.40 (0.17, 0.97)	2.00 (0.18, 20.93)	0.42 (0.01, 28.91)	0.53 (0.04, 7.17)	0.27 (0.05, 1.69)	0.58 (0.06, 6.29)	1.23 (0.10, 15.19)	1.23 (0.42, 3.66)	3.99 (1.24, 13.43)	1.31 (0.40, 4.34)	1.85 (0.24, 14.58)
0.44 (0.06, 3.01)	0.95 (0.04, 18.71)	1.57 (0.16, 16.04)	0.93 (0.09, 9.38)	0.37 (0.02, 5.70)	0.39 (0.01, 13.35)	0.02 (0.00, 0.11)	0.15 (0.01, 1.60)	0.31 (0.07, 1.36)	0.18 (0.02, 1.75)	0.89 (0.11, 7.13)	0.39 (0.03, 4.23)	1.43 (0.16, 12.46)	0.92 (0.07, 14.14)	1.19 (0.08, 19.67)	0.42 (0.07, 2.26)	SR+TACE+PVC	0.10 (0.02, 0.50)	0.17 (0.03, 0.80)	0.83 (0.05, 12.86)	0.18 (0.00, 14.71)	0.22 (0.01, 3.94)	0.11 (0.02, 0.61)	0.25 (0.02, 3.64)	0.52 (0.03, 8.73)	0.52 (0.09, 2.93)	1.69 (0.28, 9.95)	0.55 (0.09, 3.22)	0.79 (0.06, 8.79)
4.67 (1.35, 17.07)	9.98 (0.72, 140.80)	16.33 (2.89, 107.41)	9.78 (1.54, 65.31)	3.94 (0.34, 46.42)	4.15 (0.20, 107.15)	0.24 (0.10, 0.56)	1.55 (0.21, 11.58)	3.30 (1.40, 7.74)	1.92 (0.25, 13.38)	9.49 (1.75, 51.10)	4.19 (0.56, 32.14)	15.11 (2.49, 89.57)	9.82 (0.99, 107.83)	12.59 (1.04, 160.54)	4.44 (1.55, 13.09)	10.46 (1.99, 59.60)	Sorafenib	1.79 (0.84, 3.80)	8.88 (0.83, 89.36)	1.95 (0.03, 130.42)	2.33 (0.18, 30.41)	1.23 (0.21, 7.35)	2.62 (0.25, 25.90)	5.51 (0.48, 68.24)	5.49 (1.93, 15.73)	17.93 (6.53, 49.12)	5.84 (2.34, 14.37)	8.26 (1.35, 52.54)
2.60 (0.74, 9.50)	5.52 (0.40, 79.88)	9.10 (1.60, 60.46)	5.50 (0.94, 34.77)	2.17 (0.21, 23.18)	2.31 (0.12, 54.50)	0.13 (0.07, 0.25)	0.86 (0.13, 6.42)	1.84 (1.04, 3.25)	1.08 (0.16, 6.75)	5.36 (1.15, 25.13)	2.34 (0.36, 15.43)	8.33 (1.62, 43.89)	5.43 (0.63, 53.32)	7.01 (0.64, 79.59)	2.50 (1.03, 5.83)	5.88 (1.25, 29.41)	0.56 (0.26, 1.20)	TACE	4.93 (0.53, 42.79)	1.06 (0.02, 64.49)	1.32 (0.11, 14.74)	0.68 (0.13, 3.74)	1.46 (0.17, 13.01)	3.07 (0.30, 32.54)	3.05 (1.29, 7.51)	9.94 (3.95, 26.06)	3.24 (1.37, 7.68)	4.63 (0.72, 31.39)
0.53 (0.04, 6.66)	1.10 (0.04, 38.80)	1.86 (0.12, 32.81)	1.09 (0.07, 20.44)	0.44 (0.02, 11.25)	0.47 (0.01, 24.75)	0.03 (0.00, 0.27)	0.17 (0.01, 3.42)	0.37 (0.04, 3.69)	0.22 (0.01, 3.83)	1.06 (0.07, 16.22)	0.47 (0.03, 9.26)	1.69 (0.10, 28.82)	1.12 (0.05, 25.92)	1.42 (0.06, 37.67)	0.50 (0.05, 5.42)	1.20 (0.08, 19.53)	0.11 (0.01, 1.20)	0.20 (0.02, 1.88)	TACE+HAC	0.21 (0.00, 21.77)	0.26 (0.01, 7.16)	0.14 (0.01, 2.37)	0.29 (0.01, 6.33)	0.62 (0.03, 15.62)	0.62 (0.06, 6.88)	2.01 (0.19, 23.89)	0.65 (0.06, 7.24)	0.94 (0.05, 17.51)
2.43 (0.04, 161.06)	5.36 (0.04, 601.75)	8.34 (0.11, 731.90)	5.02 (0.06, 419.85)	2.02 (0.02, 216.30)	2.18 (0.01, 344.70)	0.13 (0.00, 7.50)	0.83 (0.01, 76.65)	1.73 (0.03, 104.93)	0.99 (0.01, 90.27)	4.91 (0.06, 394.87)	2.18 (0.02, 199.83)	7.79 (0.10, 659.92)	5.31 (0.05, 529.18)	6.53 (0.06, 730.22)	2.36 (0.03, 145.88)	5.62 (0.07, 432.30)	0.51 (0.01, 31.10)	0.95 (0.02, 54.59)	4.72 (0.05, 470.80)	TACE+HIFU	1.22 (0.01, 149.42)	0.64 (0.01, 53.78)	1.39 (0.01, 138.98)	2.96 (0.03, 282.83)	2.92 (0.04, 184.26)	9.04 (0.14, 609.26)	3.02 (0.04, 197.90)	4.43 (0.05, 381.99)
2.00 (0.13, 32.16)	4.41 (0.12, 162.28)	7.06 (0.35, 143.79)	4.27 (0.20, 90.76)	1.67 (0.06, 49.11)	1.76 (0.04, 107.64)	0.10 (0.01, 1.30)	0.67 (0.03, 16.82)	1.40 (0.11, 18.55)	0.81 (0.04, 18.25)	4.01 (0.22, 79.64)	1.80 (0.08, 43.96)	6.48 (0.35, 127.19)	4.27 (0.15, 126.98)	5.36 (0.17, 162.84)	1.90 (0.14, 25.80)	4.55 (0.25, 93.71)	0.43 (0.03, 5.62)	0.76 (0.07, 9.37)	3.86 (0.14, 106.93)	0.82 (0.01, 101.73)	TACE+PEI	0.53 (0.03, 10.89)	1.11 (0.04, 29.10)	2.35 (0.08, 70.21)	2.34 (0.18, 32.77)	7.73 (0.57, 108.67)	2.47 (0.18, 34.84)	3.60 (0.16, 80.03)
3.80 (0.50, 28.71)	8.12 (0.37, 174.38)	13.58 (1.29, 159.94)	8.16 (0.73, 86.28)	3.21 (0.18, 51.58)	3.42 (0.12, 109.40)	0.20 (0.04, 1.03)	1.27 (0.11, 15.26)	2.69 (0.54, 13.10)	1.55 (0.14, 17.16)	7.71 (0.88, 71.44)	3.41 (0.29, 40.27)	12.24 (1.29, 116.45)	8.08 (0.57, 128.00)	10.17 (0.58, 181.31)	3.69 (0.59, 20.69)	8.70 (1.64, 46.03)	0.81 (0.14, 4.74)	1.47 (0.27, 7.69)	7.32 (0.42, 114.36)	1.56 (0.02, 131.10)	1.90 (0.09, 36.96)	TACE+PVC	2.10 (0.13, 32.42)	4.51 (0.24, 80.60)	4.49 (0.73, 27.75)	14.49 (2.24, 95.28)	4.74 (0.75, 31.12)	6.85 (0.57, 79.09)
1.80 (0.14, 23.08)	3.85 (0.13, 128.18)	6.35 (0.39, 119.73)	3.71 (0.22, 67.20)	1.49 (0.06, 37.48)	1.62 (0.04, 79.63)	0.09 (0.01, 0.87)	0.59 (0.03, 11.43)	1.25 (0.13, 11.81)	0.73 (0.04, 12.66)	3.67 (0.26, 54.46)	1.62 (0.09, 28.28)	5.77 (0.36, 89.93)	3.81 (0.17, 92.86)	4.80 (0.18, 131.64)	1.72 (0.16, 17.42)	4.03 (0.27, 60.91)	0.38 (0.04, 3.99)	0.68 (0.08, 6.06)	3.44 (0.16, 71.26)	0.72 (0.01, 82.08)	0.90 (0.03, 24.27)	0.48 (0.03, 7.54)	TACE+PVE	2.10 (0.09, 53.20)	2.11 (0.20, 22.06)	6.79 (0.65, 72.08)	2.26 (0.22, 23.23)	3.20 (0.17, 58.89)
0.83 (0.06, 12.68)	1.76 (0.05, 60.22)	2.99 (0.17, 57.98)	1.77 (0.09, 33.42)	0.71 (0.03, 18.89)	0.77 (0.02, 36.43)	0.04 (0.00, 0.50)	0.27 (0.01, 6.31)	0.59 (0.05, 6.66)	0.35 (0.02, 6.78)	1.70 (0.11, 28.69)	0.75 (0.04, 14.21)	2.72 (0.15, 46.90)	1.80 (0.07, 44.42)	2.31 (0.09, 66.50)	0.81 (0.07, 9.81)	1.93 (0.11, 33.41)	0.18 (0.01, 2.07)	0.33 (0.03, 3.30)	1.61 (0.06, 38.83)	0.34 (0.00, 39.58)	0.42 (0.01, 12.35)	0.22 (0.01, 4.22)	0.48 (0.02, 11.42)	TACE+RFA	1.00 (0.08, 12.48)	3.27 (0.26, 41.06)	1.06 (0.09, 12.28)	1.47 (0.07, 33.79)
0.85 (0.20, 3.71)	1.78 (0.11, 29.69)	3.01 (0.43, 22.31)	1.80 (0.25, 12.86)	0.71 (0.06, 8.71)	0.77 (0.04, 20.21)	0.04 (0.01, 0.12)	0.28 (0.03, 2.29)	0.60 (0.23, 1.52)	0.35 (0.05, 2.52)	1.75 (0.30, 9.76)	0.77 (0.09, 5.95)	2.73 (0.43, 16.85)	1.78 (0.18, 19.56)	2.28 (0.18, 28.84)	0.81 (0.27, 2.36)	1.91 (0.34, 11.38)	0.18 (0.06, 0.52)	0.33 (0.13, 0.78)	1.62 (0.15, 16.46)	0.34 (0.01, 24.93)	0.43 (0.03, 5.66)	0.22 (0.04, 1.37)	0.47 (0.05, 5.02)	1.00 (0.08, 12.34)	TACE+RT	3.25 (0.96, 11.07)	1.06 (0.32, 3.43)	1.51 (0.19, 11.79)

0.26 (0.06, 1.19)	0.56 (0.03, 8.72)	0.93 (0.13, 6.94)	0.55 (0.08, 4.09)	0.22 (0.02, 2.75)	0.23 (0.01, 6.44)	0.01 (0.00, 0.04)	0.09 (0.01, 0.70)	0.18 (0.06, 0.52)	0.11 (0.01, 0.81)	0.53 (0.09, 3.35)	0.24 (0.03, 1.84)	0.84 (0.13, 5.45)	0.55 (0.05, 6.07)	0.70 (0.06, 9.36)	0.25 (0.07, 0.81)	0.59 (0.10, 3.62)	0.06 (0.02, 0.15)	0.10 (0.04, 0.25)	0.50 (0.04, 5.25)	0.11 (0.00, 6.97)	0.13 (0.01, 1.75)	0.07 (0.01, 0.45)	0.15 (0.01, 1.53)	0.31 (0.02, 3.83)	0.31 (0.09, 1.05)	TACE+Sorafenib	0.33 (0.10, 1.07)	0.46 (0.06, 3.56)
0.80 (0.19, 3.41)	1.70 (0.11, 27.55)	2.81 (0.41, 20.94)	1.69 (0.24, 12.25)	0.67 (0.06, 8.09)	0.71 (0.03, 18.71)	0.04 (0.01, 0.11)	0.27 (0.03, 2.20)	0.57 (0.21, 1.53)	0.33 (0.04, 2.39)	1.61 (0.28, 9.38)	0.72 (0.09, 5.73)	2.58 (0.40, 16.55)	1.69 (0.16, 19.07)	2.17 (0.17, 27.57)	0.77 (0.23, 2.52)	1.82 (0.31, 10.92)	0.17 (0.07, 0.43)	0.31 (0.13, 0.73)	1.54 (0.14, 15.82)	0.33 (0.01, 22.26)	0.40 (0.03, 5.52)	0.21 (0.03, 1.33)	0.44 (0.04, 4.55)	0.94 (0.08, 11.73)	0.94 (0.29, 3.12)	3.07 (0.93, 10.40)	TARE	1.42 (0.23, 8.70)
0.57 (0.06, 5.13)	1.19 (0.05, 30.72)	2.01 (0.15, 26.88)	1.18 (0.09, 15.40)	0.46 (0.02, 9.69)	0.49 (0.01, 18.95)	0.03 (0.00, 0.20)	0.19 (0.01, 2.70)	0.40 (0.06, 2.75)	0.23 (0.02, 3.20)	1.14 (0.10, 13.92)	0.51 (0.03, 7.29)	1.80 (0.14, 22.80)	1.19 (0.07, 21.98)	1.51 (0.07, 35.41)	0.54 (0.07, 4.16)	1.27 (0.11, 15.46)	0.12 (0.02, 0.74)	0.22 (0.03, 1.40)	1.06 (0.06, 18.55)	0.23 (0.00, 21.28)	0.28 (0.01, 6.12)	0.15 (0.01, 1.77)	0.31 (0.02, 6.00)	0.68 (0.03, 14.41)	0.66 (0.08, 5.15)	2.17 (0.28, 16.74)	0.70 (0.11, 4.29)	TARE+Sorafenib

BCLC Stage D. 1-year overall survival

HAC	0.17 (0.01, 4.95)	0.19 (0.02, 1.52)
5.93 (0.20, 195.80)	RFA	1.10 (0.08, 13.62)
5.22 (0.66, 62.75)	0.91 (0.07, 13.03)	TACE

BCLC Stage D. 1-year overall survival

LT	0.61 (0.00, 724.43)	0.00 (0.00, 0.01)
1.63 (0.00, 1982.19)	PEI	0.00 (0.00, 0.01)
8.90E+23 (191.03, 1.72E+62)	4.80E+23 (71.51, 1.20E+62)	SC

Supplementary Table S5. Results of Node-splitting Models for the Test of Difference Between Direct and Indirect Effect.

Items	Comparison	Direct Effect	Indirect Effect	Overall	*P* value
**BCLC Stage 0. 1-year overall survival**					
	PEI, SR	-0.43 (-2.80, 1.99)	0.60 (-0.90, 2.53)	0.36 (-0.92, 1.88)	0.40
**BCLC Stage 0. 3-year overall survival**					
	PEI, SR	-0.18 (-1.86, 1.50)	0.32 (-0.75, 1.47)	0.24 (-0.69, 1.19)	0.57
**BCLC Stage 0. 5-year overall survival**					
	PEI, SR	-0.53 (-2.43, 1.36)	-0.10 (-1.37, 1.19)	-0.06 (-1.14, 1.05)	0.68
**BCLC Stage A. 1-year overall survival**					
	LT, TACE	0.12 (-2.02, 2.45)	-0.68 (-1.65, 0.25)	-0.54 (-1.50, 0.33)	0.48
	MWA, RFA	0.44 (-0.73, 1.58)	-0.42 (-1.69, 0.80)	0.06 (-0.78, 0.90)	0.31
	MWA, SR	-0.21 (-1.51, 0.98)	0.64 (-0.53, 1.87)	0.25 (-0.61, 1.09)	0.31
	PEI, RFA	-0.14 (-1.04, 0.76)	-0.43 (-1.14, 0.31)	-0.34 (-0.96, 0.30)	0.59
	RFA, SR	0.22 (-0.06, 0.50)	-0.04 (-0.71, 0.63)	0.18 (-0.08, 0.44)	0.47
	RFA, TACE	0.16 (-0.62, 0.92)	-0.08 (-0.75, 0.59)	0.01 (-0.50, 0.51)	0.65
	RFA, TACE+RFA	0.48 (-0.66, 1.66)	0.89 (-0.03, 1.85)	0.70 (0.00, 1.44)	0.58
	SC, SR	1.84 (0.87, 2.89)	2.51 (1.10, 3.99)	1.94 (1.06, 2.89)	0.41
	SC, TACE	2.30 (1.00, 3.76)	1.66 (0.51, 2.84)	1.77 (0.84, 2.75)	0.44
	SR, TACE	-0.22 (-0.85, 0.43)	-0.21 (-1.00, 0.59)	-0.17 (-0.66, 0.31)	0.99
	SR, TACE+RFA	0.68 (-0.30, 1.67)	0.33 (-0.66, 1.38)	0.52 (-0.18, 1.24)	0.62
	TACE, TACE+RFA	0.83 (-0.47, 2.23)	0.60 (-0.32, 1.56)	0.69 (-0.08, 1.50)	0.77
**BCLC Stage A. 3-year overall survival**					
	LT, TACE	-1.52 (-3.40, 0.31)	-1.55 (-2.32, -0.78)	-1.50 (-2.25, -0.76)	0.97
	MWA, RFA	0.64 (-0.16, 1.43)	-0.14 (-0.90, 0.63)	0.23 (-0.34, 0.78)	0.17
	MWA, SR	0.35 (-0.37, 1.05)	1.13 (0.32, 1.95)	0.71 (0.15, 1.27)	0.15
	PEI, RFA	-0.49 (-1.60, 0.60)	-0.27 (-0.94, 0.39)	-0.30 (-0.91, 0.30)	0.73
	RFA, SR	0.51 (0.29, 0.73)	0.33 (-0.21, 0.87)	0.48 (0.28, 0.69)	0.55
	RFA, TACE	-0.22 (-0.84, 0.40)	0.08 (-0.48, 0.62)	-0.07 (-0.47, 0.33)	0.47
	RFA, TACE+RFA	0.47 (-0.41, 1.37)	0.02 (-0.60, 0.63)	0.17 (-0.34, 0.68)	0.41
	SC, SR	2.00 (1.06, 2.97)	2.39 (1.12, 3.81)	2.08 (1.21, 2.96)	0.57
	SC, TACE	1.85 (0.62, 3.14)	1.44 (0.42, 2.49)	1.53 (0.63, 2.46)	0.56
	SR, TACE	-0.65 (-1.17, -0.15)	-0.40 (-1.01, 0.21)	-0.55 (-0.94, -0.16)	0.53
	SR, TACE+RFA	-0.16 (-0.81, 0.50)	-0.50 (-1.25, 0.24)	-0.31 (-0.80, 0.18)	0.49
	TACE, TACE+RFA	-0.09 (-1.03, 0.87)	0.36 (-0.35, 1.05)	0.24 (-0.32, 0.80)	0.45
**BCLC Stage A. 5-year overall survival**					

LT, PEI	-1.91 (-3.43, -0.40)	-1.28 (-2.19, -0.38)	-1.46 (-2.30, -0.65)	0.44
LT, RFA	-0.17 (-1.66, 1.24)	-1.57 (-2.22, -0.95)	-1.50 (-2.15, -0.86)	0.06
LT, SC	-2.79 (-4.43, -1.17)	-3.22 (-4.54, -2.00)	-3.01 (-4.01, -2.01)	0.65
LT, SR	-1.14 (-1.79, -0.53)	-0.62 (-1.77, 0.51)	-1.04 (-1.66, -0.43)	0.38
LT, TACE	-1.91 (-3.14, -0.70)	-1.77 (-2.56, -1.03)	-1.79 (-2.51, -1.11)	0.82
MWA, RFA	-0.45 (-2.57, 1.50)	-0.17 (-0.96, 0.59)	-0.20 (-0.92, 0.54)	0.79
MWA, SR	0.29 (-0.46, 1.03)	0.03 (-2.10, 2.01)	0.26 (-0.43, 0.97)	0.80
PEI, RFA	-0.10 (-1.02, 0.82)	-0.04 (-0.65, 0.61)	-0.04 (-0.61, 0.55)	0.92
PEI, SC	-1.04 (-2.48, 0.35)	-1.76 (-3.10, -0.53)	-1.54 (-2.49, -0.61)	0.44
PEI, SR	0.39 (-0.22, 1.01)	0.24 (-0.59, 1.11)	0.42 (-0.12, 0.99)	0.78
PEI, TACE	-0.35 (-1.65, 0.95)	-0.25 (-0.95, 0.44)	-0.33 (-0.99, 0.33)	0.88
RFA, SC	-2.04 (-3.54, -0.61)	-1.73 (-2.90, -0.63)	-1.51 (-2.35, -0.69)	0.73
RFA, SR	0.38 (0.16, 0.62)	0.95 (0.39, 1.51)	0.46 (0.25, 0.68)	0.07
RFA, TACE	-0.26 (-0.87, 0.33)	-0.41 (-1.03, 0.21)	-0.30 (-0.71, 0.12)	0.74
RFA, TACE+RFA	0.80 (-0.12, 1.75)	-0.15 (-0.96, 0.66)	0.21 (-0.38, 0.81)	0.12
SC, SR	1.83 (0.92, 2.74)	2.06 (1.08, 3.07)	1.96 (1.16, 2.79)	0.66
SC, TACE	1.07 (0.09, 2.12)	1.29 (0.34, 2.27)	1.21 (0.35, 2.08)	0.71
SR, TACE	-0.83 (-1.32, -0.35)	-0.38 (-1.13, 0.35)	-0.75 (-1.16, -0.36)	0.30
SR, TACE+RFA	-0.59 (-1.36, 0.18)	0.20 (-0.68, 1.10)	-0.25 (-0.83, 0.34)	0.19
TACE, TACE+RFA	0.67 (-0.71, 2.00)	0.28 (-0.48, 1.05)	0.51 (-0.16, 1.18)	0.62
**BCLC Stage B. 1-year overall survival**				
SC, SR	2.17 (1.15, 3.26)	2.66 (1.62, 3.78)	2.31 (1.56, 3.15)	0.48
SC, Sorafenib	0.41 (-1.30, 2.16)	2.69 (0.90, 4.57)	1.35 (0.05, 2.68)	0.06
SC, TACE	1.98 (1.05, 2.97)	1.03 (0.01, 2.00)	1.48 (0.73, 2.29)	0.13
SR, TACE	-0.89 (-1.33, -0.46)	-0.35 (-2.10, 1.33)	-0.83 (-1.26, -0.45)	0.53
**BCLC Stage B. 3-year overall survival**				
SC, SR	2.25 (0.78, 3.77)	2.75 (1.15, 4.63)	2.42 (1.36, 3.66)	0.61
SC, Sorafenib	-21.86 (-85.64, -0.87)	25.03 (4.11, 85.74)	1.60 (-0.36, 3.72)	0.09
SC, TACE	2.23 (0.74, 4.00)	0.60 (-1.00, 1.96)	1.31 (0.25, 2.57)	0.07
SR, TACE	-1.16 (-1.72, -0.62)	0.22 (-2.60, 3.91)	-1.10 (-1.64, -0.58)	0.34
**BCLC Stage C. 1-year overall survival**				
HAC, HAC+Sorafenib	0.43 (-2.03, 2.94)	1.15 (-1.54, 3.86)	0.71 (-1.11, 2.55)	0.68
HAC, SC	-2.33 (-4.39, -0.60)	-3.50 (-5.16, -1.92)	-2.97 (-4.30, -1.74)	0.35
HAC, SCT	-2.23 (-6.04, 0.65)	-0.24 (-3.09, 2.55)	-1.08 (-3.15, 0.90)	0.33
HAC, Sorafenib	-1.64 (-3.43, 0.10)	-1.47 (-3.31, 0.26)	-1.54 (-2.78, -0.30)	0.89

HAC+Sorafenib, TACE	-1.99 (-4.30, 0.31)	-1.30 (-4.23, 1.57)	-1.67 (-3.47, 0.07)	0.70
SC, SR	2.66 (1.81, 3.61)	2.70 (1.74, 3.71)	2.61 (1.95, 3.32)	0.95
SC, SR+TACE	2.20 (0.83, 3.65)	3.69 (2.59, 4.87)	2.93 (1.97, 3.90)	0.09
SC, Sorafenib	1.04 (-0.41, 2.51)	1.66 (0.64, 2.77)	1.44 (0.59, 2.30)	0.47
SC, TACE	2.25 (1.50, 3.07)	1.64 (0.81, 2.54)	2.01 (1.38, 2.70)	0.27
SC, TACE+Sorafenib	5.67 (3.11, 8.33)	3.85 (2.71, 5.07)	4.31 (3.25, 5.43)	0.20
SCT, SR	0.37 (-2.24, 2.89)	0.88 (-1.60, 3.34)	0.72 (-1.19, 2.73)	0.75
SR, SR+TACE	0.91 (-0.02, 1.85)	-1.42 (-3.01, 0.04)	0.31 (-0.51, 1.14)	0.07
SR, Sorafenib	-1.26 (-3.74, 1.21)	-1.22 (-2.17, -0.29)	-1.18 (-2.04, -0.32)	0.97
SR, TACE	-0.78 (-1.45, -0.08)	-0.28 (-1.23, 0.66)	-0.59 (-1.18, -0.03)	0.40
SR, TACE+RT	0.17 (-1.98, 2.26)	0.60 (-0.48, 1.69)	0.51 (-0.42, 1.51)	0.73
SR+PVC, TACE	-2.32 (-5.17, 0.33)	-1.49 (-3.14, 0.20)	-1.65 (-3.25, -0.05)	0.59
SR+RT, SR+TACE	-0.65 (-3.09, 1.77)	-1.63 (-3.54, 0.25)	-1.22 (-2.96, 0.49)	0.49
SR+TACE, Sorafenib	-0.64 (-4.36, 2.39)	-1.42 (-2.48, -0.36)	-1.49 (-2.53, -0.44)	0.66
SR+TACE, TACE	0.16 (-1.13, 1.48)	-1.30 (-2.22, -0.36)	-0.91 (-1.76, -0.04)	0.06
SR+TACE, TACE+RT	-0.43 (-2.47, 1.69)	0.47 (-0.82, 1.80)	0.20 (-0.87, 1.30)	0.45
SR+TACE, TACE+Sorafenib	4.31 (2.04, 6.86)	0.92 (-0.38, 2.20)	1.39 (0.19, 2.60)	0.10
Sorafenib , TACE	1.41 (0.17, 2.65)	0.25 (-0.52, 1.05)	0.58 (-0.15, 1.32)	0.10
Sorafenib , TACE+RT	3.10 (1.46, 4.80)	1.05 (-0.20, 2.32)	1.70 (0.69, 2.75)	0.06
Sorafenib , TACE+Sorafenib	2.44 (1.13, 3.77)	3.22 (1.74, 4.79)	2.88 (1.88, 3.90)	0.42
Sorafenib , TARE	1.25 (-0.03, 2.53)	2.24 (0.99, 3.53)	1.75 (0.88, 2.67)	0.28
Sorafenib , TARE+Sorafenib	1.51 (-1.04, 4.01)	2.89 (0.32, 5.49)	2.12 (0.32, 3.96)	0.45
TACE, TACE+RT	0.70 (-0.44, 1.89)	1.62 (0.27, 3.05)	1.12 (0.26, 2.02)	0.30
TACE, TACE+Sorafenib	2.40 (1.32, 3.52)	0.85 (-0.85, 2.51)	2.30 (1.39, 3.24)	0.13
TACE, TARE	1.64 (0.57, 2.70)	0.31 (-1.18, 1.78)	1.17 (0.31, 2.04)	0.14
TARE, TARE+Sorafenib	0.95 (-1.43, 3.50)	-0.38 (-3.02, 2.28)	0.38 (-1.42, 2.18)	0.46
